# Mapping the structural organization of the brain in conduct disorder: replication of findings in two independent samples

**DOI:** 10.1111/jcpp.12581

**Published:** 2016-06-15

**Authors:** Graeme Fairchild, Nicola Toschi, Kate Sully, Edmund J.S. Sonuga‐Barke, Cindy C. Hagan, Stefano Diciotti, Ian M. Goodyer, Andrew J. Calder, Luca Passamonti

**Affiliations:** ^1^ Academic Unit of PsychologyUniversity of SouthamptonSouthamptonUK; ^2^ Department of PsychiatryUniversity of CambridgeCambridgeUK; ^3^ Department of Biomedicine and PreventionUniversity of Rome “Tor Vergata”RomeItaly; ^4^ Martinos Center for Biomedical ImagingBostonMAUSA; ^5^ Harvard Medical SchoolBostonMAUSA; ^6^ Department of Experimental, Clinical & Health PsychologyGent UniversityGentBelgium; ^7^ Department of PsychologyColumbia UniversityNew YorkNYUSA; ^8^ Department of Electrical, Electronic, and Information EngineeringUniversity of BolognaBolognaItaly; ^9^ Medical Research CouncilCognition and Brain Sciences UnitCambridgeUK; ^10^ Institute of Bioimaging and Molecular PhysiologyNational Research CouncilCatanzaroItaly; ^11^ Department of Clinical NeurosciencesUniversity of CambridgeCambridgeUK

**Keywords:** Cortical thickness, structural covariance, conduct disorder, antisocial behavior, developmental taxonomic theory

## Abstract

**Background:**

Neuroimaging methods that allow researchers to investigate structural covariance between brain regions are increasingly being used to study psychiatric disorders. Structural covariance analyses are particularly well suited for studying disorders with putative neurodevelopmental origins as they appear sensitive to changes in the synchronized maturation of different brain regions. We assessed interregional correlations in cortical thickness as a measure of structural covariance, and applied this method to investigate the coordinated development of different brain regions in conduct disorder (CD). We also assessed whether structural covariance measures could differentiate between the childhood‐onset (CO‐CD) and adolescence‐onset (AO‐CD) subtypes of CD, which may differ in terms of etiology and adult outcomes.

**Methods:**

We examined interregional correlations in cortical thickness in male youths with CO‐CD or AO‐CD relative to healthy controls (HCs) in two independent datasets. The age range in the Cambridge sample was 16–21 years (mean: 18.0), whereas the age range of the Southampton sample was 13–18 years (mean: 16.7). We used FreeSurfer to perform segmentations and applied structural covariance methods to the resulting parcellations.

**Results:**

In both samples, CO‐CD participants displayed a strikingly higher number of significant cross‐cortical correlations compared to HC or AO‐CD participants, whereas AO‐CD participants presented fewer significant correlations than HCs. Group differences in the strength of the interregional correlations were observed in both samples, and each set of results remained significant when controlling for IQ and comorbid attention‐deficit/hyperactivity disorder symptoms.

**Conclusions:**

This study provides new evidence for quantitative differences in structural brain organization between the CO‐CD and AO‐CD subtypes, and supports the hypothesis that both subtypes of CD have neurodevelopmental origins.

## Introduction

Structural covariance is an important property of brain organization. Brain regions that develop together show higher covariance in neuroanatomical measures, such as cortical thickness, than regions that develop according to different maturational schedules (Alexander‐Bloch, Giedd, & Bullmore, 2013). Recently, there has been increasing interest in applying structural covariance methods to investigate psychiatric disorders with putative neurodevelopmental origins, such as autism (Dziobek, Bahnemann, Convit, & Heekeren, 2010), and attention‐deficit/hyperactivity disorder (ADHD; Li et al., 2015). In the present study, we employed structural covariance methods based on cortical thickness data to compare youths with conduct disorder (CD) and typically developing individuals in terms of the overall number of significant interregional correlations in cortical thickness and test for group differences in the relative strength of these correlations.

We also assessed for differences between the two main subtypes of CD – that is the childhood‐onset subtype of CD (CO‐CD) and the adolescence‐onset subtype (AO‐CD; American Psychiatric Association, 2013). According to the developmental taxonomic theory, CO‐CD is a neurodevelopmental disorder, whereas AO‐CD is an exaggerated form of teenage rebellion in which individuals imitate the behavior of antisocial peers (Moffitt, 1993). However, contrary to this theory, we found that both forms of CD are associated with alterations in brain structure and function (Fairchild et al., 2011; Passamonti et al., 2010). Consequently, we reformulated the developmental taxonomic theory to create a new model of CD which proposes that CO‐CD and AO‐CD differ on a quantitative, rather a qualitative, basis (Fairchild, van Goozen, Calder, & Goodyer, 2013). Nevertheless, it remains to be determined whether brain‐based measures are able to discriminate between these CD subtypes, as we previously found little or no evidence for differences between CO‐CD and AO‐CD in brain function and structure (Fairchild et al., 2011; Passamonti et al., 2010). The neurodevelopmental changes that have been described as occurring in childhood differ quantitatively and qualitatively from those observed in adolescence (e.g. childhood is associated with progressive cellular maturational events such as synaptogenesis, while adolescence is characterized by synaptic pruning Giedd et al., 1999); hence, it is of interest to investigate whether CO‐CD and AO‐CD are associated with distinct alterations in structural covariance that may in turn reflect different neurodevelopmental influences. Structural covariance methods may be particularly informative in this respect, given their sensitivity to changes in the coordinated development of brain regions across the entire cortex.

The notion that neurodevelopmental abnormalities may contribute to the pathophysiology of CD has also been supported by recent morphometric studies showing that CD youths, relative to age‐matched healthy controls (HCs), display alterations in cortical thickness, folding, and surface area (Fairchild et al., 2015; Hyatt, Haney‐Caron, & Stevens, 2012), which are aspects of cortical structure that differ in etiology and developmental trajectories (Panizzon et al., 2009). Likewise, it is possible that CD youths, relative to HCs, present significant changes in the coordinated growth of multiple cortical areas across the brain, rather than just a specific region or network. This is because the genetic and environmental factors that increase risk for CD may exert similar neurotrophic influences across interconnected brain regions or areas that subserve similar functions (Lerch et al., 2006). Furthermore, CD‐related genetic and environmental risk factors may alter brain developmental trajectories in different ways depending on whether they are activated or occur in childhood or adolescence.

To our knowledge, this study is the first to test for changes in structural covariance in youths with CD relative to HCs. We applied a method developed by Lerch and colleagues known as ‘Mapping Anatomical Correlations Across Cerebral Cortex’ (MACACC; Lerch et al., 2006) to analyze cortical thickness data collected in two independent samples of participants (*n* = 83 recruited in Cambridge and *n* = 69 recruited in Southampton). Critically, the MACACC approach assesses for interregional correlations in cortical thickness across the entire cortex, enabling us to test whether CD is associated with global changes in brain structure. Previous studies have shown that neurodevelopmental disorders such as autism may be associated with widespread changes in structural covariance networks (Alexander‐Bloch et al., 2013), so one of our key aims was to test whether CD is associated with similar global changes in the structural organization of the brain.

We also hypothesized that youths with CD would display increases or reductions in the strength of interregional correlations in cortical thickness relative to HCs. This hypothesis was guided by previous work showing both increases and reductions in the strength of interregional correlations in adults with antisocial personality disorder, relative to age‐matched HCs (Yang et al., 2012). Furthermore, we predicted that youths with CO‐CD would show more pronounced alterations in interregional correlations in cortical thickness than youths with AO‐CD, given that CO‐CD is typically more severe and linked to less favorable adult outcomes (Burt, Donnellan, Iacono, & McGue, 2011). Finally, we hypothesized that the group differences in correlation strength would be identified within and across a number of prefrontal and temporal regions previously implicated in the pathophysiology of antisocial behavior (Yang et al., 2012). These hypotheses were tested in two independent samples recruited in separate locations, using similar psychiatric assessment procedures.

## Participants

Fifty‐eight male adolescents and young adults with CD and 25 sex‐ and age‐matched HCs (age range: 16–21 years) were recruited at Cambridge University between 2007 and 2010. Part of this sample was included in earlier neuroimaging studies published by our group (Fairchild et al., 2011; Passamonti et al., 2010). An independent sample of 37 CD and 32 HC participants (all male; age range: 13–18 years) was recruited at Southampton University between 2012 and 2014.

At both sites, CD participants were recruited from pupil referral units and Youth Offending Services (YOSs), whereas HCs were recruited from mainstream schools and colleges. At schools and colleges, participants were contacted by sending an information pack about the study from the school/college to their homes. At pupil referral units and YOSs, a member of staff (usually their keyworker) described the study to potential volunteers and asked them whether they were interested in taking part. If they were interested in participating, they either sent back reply slips or gave permission for the staff member to pass on their contact details to the research team. Once we received reply slips or contact details, we arranged to visit the participants' homes to carry out separate semistructured diagnostic interviews with them and their primary caregiver. The Suffolk National Health Service Research Ethics Committee approved the Cambridge study, whereas the University of Southampton's Ethics Committee and Research Governance Office approved the Southampton study. Written informed consent was obtained from all participants and their parents.

Conduct disorder was assessed in the same way in both samples: participants and their parents underwent separate semistructured diagnostic interviews using the Kiddie‐Schedule for Affective Disorders and Schizophrenia‐Present and Lifetime Version (K‐SADS‐PL; Kaufman et al., 1997). Diagnoses were reached by combining information from both interviews. Participants were classified as having CO‐CD if they or their parents reported that at least one CD symptom and functional impairment was present before age 10. Alternatively, if the individual only developed CD symptoms after age 10, an AO‐CD diagnosis was given. According to these criteria, 33 participants were classified as having CO‐CD and 25 as having AO‐CD in the Cambridge sample, whereas 23 individuals had CO‐CD and 14 had AO‐CD in the Southampton sample.

The exclusion criteria applied in Cambridge and Southampton were broadly similar: (a) presence of serious physical or psychiatric illnesses (e.g. autism, schizophrenia, bipolar disorder), as disclosed in the K‐SADS‐PL interview; and (b) any contraindication to brain scanning (e.g. claustrophobia or metal in the body). In Cambridge, we also excluded CD participants with IQs < 85, as estimated using the two subtest version of the Wechsler Abbreviated Scale of Intelligence (Wechsler, 1999), whereas in Southampton the cutoff on the same instrument was IQ < 75. In an attempt to match groups in IQ, we only included HCs with IQs < 115 in both samples. We administered the full ADHD supplement of the K‐SADS‐PL to participants in both samples to comprehensively assess for all 18 of the DSM‐IV symptoms of ADHD and evaluate the impact of threshold and subthreshold ADHD comorbidity on the key findings by regressing out the contribution of ADHD symptoms when assessing for significant interregional correlations within each group, as well as group differences in interregional correlation strength. At both sites, we obtained data on psychopathic traits using the self‐report Youth Psychopathic traits Inventory (Andershed, Kerr, Stattin, & Levander, 2002). In the Cambridge sample, we obtained data on participants' socioeconomic status using the ACORN geodemographic tool, which is based on UK postcodes (http://acorn.caci.co.uk/).

## Magnetic resonance imaging (MRI)

In Cambridge, structural MRI data were acquired on a 3‐Tesla Siemens Tim‐Trio scanner at the Medical Research Council Cognition and Brain Sciences Unit. The following parameters were used: voxel size = 1 × 1 × 1 mm, repetition time = 2250 ms; echo time = 2.99 ms; flip angle = 9°. Total scanning time was 4 min and 16 s.

In Southampton, the structural MRI data were acquired on a 1.5‐Tesla Siemens Magnetom scanner at the Southampton General Hospital using the following parameters: voxel size = 1.2 × 1.2 × 1.2 mm, repetition time = 2400 ms, echo time = 3.62 ms, flip angle = 8°. Total scanning time was 7 min and 41 s.

Stringent quality control procedures were adopted during data collection and preprocessing in both samples. Specifically, all structural images were checked immediately after acquisition for movement artifacts, and repeated until a high‐quality T1‐weighted image was available. Before including T1‐weighted images in the study, they were carefully reviewed by two coauthors (G.F., L.P.) and an experienced radiographer who was blind to group status and the researchers' assessments. Additional visual inspection after cortical surface reconstruction was manually performed by another coauthor (N.T.), who is an expert in advanced neuroimaging analyses. To minimize discomfort and reduce motion artifacts, the structural MRI data were collected at the beginning of each scanning session.

## MACACC approach

Quantification of cortical thickness values was performed using FreeSurfer v.5.3.0 (http://surfer.nmr.mgh.harvard.edu) (Dale, Fischl, & Sereno, 1999; Fischl & Dale, 2000). This method involves vertex‐wise reconstruction of the white matter and pial surface and parcellation of the cortex into 34 regions of interest (ROIs) per hemisphere according to the Desikan–Killiany atlas (Desikan et al., 2006). An average cortical thickness value was then extracted for each ROI by reconstructing the gray–white matter boundary and the cortical surface; the distance between these surfaces was calculated individually at each point/vertex across the cortical mantle (see Supporting information for further details).

In both samples, the cortical thickness data were mean‐centered across regions within each participant, before computing the interregional correlations and testing for group differences in correlation strength. This was done to limit the possibility that the findings were driven by scaling effects. Following this step, we regressed out the effects of age, IQ, and ADHD symptoms before computing the interregional correlation coefficients in the Cambridge sample. This enabled us to adjust for these variables which differed between the CD and HC groups (Table 1). Likewise, in the Southampton sample, we mean‐centered cortical thickness across regions within each participant to adjust for possible scaling effects and regressed out the contribution of IQ and ADHD symptoms before computing the interregional correlations. Age was not included as a covariate in this case, as the groups were deliberately matched on this variable (Table 1). Nevertheless, for completeness and to show the impact of these variables on the structural covariance findings, the interregional correlation results that were obtained without controlling for these variables are reported in Supporting information. To confirm that our results were not attributable to group differences in brain size, we also repeated all analyses regressing out estimated total intracranial volume (eTIV) along with demographic and clinical variables.

**Table 1:** Demographic and clinical characteristics of the participants

Cambridge sample	HCs (*n* = 25)		CO‐CD (*n* = 33)		AO‐CD (*n* = 25)		One‐way ANOVA analyses
Measure	Mean	*SD*	Mean	*SD*	Mean	*SD*	
Age (years)	18.5	1.1	17.8	1.1	17.9	1.1	*F* = 3.12; *p* = .05
Verbal IQ	99.0	13.1	89.6	14.0	96.0	16.9	*F* = 3.13; *p* = .05
Performance IQ	107.5	11.7	104.5	10.7	105.6	12.7	*F* = 0.5; *p* = .62
Psychopathic traits (YPI)	98.5	13.2	124.1	21.0	123.7	17.7	*F* = 17.5; *p* < .001
Lifetime/ever CD symptoms	0.4	0.6	9.0	1.8	7.4	2.5	*F* = 172.3; *p* < .0001
Lifetime/ever ADHD symptoms	2.5	2.3	9.0	4.7	6.0	4.3	*F* = 18.7; *p* < .001
SES (ACORN class)	*n*	%	*n*	%	*N*	%	χ² (exact test)
Wealthy achievers (1)	4	16.0	0	0.0	2	8.0	χ^2^ = 13.7, *p* = .09
Urban prosperity (2)	7	28.0	2	6.1	6	24.0	
Comfortably off (3)	6	24.0	13	39.4	6	24.0	
Moderate means (4)	2	8.0	4	12.1	1	4.0	
Hard‐pressed (5)	6	24.0	14	42.4	10	40.0	
Comorbid diagnoses	*n*	%	*n*	%	*N*	%	χ² (exact test)
Number with comorbid ADHD^a^	–	–	11	33.3	3	12.0	
Number with comorbid MDD^a^	–	–	3	9.0	2	8.0	
Regular use of:							
Tobacco	7	28.0	28	84.8	20	80.0	χ^2^ = 23.6, *p* < .001
Alcohol	13	52.0	20	60.6	20	80.0	χ^2^ = 4.5, *p* = .11
Cannabis	4	16.0	19	57.6	16	64.0	χ^2^ = 14.0, *p* = .001
Current medication use	*n*	%	*n*	%	*N*	%	
Methylphenidate	–	–	2	6.0	0	0	
Southampton sample	HCs (*n* = 32)		CO‐CD (*n* = 23)		AO‐CD (*n* = 14)		
Measure	Mean	*SD*	Mean	*SD*	Mean	*SD*	One‐way ANOVA analyses
Age (years)	16.6	1.1	16.7	1.4	16.8	1.2	*F* = 0.10; *p* = .91
Estimated full‐scale IQ	103.4	10.0	94.7	12.4	89.4	7.7	*F* = 9.89; *p* < .001
Psychopathic traits (YPI)^b^	101.7	16.3	125.5	22.3	120.4	18.1	*F* = 11.57; *p* < .001
Lifetime/ever CD symptoms	0.3	0.6	9.4	2.0	7.6	1.7	*F* = 300.8; *p* < .0001
Lifetime/ever ADHD symptoms	0.7	1.4	8.1	4.9	6.4	3.4	*F* = 36.8; *p* < .001
Comorbid diagnoses	*n*	%	*n*	%	*N*	%	*χ*² (exact test)
Number with comorbid ADHD^a^	–	–	6	26.1	2	14.3	
Number with comorbid MDD^a^	–	–	2	8.7	0	0	
Current medication use	*n*	%	*n*	%	*N*	%	
Selective serotonin reuptake inhibitors	–	–	1	4.3	2	14.2	
Methylphenidate	–	–	2	8.6	0	0	

ADHD, attention‐deficit/hyperactivity disorder; AO‐CD, adolescence‐onset conduct disorder; CO‐CD, childhood‐onset conduct disorder; HCs, healthy controls; IQ, intelligence quotient; *SD*, standard deviation; SES, socioeconomic status; YPI, Youth Psychopathic traits Inventory. ACORN is a geodemographic tool for assessing socioeconomic status using UK postcodes.

a A current psychiatric disorder was an exclusion criterion for the control group.

b YPI data were unavailable for one control subject.

Next, cortical thickness correlation matrices were generated within each group and in each sample by computing the Pearson's correlation coefficients (*R*‐values) between each pair of regions across subjects with an associated *p*‐value that was corrected for multiple comparisons using a false‐discovery‐rate (FDR) procedure. To test for significant differences across groups, and to avoid a priori assumptions about the distribution of differences between group‐wise *R*‐values for each correlation, the null distribution was built using a random sampling technique (1 × 10^6^ samples – see Supporting information for details about the procedure) and the resulting *p*‐value was corrected for multiple comparisons (Benjamini‐Hochberg's correction, *p* < .05, FDR).

Finally, to assess the degree of comparability between the results obtained with the Cambridge and Southampton samples, we produced correlation matrices illustrating the overlap between the samples in terms of the significant interregional correlations identified within each group (i.e. HCs, CO‐CD, and AO‐CD).

## Results

### Participant characteristics

In both samples, youths with CD had higher levels of psychopathic traits and reported more CD and ADHD symptoms than HCs (Table 1). In the Cambridge sample, post hoc tests comparing the CO‐CD and AO‐CD subgroups revealed that CO‐CD youths endorsed more CD (*p* = .03) and ADHD symptoms (*p* = .04) than AO‐CD participants. Likewise, in the Southampton sample, youths with CO‐CD tended to display more CD and ADHD symptoms than AO‐CD youths, although these differences were not statistically significant (*p*s > .2).

### Structural covariance results

**Figure 1:**
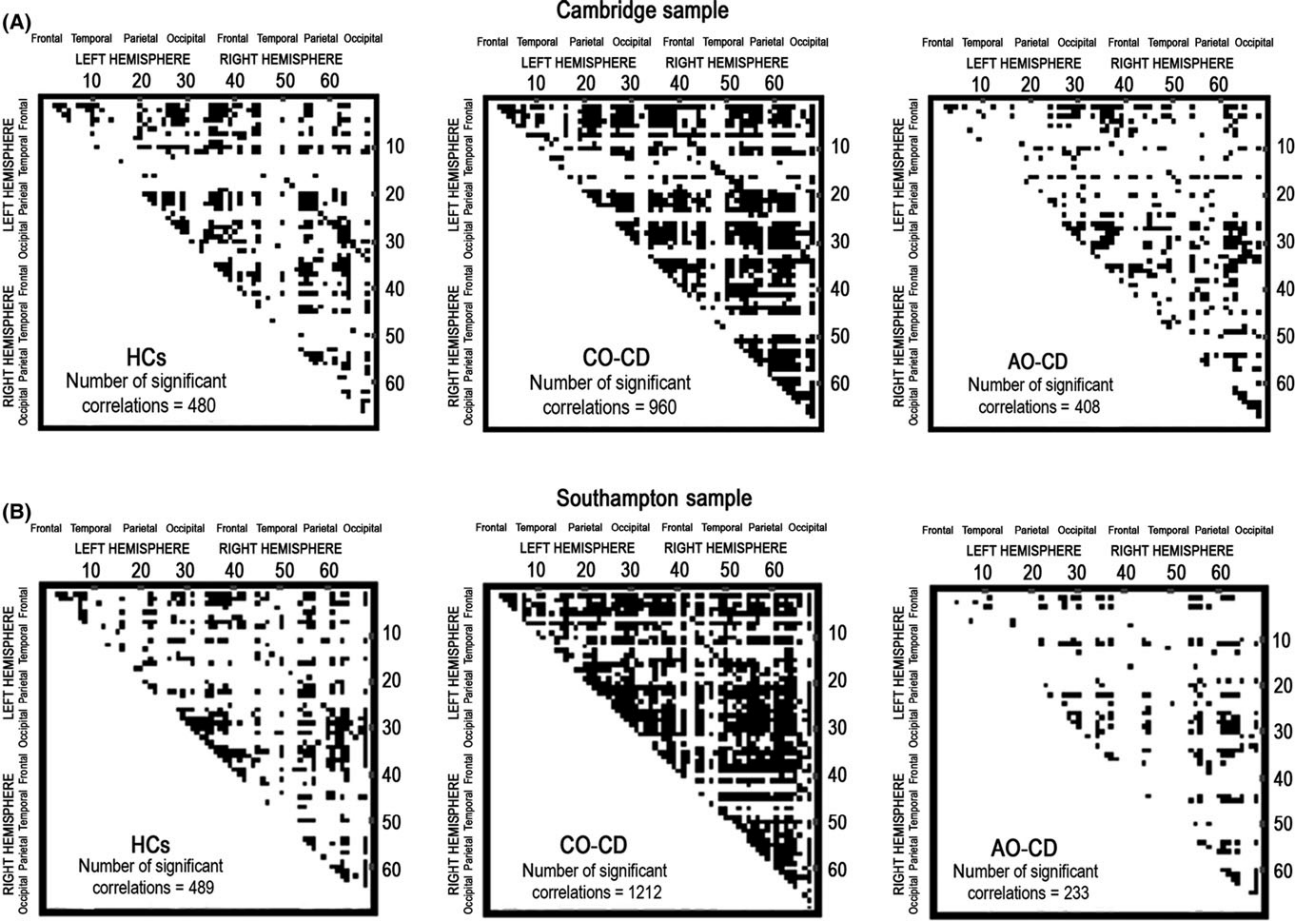
Cross‐cortical correlation matrices between regions in healthy controls (HCs) and youths with childhood‐onset conduct disorder (CO‐CD) and adolescence‐onset conduct disorder (AO‐CD) in the Cambridge (A) and Southampton samples (B). Significant interregional correlations in cortical thickness between pairs of brain regions (when applying a threshold of *p* < .05, false‐discovery‐rate correction for multiple comparisons) are denoted by black dots in the correlation matrices. The *X* and *Y* axes show the 34 regions of interest per hemisphere (i.e. 68 cortical regions in total) from the Desikan–Killiany atlas

In both samples, CO‐CD youths displayed a higher number of significant interregional correlations in cortical thickness than HCs and AO‐CD participants. In contrast, AO‐CD youths showed fewer significant interregional correlations in cortical thickness than HCs (Figure**** 1).

Direct group comparisons revealed significant differences in the strength of the interregional correlations in many regions. In the Cambridge sample, there were 18 significant differences between the HC and CO‐CD groups, 48 differences between the HC and AO‐CD groups, and 118 differences between the CO‐CD and AO‐CD groups. In the Southampton sample, there were 422 significant differences between the HC and CO‐CD groups, 48 differences between the HC and AO‐CD groups, and 68 differences between the CO‐CD and AO‐CD groups.

These differences were localized both within and across frontal, parietal, temporal, and occipital cortices (see Supporting information). The pattern of significant cross‐cortical correlations across the groups and the results of the group comparisons were very similar (albeit stronger) when age, IQ, and ADHD symptoms were not included as covariates (see Supporting information). The general pattern of significant interregional correlations and group differences in correlation strength also remained similar when eTIV was regressed out rather than mean‐centering cortical thickness within each subject prior to computing the correlation coefficients (Cambridge sample: HCs vs. CO‐CD: 21 differences; HCs vs. AO‐CD: 69 differences; CO‐CD vs. AO‐CD: 83 differences; Southampton sample: HCs vs. CO‐CD: 279 differences; HCs vs. AO‐CD: 64 differences; CO‐CD vs. AO‐CD: 106 differences; Supporting information). There were also no significant group differences in the variance of cortical thickness measures or in the variance of eTIV values in the 68 regions included in the analyses in either sample (see Supporting information). Therefore, it is unlikely that the key findings were explained by greater variability in overall cortical thickness or overall brain volume within the CD groups relative to the HCs.

**Figure 2:**
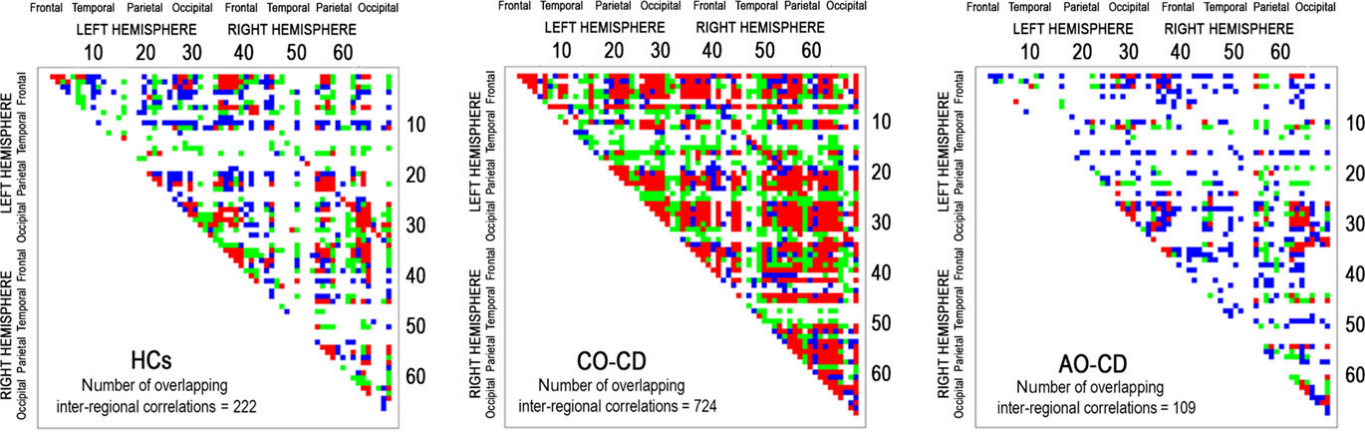
Cross‐cortical correlation matrices showing the degree of overlap (red dots) between the significant interregional correlations identified in the Cambridge and Southampton samples. Blue dots represent correlations that were significant only in the Cambridge sample, whereas green dots represent correlations that were significant only in the Southampton sample. HCs, healthy controls; CO‐CD, childhood‐onset conduct disorder; AO‐CD, adolescence‐onset conduct disorder

Finally, when studying the degree of similarity of the results obtained across the Cambridge and Southampton samples, there was substantial overlap in terms of the significant interregional correlations identified in each sample. In particular, 222 interregional correlations were identified as overlapping between the two HC samples, whereas 724 and 109 overlapping correlations were identified in the two CO‐CD and AO‐CD groups, respectively (Figure**** 2 and Supporting information).

## Discussion

Our first key finding was that youths with CO‐CD showed a strikingly higher number of significant interregional correlations in cortical thickness relative to either HCs or AO‐CD participants, whereas AO‐CD youths displayed fewer significant interregional correlations than HCs and CO‐CD youths. Notably, this pattern held across two independent samples studied in separate locations, although we used different scanners and structural MRI acquisition parameters. Furthermore, there was substantial overlap in terms of the significant interregional correlations identified in each group across two independent samples, which supports the robustness of our findings.

Our second key finding was that there were significant differences between the three groups in the strength of the interregional correlations in cortical thickness, and these were localized both within and across a number of frontal, parietal, temporal, and occipital cortices. Again, there was substantial overlap between the two samples in terms of the correlations that were identified as differing in strength. Overall, these results suggest that alterations in brain structure in CD may be much more widespread than originally thought and may involve several networks, rather than being restricted to specific cortical regions like the ventromedial prefrontal cortex (Blair, 2013). The diffuse pattern of structural covariance abnormalities observed in CD resembles the changes reported in other neurodevelopmental disorders like autism (Alexander‐Bloch et al., 2013), and suggests that, in common with these disorders, CD may be associated with rather global disruptions in brain maturation. Furthermore, group differences in the overall number and strength of the interregional correlations were independent of ADHD comorbidity. This is important as the CD youths differed from HCs in ADHD symptoms, and the CO‐CD participants endorsed more ADHD symptoms than AO‐CD youths, consistent with previous studies (Moffitt & Caspi, 2001). In addition, the results were independent of group differences in IQ, which is noteworthy as Lerch et al. (2006) found that IQ modulates interregional correlations in cortical thickness in typically developing children.

The present findings, which are among the first to demonstrate marked differences in brain structure between the CO and AO subtypes of CD, may be clinically relevant as structural covariance alterations may represent brain‐based biomarkers capable of distinguishing between these forms of CD. Furthermore, demonstrating that youths with CO‐CD differ from their AO‐CD counterparts in the overall number and strength of interregional correlations in cortical thickness supports the notion that age‐of‐onset is an important specifier for CD and should be retained in future classification systems (ICD and DSM).

It is also important to emphasize that our results suggest that both CO‐CD and AO‐CD are associated with changes in the synchronized development of the brain, consistent with the hypothesis that neurobiological factors contribute to the etiology of both CD subtypes (Fairchild et al., 2013) and challenging the view that such factors are not involved in the etiology of AO‐CD (Moffitt, 1993). However, the fact that opposite changes in structural covariance were observed in the CO‐CD and AO‐CD subgroups relative to HCs may seem difficult to reconcile with our earlier work showing relatively few or no differences between the CO‐CD and AO‐CD subtypes in brain structure and function using different neuroimaging modalities and methods (Fairchild et al., 2011; Passamonti et al., 2010). A possible explanation of these apparent inconsistencies is that structural covariance methods are more sensitive in revealing the abnormalities that distinguish between the CO‐CD and AO‐CD subtypes, compared to univariate analyses assessing brain structure or function at the voxel‐wise level.

The biological underpinnings of interregional correlations in cortical thickness are not well understood; hence, we can only speculate regarding the neurodevelopmental basis of the present findings. There is, however, evidence that brain networks identified by applying structural covariance methods to cross‐sectional structural MRI data strongly resemble patterns of maturational coupling between distinct networks (Alexander‐Bloch et al., 2013). This suggests that brain regions that develop according to similar maturational trajectories tend to covary in structure. Accordingly, one interpretation of our findings is that the CO‐CD subgroup shows a more synchronized pattern of brain development than the other groups (as indicated by greater numbers of significant interregional correlations), although this increased synchronization could reflect either accelerated development of cortical regions that normally mature later or delayed development of regions that typically mature earlier in life. Conversely, the reductions in structural covariance observed in AO‐CD youths, relative to HC and CO‐CD participants, may reflect specific disruptions in neurodevelopmental processes that occur during adolescence (e.g. synaptic pruning). In other words, if neuronal pruning is altered in the AO‐CD group against a background of relatively normal brain development in childhood, it is possible that the coordinated pattern of brain development across different cortical regions would be impaired. This might lead to fewer significant interregional correlations in cortical thickness and changes in correlation strength relative to the other groups.

### Strengths and limitations

This is one of the first clinical neuroimaging studies to present findings from independent discovery and replication samples using the same analytic method, despite concerns about the replicability of neuroimaging data (Horga, Kaur, & Peterson, 2014). The degree of correspondence between the two groups of results is particularly encouraging as there were substantial variations between the datasets in terms of MRI acquisition parameters and magnet strengths.

Second, each of the samples was relatively large, homogeneous, and well‐characterized from a psychiatric perspective, with detailed information available from diagnostic interviews and clinical data collected from participants and their parents/carers. In addition, we were able to show that the results did not appear to be explained by group differences in age, IQ, and ADHD symptoms. We note, however, that the CD groups had higher proportions of participants coming from lower socioeconomic strata, and they reported increased rates of substance use relative to HCs. Consequently, future research should examine the contribution of these variables to group differences in structural covariance. Importantly, we also deliberately restricted our sample to males, as there is evidence for sex differences in trajectories of antisocial behavior, as well as in structural covariance measures (Raznahan et al., 2011). Nevertheless, future studies should investigate whether these results generalize to females with CD.

Third, this was the first study to report differences in interregional correlations in cortical thickness between the CO‐CD and AO‐CD subtypes and our results demonstrate the value of this approach. However, we note that retrospective accounts of age‐of‐onset of CD should be treated with caution (Henry, Moffitt, Caspi, Langley, & Silva, 1994), although we attempted to mitigate against this issue by obtaining detailed information from participants and parents and asking both informants to consider salient life landmarks (e.g. the transition from primary to secondary school) to assist accurate recall.

Fourth, we used only a single excitation in the MRI anatomical pulse sequences, which may have reduced signal‐to‐noise ratio and therefore accuracy of the delineation of gray–white matter interfaces. Furthermore, the MACACC method involves performing a large number of statistical tests and it may be considered a data‐driven approach, although we applied a stringent correction for multiple comparisons and the results appeared robust and replicated across two datasets. The MACACC approach is also uninformative about the coordinated development of subcortical regions or covariance between cortical and subcortical regions. Applying structural covariance methods to whole‐brain measurements of gray matter density or white matter microstructure could enable researchers to investigate interactions between cortical and subcortical networks involving the amygdala and striatum, which are critically implicated in CD (Blair, 2013). Nevertheless, the MACACC approach may offer unique information relative to that provided by other methods such as diffusion tensor imaging (DTI). Specifically, there is evidence that MACACC can identify correlations between brain regions that have no direct anatomical connections, as only 35–40% of the interregional correlations revealed using MACACC converged with the connectivity patterns identified via DTI‐based approaches (Gong, He, Chen, & Evans, 2012).

Finally, the present findings suggest several possible avenues for future research. For example, future studies should investigate convergence between structural covariance measures and functional connectivity indices in similar populations. It would also be interesting to integrate cortical thickness‐based methods with DTI‐based approaches (Gong et al., 2012), to examine whether increased structural covariance in CO‐CD is associated with altered white matter microstructural properties. Another interesting question is whether similar changes in structural covariance would be observed in CO‐CD and AO‐CD individuals during later phases of development (e.g. adulthood). Lastly, examining the impact of environmental adversity and substance use on structural covariance measures could help extend the present findings and shed light on psychiatric conditions related to CD (e.g. substance use disorders).

## Conclusions

The present study demonstrates that interregional correlations in cortical thickness may distinguish between the CO and AO forms of CD, and that both CD subgroups significantly differ from typically developing adolescents in both the overall number and strength of the interregional correlations observed. Structural covariance methods may help researchers to refine the diagnosis and classification of CD and improve our ability to distinguish between CD subtypes which may differ in etiology and adult outcomes. Longitudinal studies investigating developmental trajectories of structural covariance, maturational coupling, and functional connectivity in children at high risk for externalizing disorders are now needed to characterize the neurodevelopmental basis of CD and differences between CO‐CD and AO‐CD subtypes in brain maturation.

**Key points**

- Structural covariance analyses are a new set of methods that can be used to investigate the coordinated development of different brain regions.
- We calculated interregional correlations in cortical thickness as a measure of structural covariance in male youths with childhood‐onset conduct disorder (CO‐CD) or adolescence‐onset CD (AO‐CD), and healthy controls (HCs), in two independent samples recruited at different locations.
- In both samples, CO‐CD youths displayed more significant interregional correlations than AO‐CD youths and HCs, whereas AO‐CD individuals displayed fewer correlations than HCs. The three groups also differed in the strength of the interregional correlations across frontal, temporal, parietal, and occipital regions.
- These findings illustrate the value of structural covariance methods in studying psychiatric disorders with putative neurodevelopmental origins like CD.

## Supporting information


**Data S1.** Supplementary methods.
**Data S2.** Supplementary figures.
**Data S3.** Supplementary tables.
