# Supplementary material for: Mapping the structural organization of the brain in conduct disorder: replication of findings in two independent samples
Source: J Child Psychol Psychiatry. 2016 Jun 15;57(9):1018–26. doi: 10.1111/jcpp.12581 (PMC4995723; doi:10.1111/jcpp.12581)
Supplement: Supplementary file 1 — Data S1. Supplementary methods. Data S2. Supplementary figures. Data S3. Supplementary tables. [file JCPP-57-1018-s001.docx]

**Online supplementary material for: *Mapping the structural organization of the brain in Conduct Disorder: Replication of findings in two independent samples, by* Fairchild et al.**

**Appendix S1: supplementary methods**

*Manual Editing of Freesurfer Segmentations*

FreeSurfer segmentations were screened for inaccuracies by a neuroimaging expert blind to group status, and minimal manual edits were performed only when deemed absolutely necessary. Manual correction involved redrawing mask boundaries to exclude non-cortex tissue which had been erroneously included by the surface reconstruction stream. In detail, this involved: 1) excluding small pieces of dura which had been placed within the mask, and 2) correcting erroneous extensions of the pial surface into the cerebellum.

*Non-parametric group-wise comparisons of correlation matrices*

We chose to compare groups using non-parametric, random sampling-based statistics in order to forego/avoid *a priori* assumptions about the distribution of the null hypothesis (i.e., the hypothesis that the between-group difference in correlation strengths between any pair of regions is zero; Good & Phillip, 2006). In detail, when comparing two groups with numerosities n1 and n2, this entails:

a) computing group differences in correlation strength for each pair of regions

b) pooling the n1 + n2 observations/subjects

c) random sampling (with replacement) of two subject groups of sizes n1 and n2 from the pooled data

d) repeating step a) for the groups sampled in c)

e) repeating steps b)-e) N times (in our paper, we chose an extremely large number of samples, i.e. 1,000,000) to build a distribution of group differences in correlation strengths for each pair of regions

f) computing a p-value for each pair of regions based on the position of the metric computed in a) in the distribution computed in e).

g) applying a False Discovery Rate correction (alpha = 0.05) of the matrix of p values across all (68 x 67)/2 = 2278 pairs of regions

*Collection of information about attention-deficit/hyperactivity disorder (ADHD) symptoms*

We administered the full ADHD supplement of the Kiddie-Schedule for Affective Disorders and Schizophrenia-Present and Lifetime Version (K-SADS-PL; Kaufman et al., 1997) to the majority of subjects in both studies, in order to collect dimensional information about ADHD symptoms in the two samples. We have used these data both as covariates of no interest and to perform correlational analyses in our previous studies (e.g., Fairchild et al., 2011, 2013, 2014, 2015; Passamonti et al., 2010). Rather than stopping the assessment of ADHD symptoms if the items in the screening interview were not endorsed, we administered the full ADHD supplement to all participants. This enabled us to comprehensively assess for all 18 of the DSM-IV symptoms of ADHD and evaluate the impact of threshold and sub-threshold ADHD comorbidity on the key findings by regressing out the contribution of ADHD symptoms when assessing for significant inter-regional correlations within each group as well as group differences in the strength of the inter-regional correlations.

**Supplementary Figures S1-S7**

**
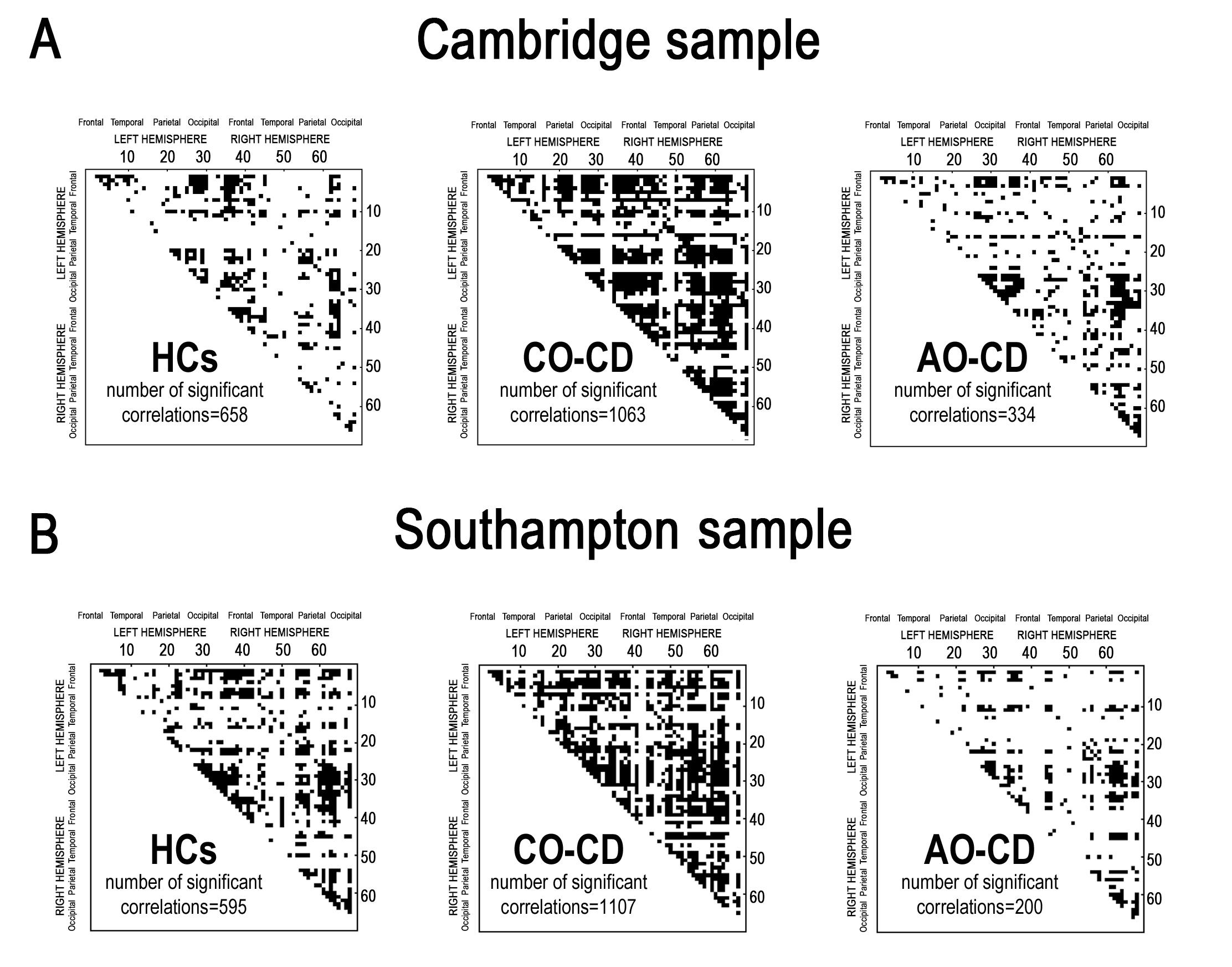
**

**Fig S1.** Cross-cortical correlation matrices between regions in healthy controls (HCs) and youths with childhood-onset conduct disorder (CO-CD), and adolescence-onset conduct disorder (AO-CD) in the Cambridge sample (**A**) and in the Southampton sample (**B**) *when not* including any covariates in the statistical models except for mean-centered cortical thickness. Significant inter-regional correlations in cortical thickness between pairs of brain regions (when applying a threshold of p<0.05, False Discovery Rate correction for multiple comparisons) are denoted by black dots in the correlation matrices. The X and Y axes show the 34 regions of interest per hemisphere from the Desikan-Killiany atlas of cortical regions (see Fig S5). In line with the main analyses that included IQ and ADHD symptoms as covariates of no interest, these analyses showed that youths with CO-CD had a strikingly higher number of significant inter-regional correlations than AO-CD and HC participants, whereas AO-CD youths showed fewer significant inter-regional correlations than HCs.

**
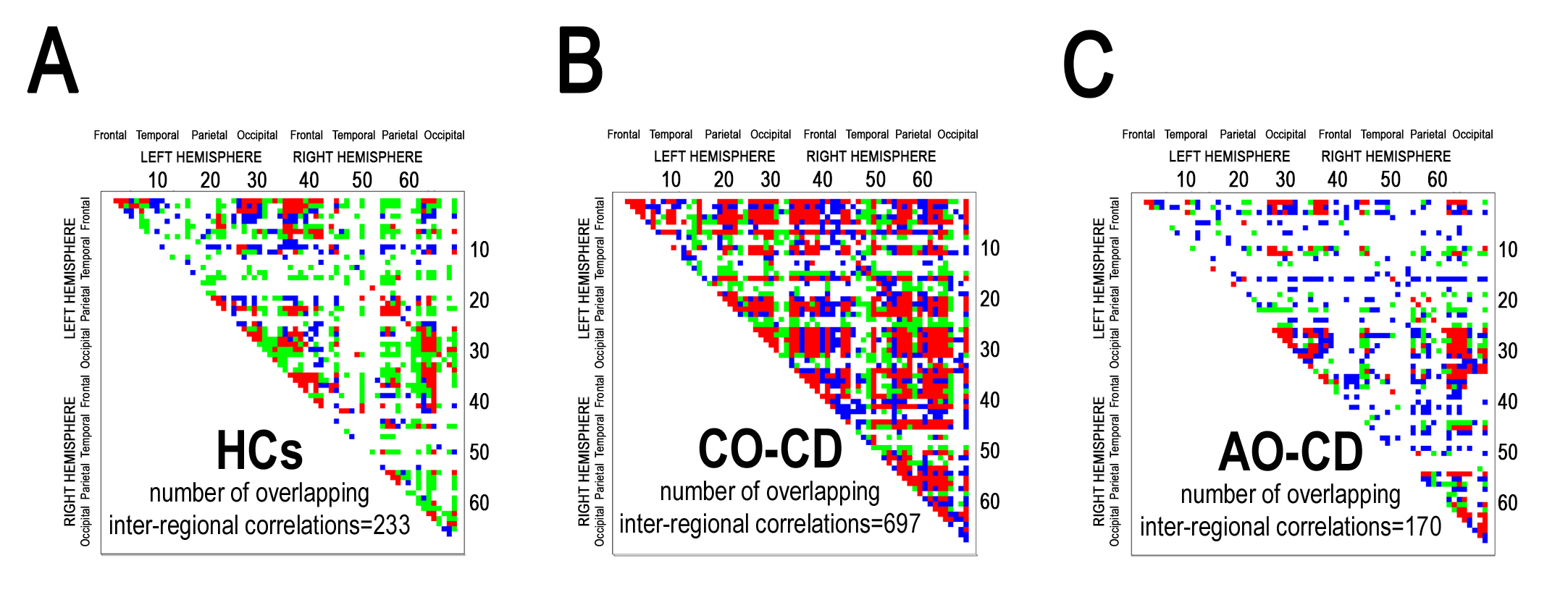
**

**Fig S2.** Cross-cortical correlation matrices showing the degree of overlap (red dots) between the significant inter-regional correlations identified using structural covariance methods in the Cambridge and Southampton samples *when not* including any covariates in the statistical models. Panel A shows the unique and shared cross-cortical correlations observed in healthy controls (HCs), whereas panels B and C show overlap in correlations observed in youths with childhood-onset conduct disorder (CO-CD) and adolescence-onset conduct disorder (AO-CD), respectively. The blue dots represent the correlations that were significant only in the Cambridge sample, whereas the green dots represent correlations that were only significant in the Southampton sample.


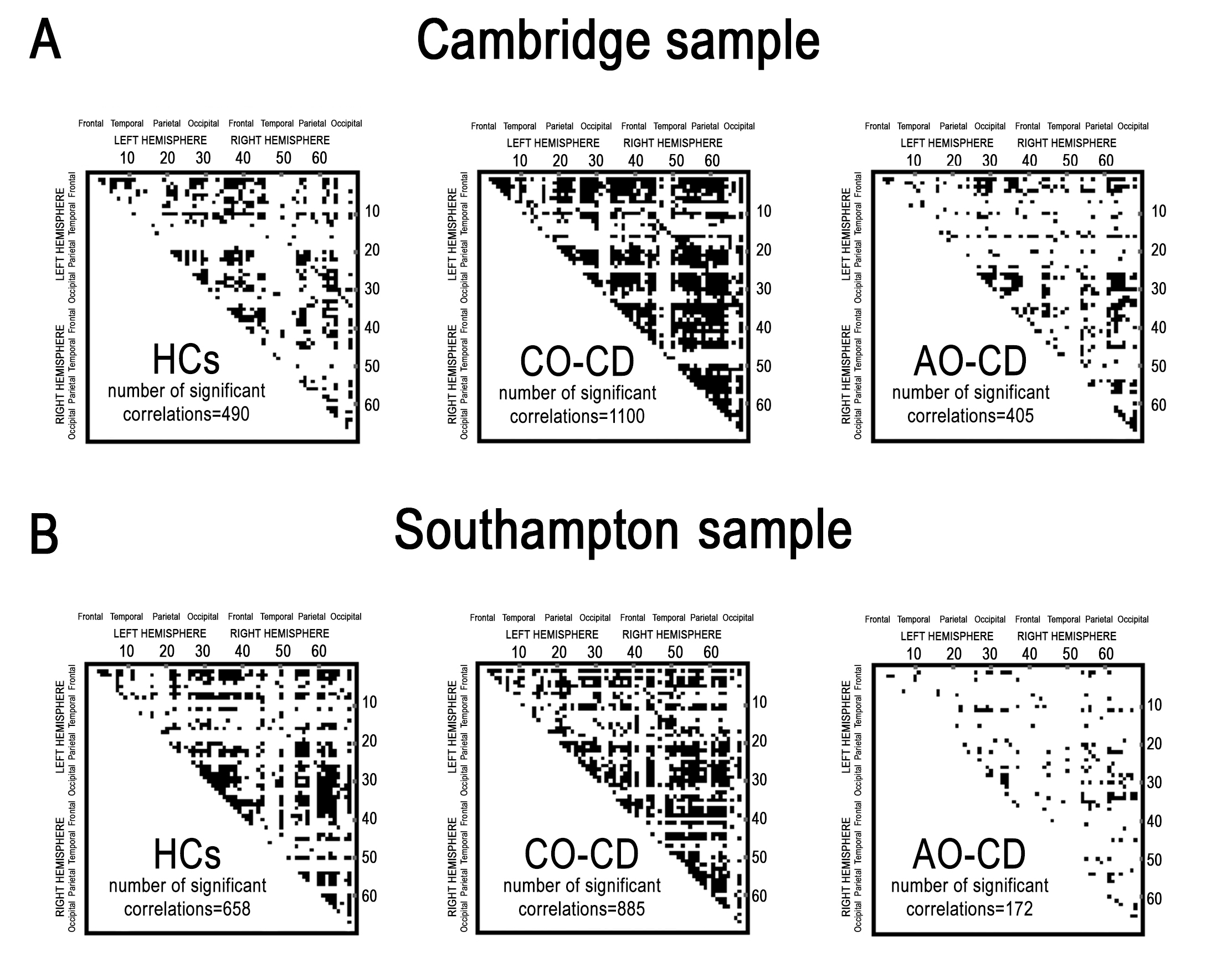


**Fig S3.** Cross-cortical correlation matrices between regions in healthy controls (HCs) and youths with childhood-onset conduct disorder (CO-CD), and adolescence-onset conduct disorder (AO-CD) in the Cambridge sample (**A**) and in the Southampton sample (**B**) *when including* estimated Total Intracranial Volume as a covariate of no interest in the statistical models (as well as the covariates included in the primary analyses, i.e., IQ and ADHD symptoms). Significant inter-regional correlations in cortical thickness between pairs of brain regions (when applying a threshold of p<0.05, False Discovery Rate correction for multiple comparisons) are denoted by black dots in the correlation matrices. The X and Y axes show the 34 regions of interest per hemisphere from the Desikan-Killiany atlas of cortical regions (see Fig S3). In line with the findings reported in the main manuscript (see Fig.1), these analyses showed that youths with CO-CD had a strikingly higher number of significant inter-regional correlations than AO-CD and HC participants, whereas AO-CD youths showed fewer significant correlations than HCs.


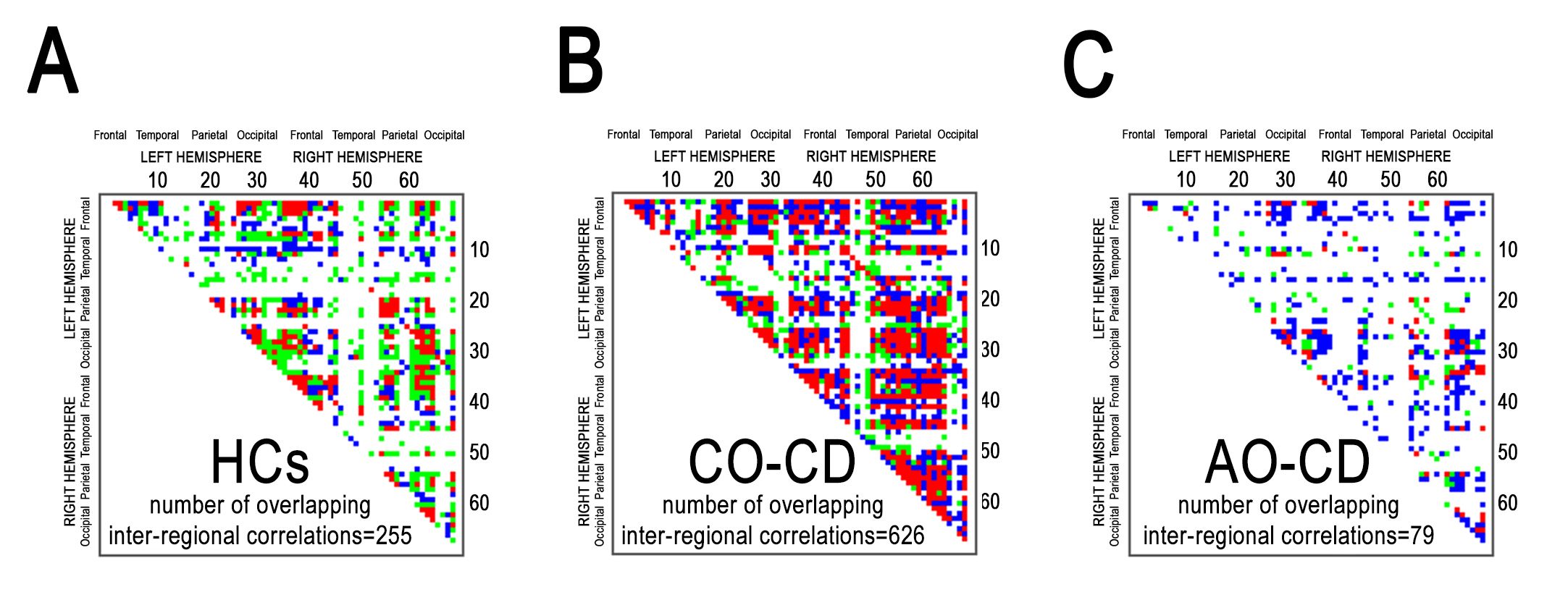


**Fig S4.** Cross-cortical correlation matrices showing the degree of overlap (red dots) between the significant inter-regional correlations identified using structural covariance methods in the Cambridge and Southampton samples *when including* estimated Total Intracranial Volume as covariate of no interest in the statistical models (as well as the covariates included in the primary analyses, i.e., IQ and ADHD symptoms). Panel A shows the unique and shared cross-cortical correlations observed in healthy controls (HCs), whereas panels B and C show overlap in correlations observed in youths with childhood-onset conduct disorder (CO-CD) and adolescence-onset conduct disorder (AO-CD), respectively. The blue dots represent the correlations that were significant only in the Cambridge sample, whereas the green dots represent correlations that were only significant in the Southampton sample.

**Fig S5.** This Figure is based on Figure 1 from *Desikan et al.*, “An automated labeling system for subdividing the human cerebral cortex on MRI scans into gyral based regions of interest”, *Neuroimage,* (2006), 31: 968–980. This extracted picture is intended as a guide for localizing the group differences in the strength of inter-regional correlations between the pairs of cortical regions listed in **Tables S4-S21**. The top panel illustrates the lateral view of the inflated cerebral hemisphere, whereas the bottom panel shows the medial view of the inflated cerebral hemisphere. The yellow asterisks on the lateral inflated surface indicate the cortex around the perimeter of the central sulcus that has been inflated and is therefore visible.


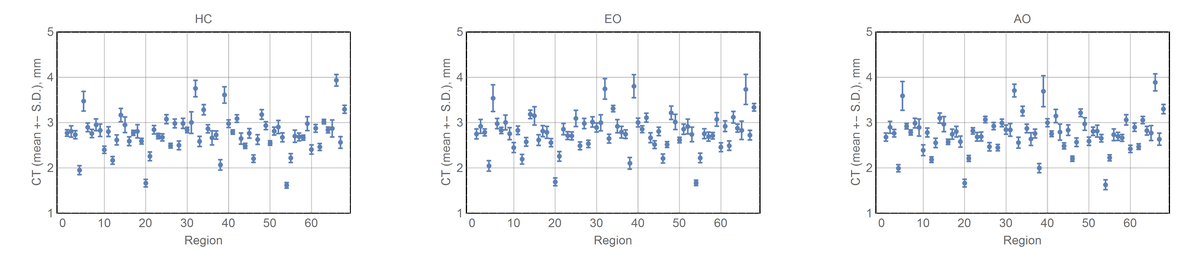


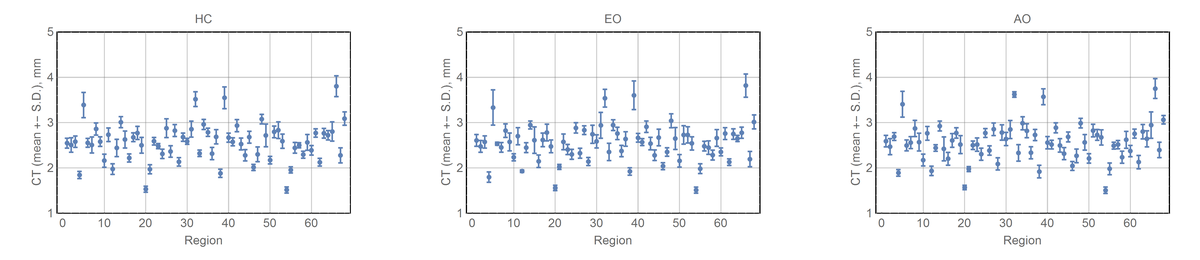


**Fig S6.** Median + Median Absolute Deviation cortical thickness values for each of the 68 cortical regions from the Desikan-Killiany atlas in each group (the values for the Cambridge sample are shown in the top panels, whereas the values for the Southampton sample are shown in the bottom panels). There were no significant group differences in the variance of cortical thickness measures in any of the 68 regions included in the analyses, as assessed using Conover Tests and applying a False Discovery Rate correction for multiple comparisons (p < 0.05). Key: HC, healthy control; EO, childhood-onset Conduct Disorder; AO, adolescence-onset Conduct Disorder. Note: in this figure, uncentered cortical thickness values are shown for visualization purposes, whereas mean-centered values were used to perform the statistical analyses.

**Fig S7.** Median + Median Absolute Deviation estimated Total Intracranial Volume in each group (the values for the Cambridge sample are shown on the left, whereas the values for the Southampton sample are shown on the right). There were no significant group differences in the variance of total intracranial volume, as assessed using Conover Tests. Key: HC, healthy control; EO, childhood-onset Conduct Disorder; AO, adolescence-onset Conduct Disorder.

**Supplementary Tables S1-S21**

**Table S1.** Group comparisons for variance in cortical thickness in each of the 68 cortical regions included in the structural covariance analyses (Conover Test for equal variances). The table shows the P values obtained when applying a False-Discovery Rate correction for multiple comparisons with a threshold of p<0.05 in the Cambridge sample. Key: AO, adolescence-onset Conduct Disorder; EO, childhood-onset Conduct Disorder; HC, healthy control; ROI, region of interest.

|  | Left Hemisphere | |  |  |  | Right Hemisphere | |  |  |
| --- | --- | --- | --- | --- | --- | --- | --- | --- | --- |
| **ROI** | **Main Effect of Group** | **EOvsAO** | **HCvsAO** | **HCvsEO** |  | **Main Effect of Group** | **EOvsAO** | **HCvsAO** | **HCvsEO** |
| bankssts | 0.966 | 0.779 | 0.911 | 0.991 |  | 0.960 | 0.646 | 0.993 | 0.768 |
| caudalanteriorcingulate | 0.854 | 0.673 | 0.799 | 1.000 |  | 0.835 | 0.498 | 0.825 | 0.846 |
| caudalmiddlefrontal | 0.821 | 0.919 | 0.782 | 0.790 |  | 0.988 | 0.828 | 0.838 | 0.848 |
| cuneus | 0.845 | 0.893 | 0.824 | 0.668 |  | 0.983 | 0.872 | 0.964 | 0.971 |
| entorhinal | 0.477 | 0.999 | 0.523 | 0.437 |  | 0.348 | 0.687 | 0.194 | 0.594 |
| frontalpole | 0.935 | 0.724 | 0.888 | 1.000 |  | 0.801 | 0.739 | 0.992 | 0.912 |
| fusiform | 0.569 | 0.340 | 0.920 | 0.815 |  | 0.796 | 0.641 | 1.000 | 0.624 |
| inferiorparietal | 0.855 | 0.748 | 0.860 | 0.859 |  | 0.874 | 0.809 | 0.895 | 0.769 |
| inferiortemporal | 0.872 | 0.672 | 0.992 | 0.781 |  | 0.637 | 0.845 | 0.795 | 0.542 |
| insula | 0.883 | 0.710 | 1.000 | 0.817 |  | 0.821 | 0.618 | 0.704 | 0.846 |
| isthmuscingulate | 0.952 | 0.839 | 0.965 | 1.000 |  | 0.877 | 0.926 | 1.000 | 0.995 |
| lateraloccipital | 0.881 | 0.839 | 0.935 | 0.842 |  | 0.869 | 0.745 | 0.866 | 0.461 |
| lateralorbitofrontal | 0.960 | 0.759 | 1.000 | 0.827 |  | 0.927 | 0.847 | 1.000 | 0.851 |
| lingual | 0.954 | 0.831 | 1.000 | 0.862 |  | 0.815 | 0.741 | 0.916 | 0.960 |
| medialorbitofrontal | 1.000 | 0.703 | 0.718 | 0.896 |  | 0.824 | 0.835 | 0.906 | 0.649 |
| middletemporal | 0.898 | 0.814 | 0.846 | 0.811 |  | 0.885 | 0.679 | 0.964 | 0.659 |
| paracentral | 0.537 | 0.415 | 0.932 | 0.542 |  | 0.858 | 0.809 | 0.813 | 0.579 |
| parahippocampal | 0.994 | 0.900 | 0.914 | 0.995 |  | 0.992 | 0.871 | 0.968 | 0.884 |
| parsopercularis | 0.464 | 0.857 | 0.553 | 0.684 |  | 0.942 | 0.852 | 1.000 | 0.859 |
| parsorbitalis | 0.974 | 0.925 | 0.968 | 0.879 |  | 0.880 | 0.859 | 0.970 | 0.825 |
| parstriangularis | 0.700 | 0.841 | 0.656 | 0.385 |  | 0.845 | 0.829 | 0.952 | 0.647 |
| pericalcarine | 0.919 | 0.840 | 0.835 | 0.794 |  | 0.852 | 0.492 | 0.585 | 0.894 |
| postcentral | 0.538 | 0.445 | 0.958 | 0.665 |  | 0.953 | 0.666 | 0.817 | 0.993 |
| posteriorcingulate | 0.922 | 0.691 | 1.000 | 0.632 |  | 0.872 | 0.655 | 0.984 | 0.870 |
| precentral | 0.830 | 0.701 | 1.000 | 0.622 |  | 0.821 | 0.735 | 0.591 | 0.758 |
| precuneus | 0.832 | 0.418 | 0.924 | 0.855 |  | 0.939 | 0.684 | 0.818 | 0.980 |
| rostralanteriorcingulate | 0.391 | 0.614 | 0.989 | 0.890 |  | 1.000 | 0.804 | 0.959 | 0.922 |
| rostralmiddlefrontal | 1.000 | 0.890 | 0.981 | 0.801 |  | 0.990 | 0.853 | 1.000 | 0.846 |
| superiorfrontal | 0.836 | 0.536 | 0.878 | 0.767 |  | 0.829 | 0.740 | 0.966 | 0.827 |
| superiorparietal | 0.882 | 0.448 | 0.640 | 0.969 |  | 0.578 | 0.445 | 0.749 | 0.739 |
| superiortemporal | 0.911 | 0.558 | 0.820 | 0.855 |  | 0.796 | 0.655 | 0.953 | 0.660 |
| supramarginal | 0.876 | 0.675 | 1.000 | 0.831 |  | 0.837 | 0.811 | 0.806 | 0.577 |
| temporalpole | 0.850 | 0.631 | 0.992 | 0.699 |  | 0.602 | 0.648 | 0.975 | 0.546 |
| transversetemporal | 0.810 | 0.517 | 0.981 | 0.324 |  | 0.963 | 0.694 | 0.869 | 0.985 |

**Table S2.** Group comparisons for variance in cortical thickness in each of the 68 cortical regions included in the structural covariance analyses (performed using the Conover Test for equal variances). The table shows the P values obtained when applying a False-Discovery Rate correction for multiple comparisons with a threshold of p<0.05 in the Southampton sample. Key: AO, adolescence-onset Conduct Disorder; EO, childhood-onset Conduct Disorder; HC, healthy control; ROI, region of interest.

|  | Left Hemisphere | |  |  |  | Right Hemisphere | |  |  |
| --- | --- | --- | --- | --- | --- | --- | --- | --- | --- |
| **ROI** | **Main Effect of Group** | **EOvsAO** | **HCvsAO** | **HCvsEO** |  | **Main Effect of Group** | **EOvsAO** | **HCvsAO** | **HCvsEO** |
| bankssts | 1.000 | 0.799 | 0.947 | 0.799 |  | 0.890 | 0.960 | 0.634 | 0.960 |
| caudalanteriorcingulate | 0.989 | 0.982 | 0.994 | 0.982 |  | 0.997 | 0.939 | 0.998 | 0.939 |
| caudalmiddlefrontal | 1.000 | 1.000 | 0.637 | 1.000 |  | 0.990 | 0.889 | 0.966 | 0.889 |
| cuneus | 0.899 | 0.929 | 0.590 | 0.929 |  | 0.921 | 0.851 | 0.944 | 0.851 |
| entorhinal | 0.696 | 0.785 | 1.000 | 0.785 |  | 0.996 | 1.000 | 0.856 | 1.000 |
| frontalpole | 0.811 | 0.948 | 0.685 | 0.948 |  | 0.752 | 1.000 | 0.987 | 1.000 |
| fusiform | 0.890 | 1.000 | 1.000 | 1.000 |  | 0.959 | 0.786 | 0.659 | 0.786 |
| inferiorparietal | 1.000 | 0.975 | 0.983 | 0.975 |  | 1.000 | 1.000 | 0.580 | 1.000 |
| inferiortemporal | 0.969 | 0.897 | 0.819 | 0.897 |  | 1.000 | 1.000 | 0.972 | 1.000 |
| insula | 1.000 | 0.999 | 0.951 | 0.999 |  | 1.000 | 0.926 | 0.930 | 0.926 |
| isthmuscingulate | 0.572 | 0.947 | 0.064 | 0.947 |  | 0.974 | 0.986 | 0.651 | 0.986 |
| lateraloccipital | 0.484 | 1.000 | 0.897 | 1.000 |  | 0.906 | 1.000 | 0.581 | 1.000 |
| lateralorbitofrontal | 1.000 | 0.904 | 1.000 | 0.904 |  | 1.000 | 1.000 | 0.995 | 1.000 |
| lingual | 0.537 | 0.777 | 0.986 | 0.777 |  | 0.905 | 0.965 | 0.268 | 0.965 |
| medialorbitofrontal | 0.665 | 0.991 | 0.807 | 0.991 |  | 0.539 | 1.000 | 0.995 | 1.000 |
| middletemporal | 0.997 | 1.000 | 0.976 | 1.000 |  | 1.000 | 0.974 | 0.837 | 0.974 |
| paracentral | 0.864 | 0.994 | 0.364 | 0.994 |  | 1.000 | 0.978 | 0.548 | 0.978 |
| parahippocampal | 1.000 | 1.000 | 0.982 | 1.000 |  | 1.000 | 1.000 | 0.642 | 1.000 |
| parsopercularis | 1.000 | 1.000 | 0.982 | 1.000 |  | 0.941 | 0.769 | 0.992 | 0.769 |
| parsorbitalis | 1.000 | 0.881 | 0.658 | 0.881 |  | 0.984 | 0.985 | 0.967 | 0.985 |
| parstriangularis | 0.977 | 1.000 | 0.924 | 1.000 |  | 1.000 | 1.000 | 0.660 | 1.000 |
| pericalcarine | 1.000 | 1.000 | 0.651 | 1.000 |  | 0.988 | 1.000 | 0.975 | 1.000 |
| postcentral | 1.000 | 0.996 | 0.979 | 0.996 |  | 1.000 | 1.000 | 0.615 | 1.000 |
| posteriorcingulate | 0.875 | 1.000 | 0.915 | 1.000 |  | 0.703 | 1.000 | 0.643 | 1.000 |
| precentral | 0.532 | 0.877 | 0.388 | 0.877 |  | 0.218 | 0.974 | 0.363 | 0.974 |
| precuneus | 0.730 | 0.908 | 0.577 | 0.908 |  | 0.584 | 1.000 | 0.056 | 1.000 |
| rostralanteriorcingulate | 0.974 | 1.000 | 0.611 | 1.000 |  | 0.988 | 0.995 | 0.888 | 0.995 |
| rostralmiddlefrontal | 1.000 | 1.000 | 0.722 | 1.000 |  | 0.958 | 0.975 | 0.939 | 0.975 |
| superiorfrontal | 0.957 | 0.973 | 0.867 | 0.973 |  | 1.000 | 1.000 | 1.000 | 1.000 |
| superiorparietal | 0.970 | 1.000 | 0.396 | 1.000 |  | 0.511 | 0.916 | 0.071 | 0.916 |
| superiortemporal | 0.681 | 1.000 | 0.994 | 1.000 |  | 1.000 | 0.919 | 0.839 | 0.919 |
| supramarginal | 0.419 | 1.000 | 0.061 | 1.000 |  | 1.000 | 1.000 | 0.609 | 1.000 |
| temporalpole | 1.000 | 0.993 | 0.837 | 0.993 |  | 0.989 | 0.975 | 0.951 | 0.975 |
| transversetemporal | 0.370 | 0.957 | 0.072 | 0.957 |  | 1.000 | 1.000 | 0.840 | 1.000 |

**Table S3.** Group comparisons for variance in estimated Total Intracranial Volume, performed using the Conover Test for equal variances. Table shows the P values obtained when applying a False-Discovery Rate correction for multiple comparisons with a threshold of p<0.05 in the Cambridge and Southampton samples respectively. **Key:** AO, adolescence-onset Conduct Disorder; CO, childhood-onset Conduct Disorder; HC, healthy control.

|  | | **Main Effect of Group** | | **COvsAO** | **HCvsAO** | | **HCvsCO** | |  |  |
| --- | --- | --- | --- | --- | --- | --- | --- | --- | --- | --- |
| Cambridge | | 0.676251 | | 0.619391 | 0.289486 | | 0.931461 | |  |  |
| Southampton | | 0.605054 | | 0.340187 | 0.417263 | | 0.671728 | |  |  |
| **Table S4.** Group differences in the strength of inter-regional correlations in cortical thickness between youths with childhood-onset conduct disoder (CO-CD) and adolescence-onset conduct disorder (AO-CD) in the Cambridge sample (N=83) after removing variability between groups in age, intelligence quotient, and attention/deficit-hyperactivity disorder symptoms. | | | | | | | | | |  |
|  | |  | | |  | |  | |  |  |
|  | |  | | | **Metric** | | **Metric** | |  |  |
| **Region1** | | **Region2** | | | **CO-CD** | | **AO-CD** | | **Corr P values** |  |
| Right Frontal Pole | | Right CA Cingulate | | | 0.06 | | 0.60 | | 0.00637 | **frontal cortices** |
| Left Orbital Frontal | | Left PreCentral | | | 0.55 | | -0.03 | | 0.00734 | **temporal cortices** |
| Left ParsOpercularis | | Right PreCentral | | | 0.67 | | 0.13 | | 0.00946 | **parietal cortices** |
| Left Orbital Frontal | | Right CA Cingulate | | | 0.51 | | -0.14 | | 0.01641 | **occipital cortices** |
| Left Orbital Frontal | | Left CA Cingulate | | | 0.25 | | -0.34 | | 0.02452 |  |
| Right Caudal Middle Frontal | | Right PreCentral | | | 0.73 | | 0.32 | | 0.02625 |  |
| Left Orbital Frontal | | Right ParsOrbitalis | | | 0.62 | | 0.12 | | 0.02857 |  |
| Left ParsOpercularis | | Right RA cingulate | | | 0.21 | | 0.67 | | 0.02954 |  |
| Left Orbital Frontal | | Right PreCentral | | | 0.65 | | -0.06 | | 0.03089 |  |
| Left PreCentral | | Right Temporal Pole | | | 0.66 | | -0.18 | | 0.00039 |  |
| Left Caudal Middle Frontal | | Right Temporal Pole | | | 0.50 | | -0.32 | | 0.00193 |  |
| Left Orbital Frontal | | Right Temporal Pole | | | 0.77 | | 0.07 | | 0.00212 |  |
| Right ParsOrbitalis | | Right Entorhinal | | | 0.45 | | -0.19 | | 0.00386 |  |
| Left Rostral Middle Frontal | | Right Temporal Pole | | | 0.54 | | -0.21 | | 0.00444 |  |
| Left Superior Frontal | | Right Temporal Pole | | | 0.71 | | 0.07 | | 0.00579 |  |
| Right PreCentral | | Right Temporal Pole | | | 0.61 | | 0.00 | | 0.01100 |  |
| Left Orbital Frontal | | Right Entorhinal | | | 0.52 | | -0.15 | | 0.01216 |  |
| Left Orbital Frontal | | Left Superior Temporal | | | 0.58 | | -0.05 | | 0.01950 |  |
| Right Superior Frontal | | Right Temporal Pole | | | 0.68 | | 0.18 | | 0.02008 |  |
| Right ParsOrbitalis | | Right Parahippocampal | | | 0.53 | | -0.05 | | 0.02973 |  |
| Right Orbital Frontal | | Right Entorhinal | | | 0.57 | | -0.04 | | 0.02992 |  |
| Right ParsOpercularis | | Right Entorhinal | | | 0.38 | | -0.18 | | 0.03552 |  |
| Left ParsOpercularis | | Left Middle Temporal | | | 0.71 | | 0.26 | | 0.03591 |  |
| Left ParsOrbitalis | | Left Superior Temporal | | | 0.46 | | -0.14 | | 0.03919 |  |
| Left Orbital Frontal | | Right Parahippocampal | | | 0.40 | | -0.16 | | 0.03958 |  |
| Right Orbital Frontal | | Right Temporal Pole | | | 0.49 | | -0.05 | | 0.03996 |  |
| Left Caudal Middle Frontal | | Right Isthmus | | | -0.26 | | 0.63 | | 0.00000 |  |
| Right PreCentral | | Right PostCentral | | | 0.71 | | -0.08 | | 0.00077 |  |
| Right Rostral Middle Frontal | | Right Isthmus | | | -0.07 | | 0.68 | | 0.00135 |  |
| Right Superior Frontal | | Right Isthmus | | | 0.03 | | 0.64 | | 0.00290 |  |
| Left Rostral Middle Frontal | | Right Isthmus | | | -0.36 | | 0.34 | | 0.00405 |  |
| Left Superior Frontal | | Right Isthmus | | | 0.08 | | 0.64 | | 0.00946 |  |
| Right CA Cingulate | | Right Isthmus | | | 0.15 | | 0.65 | | 0.01371 |  |
| Left Orbital Frontal | | Right ParaCentral Lobule | | | 0.56 | | -0.05 | | 0.01795 |  |
| Left PreCentral | | Right PostCentral | | | 0.68 | | 0.12 | | 0.02143 |  |
| Left PreCentral | | Right Posterior Cingulate | | | 0.12 | | 0.63 | | 0.02761 |  |
| Left Orbital Frontal | | Right PostCentral | | | 0.50 | | -0.05 | | 0.04749 |  |
| Left Orbital Frontal | | Right Lateral Occipital | | | 0.60 | | 0.04 | | 0.04189 |  |
| Left CA Cingulate | | Right Lingual | | | 0.05 | | -0.43 | | 0.04517 |  |
| Right PreCentral | | Right Insula | | | 0.65 | | 0.01 | | 0.00521 |  |
| Left Orbital Frontal | | Right Insula | | | 0.57 | | 0.07 | | 0.04151 |  |
| Left Parahippocampal | | Right ParsOrbitalis | | | 0.39 | | -0.32 | | 0.00483 |  |
| Left Transverse Temporal | | Right CA Cingulate | | | 0.12 | | 0.56 | | 0.02510 |  |
| Left Middle Temporal | | Right CA Cingulate | | | 0.07 | | 0.61 | | 0.03320 |  |
| Left Middle Temporal | | Right ParsTriangularis | | | 0.68 | | 0.09 | | 0.03475 |  |
| Left Inferior Temporal | | Right ParsTriangularis | | | 0.67 | | 0.14 | | 0.03687 |  |
| Left Banks Superior Temporal | | Right CA Cingulate | | | -0.16 | | 0.46 | | 0.04942 |  |
| Left SupraMarginal | | Right CA Cingulate | | | 0.05 | | 0.63 | | 0.00174 |  |
| Left ParaCentral Lobule | | Right Caudal Middle Frontal | | | 0.66 | | 0.19 | | 0.00676 |  |
| Left Insula | | Right RA cingulate | | | -0.08 | | 0.51 | | 0.03378 |  |
| Left Superior Temporal | | Right Superior Temporal | | | 0.88 | | 0.39 | | 0.00000 |  |
| Left Fusiform | | Right Temporal Pole | | | 0.62 | | -0.22 | | 0.00019 |  |
| Left Superior Temporal | | Right Temporal Pole | | | 0.61 | | -0.22 | | 0.00039 |  |
| Right Temporal Pole | | Right Fusiform | | | 0.57 | | -0.32 | | 0.00039 |  |
| Right Entorhinal | | Right Banks Superior Temporal | | | 0.43 | | -0.21 | | 0.00232 |  |
| Left Fusiform | | Left Inferior Temporal | | | 0.79 | | 0.31 | | 0.00328 |  |
| Left Temporal Pole | | Left Fusiform | | | 0.70 | | 0.09 | | 0.00618 |  |
| Left Superior Temporal | | Right Middle Temporal | | | 0.62 | | 0.01 | | 0.01139 |  |
| Right Parahippocampal | | Right Temporal Pole | | | 0.60 | | -0.03 | | 0.01139 |  |
| Left Inferior Temporal | | Right Entorhinal | | | 0.46 | | -0.12 | | 0.01255 |  |

| Right Temporal Pole | Right Middle Temporal | 0.61 | 0.11 | 0.01467 |
| --- | --- | --- | --- | --- |
| Left Banks Superior Temporal | Right Temporal Pole | 0.12 | -0.49 | 0.01660 |
| Right Temporal Pole | Right Superior Temporal | 0.67 | 0.22 | 0.01699 |
| Left Temporal Pole | Right Superior Temporal | 0.59 | 0.11 | 0.02066 |
| Right Temporal Pole | Right Banks Superior Temporal | 0.42 | -0.18 | 0.02780 |
| Left Superior Temporal | Left Inferior Temporal | 0.63 | 0.15 | 0.02857 |
| Left Middle Temporal | Right Middle Temporal | 0.70 | 0.27 | 0.02896 |
| Left Inferior Temporal | Right Temporal Pole | 0.57 | 0.11 | 0.03070 |
| Right Entorhinal | Right Fusiform | 0.62 | 0.11 | 0.03205 |
| Left Banks Superior Temporal | Right Transverse Temporal | 0.07 | 0.60 | 0.03436 |
| Right Temporal Pole | Right Inferior Temporal | 0.54 | -0.02 | 0.03803 |
| Right Superior Temporal | Right Transverse Temporal | 0.51 | 0.08 | 0.04228 |
| Left Fusiform | Right Entorhinal | 0.51 | 0.09 | 0.04382 |
| Right Entorhinal | Right Parahippocampal | 0.60 | 0.06 | 0.04788 |
| Left Temporal Pole | Right Inferior Temporal | 0.56 | 0.10 | 0.04865 |
| Right Temporal Pole | Right PostCentral | 0.58 | -0.31 | 0.00116 |
| Right Entorhinal | Right PostCentral | 0.49 | -0.26 | 0.00232 |
| Left Transverse Temporal | Right Isthmus | 0.05 | 0.55 | 0.01081 |
| Right Temporal Pole | Right Superior Parietal | 0.56 | -0.02 | 0.01236 |
| Left Temporal Pole | Right PostCentral | 0.41 | -0.22 | 0.02452 |
| Right Transverse Temporal | Right PostCentral | 0.30 | -0.22 | 0.04209 |
| Right Temporal Pole | Right Lateral Occipital | 0.63 | -0.25 | 0.00000 |
| Right Temporal Pole | Right PreCuneus | 0.58 | -0.33 | 0.00019 |
| Left Temporal Pole | Right Cuneus | 0.44 | -0.27 | 0.00598 |
| Right Temporal Pole | Right Lingual | 0.39 | -0.32 | 0.00907 |
| Right Entorhinal | Right Cuneus | 0.52 | -0.06 | 0.01120 |
| Right Entorhinal | Right Lateral Occipital | 0.52 | -0.07 | 0.01737 |
| Left Temporal Pole | Right Lateral Occipital | 0.53 | -0.05 | 0.02297 |
| Left Transverse Temporal | Left Lingual | 0.16 | 0.68 | 0.02819 |
| Left PostCentral | Right Temporal Pole | 0.62 | -0.30 | 0.00019 |
| Right ParaCentral Lobule | Right Temporal Pole | 0.47 | -0.17 | 0.00444 |
| Left Isthmus | Right Entorhinal | -0.30 | 0.40 | 0.00521 |
| Left Isthmus | Left Temporal Pole | -0.09 | 0.48 | 0.01236 |
| Right Isthmus | Right Inferior Temporal | -0.05 | 0.56 | 0.02664 |
| Left SupraMarginal | Right Temporal Pole | 0.54 | 0.01 | 0.02761 |
| Left Superior Parietal | Right Temporal Pole | 0.53 | -0.02 | 0.03127 |
| Left Isthmus | Left Entorhinal | -0.14 | 0.43 | 0.03842 |
| Left Superior Parietal | Right Parahippocampal | 0.40 | -0.03 | 0.04884 |
| Left Lateral Occipital | Right Temporal Pole | 0.69 | -0.16 | 0.00000 |
| Left Cuneus | Right Temporal Pole | 0.33 | -0.50 | 0.00000 |
| Left PreCuneus | Right Temporal Pole | 0.57 | -0.19 | 0.00174 |
| Right Insula | Right Temporal Pole | 0.53 | -0.10 | 0.00347 |
| Left Insula | Right Temporal Pole | 0.50 | -0.24 | 0.00618 |
| Left SupraMarginal | Right Isthmus | 0.08 | 0.79 | 0.00077 |
| Right Isthmus | Right Inferior Parietal | 0.06 | 0.69 | 0.01660 |
| Left ParaCentral Lobule | Right Isthmus | -0.13 | 0.43 | 0.01795 |
| Left PostCentral | Right PostCentral | 0.86 | 0.47 | 0.02664 |
| Right Isthmus | Right Lingual | -0.18 | 0.58 | 0.00097 |
| Right Isthmus | Right Pericalcarine | -0.33 | 0.47 | 0.00097 |
| Left SupraMarginal | Left Lingual | 0.23 | 0.74 | 0.01448 |
| Right Isthmus | Right Cuneus | -0.05 | 0.57 | 0.03610 |
| Left PostCentral | Left Cuneus | 0.19 | 0.73 | 0.03822 |
| Left PostCentral | Left Lingual | 0.44 | 0.82 | 0.04961 |
| Left Lingual | Right Isthmus | -0.25 | 0.52 | 0.00174 |
| Left Pericalcarine | Right Posterior Cingulate | -0.11 | 0.58 | 0.00270 |
| Left Cuneus | Right Posterior Cingulate | -0.14 | 0.45 | 0.02857 |
| Left Lateral Occipital | Right Isthmus | -0.01 | 0.55 | 0.04981 |
| Left Pericalcarine | Right Lingual | 0.10 | 0.64 | 0.01680 |

| **Table S5.** Group differences in the strength of inter-regional correlations in cortical thickness between between healthy controls (HCs) and youths with adolescence-onset conduct disorder (AO-CD) in the Cambridge sample (N=83) after removing variability between groups in age, intelligence quotient, and attention/deficit-hyperactivity disorder symptoms. | | | | |  |
| --- | --- | --- | --- | --- | --- |
|  |  |  |  |  |  |
|  |  | **Metric** | **Metric** |  |  |
| **Region1** | **Region2** | **HCs** | **AO-CD** | **Corr P values** |  |
| Left Rostral Middle Frontal | Right CA Cingulate | -0.28 | 0.42 | 0.00190 | **frontal cortices** |
| Left PreCentral | Right CA Cingulate | -0.04 | 0.61 | 0.00190 | **temporal cortices** |
| Right PreCentral | Right CA Cingulate | -0.25 | 0.45 | 0.00285 | **parietal cortices** |
| Left Caudal Middle Frontal | Right CA Cingulate | -0.22 | 0.48 | 0.00380 | **occipital cortices** |
| Right Frontal Pole | Right CA Cingulate | -0.12 | 0.60 | 0.00712 |  |
| Left Orbital Frontal | Left CA Cingulate | 0.32 | -0.34 | 0.02325 |  |
| Left ParsOpercularis | Right RA cingulate | 0.10 | 0.67 | 0.02373 |  |
| Right Rostral Middle Frontal | Right CA Cingulate | -0.11 | 0.50 | 0.02420 |  |
| Left Orbital Frontal | Left PreCentral | 0.60 | -0.03 | 0.03085 |  |
| Right Superior Frontal | Right CA Cingulate | 0.03 | 0.53 | 0.04414 |  |
| Left PreCentral | Right Superior Temporal | 0.77 | 0.15 | 0.00664 |  |
| Left PreCentral | Right Middle Temporal | 0.62 | 0.01 | 0.01281 |  |
| Right PreCentral | Right Middle Temporal | 0.62 | 0.08 | 0.02231 |  |
| Right RA cingulate | Right Banks Superior Temporal | -0.25 | 0.38 | 0.03417 |  |
| Left CA Cingulate | Right Inferior Parietal | 0.40 | -0.20 | 0.00142 |  |
| Left RA cingulate | Right Isthmus | -0.44 | 0.35 | 0.00759 |  |
| Left Caudal Middle Frontal | Right Isthmus | -0.05 | 0.63 | 0.02231 |  |
| Right Superior Frontal | Right Isthmus | -0.10 | 0.64 | 0.02753 |  |
| Right Rostral Middle Frontal | Right Isthmus | 0.01 | 0.68 | 0.03322 |  |
| Right CA Cingulate | Right Isthmus | 0.06 | 0.65 | 0.03370 |  |
| Right RA cingulate | Right Isthmus | -0.33 | 0.44 | 0.03939 |  |
| Left Superior Frontal | Right Isthmus | -0.04 | 0.64 | 0.04888 |  |
| Left CA Cingulate | Left Insula | 0.67 | 0.02 | 0.00570 |  |
| Left ParsOrbitalis | Right Lingual | -0.24 | 0.40 | 0.02705 |  |
| Left Caudal Middle Frontal | Right Lingual | -0.02 | 0.64 | 0.02895 |  |
| Left Transverse Temporal | Right CA Cingulate | -0.24 | 0.56 | 0.00000 |  |
| Left Middle Temporal | Right ParsTriangularis | 0.74 | 0.09 | 0.01092 |  |
| Right ParaCentral Lobule | Right CA Cingulate | -0.13 | 0.54 | 0.00807 |  |
| Left SupraMarginal | Right CA Cingulate | 0.05 | 0.63 | 0.01946 |  |
| Left Isthmus | Right ParsOrbitalis | -0.28 | 0.36 | 0.02515 |  |
| Right Temporal Pole | Right Fusiform | 0.49 | -0.32 | 0.00380 |  |
| Left Middle Temporal | Right Middle Temporal | 0.76 | 0.27 | 0.01471 |  |
| Left Fusiform | Left Superior Temporal | 0.63 | 0.14 | 0.02990 |  |
| Left Superior Temporal | Left Inferior Temporal | 0.66 | 0.15 | 0.04793 |  |
| Left Transverse Temporal | Right Isthmus | -0.26 | 0.55 | 0.00000 |  |
| Left Banks Superior Temporal | Left Inferior Parietal | 0.64 | 0.09 | 0.03797 |  |
| Right Temporal Pole | Right PostCentral | 0.54 | -0.31 | 0.04414 |  |
| Right Temporal Pole | Right PreCuneus | 0.43 | -0.33 | 0.02231 |  |
| Left Entorhinal | Left Pericalcarine | 0.31 | -0.36 | 0.04366 |  |
| Left Isthmus | Left Entorhinal | -0.40 | 0.43 | 0.01186 |  |
| Right ParaCentral Lobule | Right Middle Temporal | 0.67 | 0.11 | 0.03892 |  |
| Left SupraMarginal | Right Isthmus | -0.19 | 0.79 | 0.00095 |  |
| Right Isthmus | Right SupraMarginal | -0.29 | 0.52 | 0.00427 |  |
| Left ParaCentral Lobule | Left Pericalcarine | 0.52 | -0.08 | 0.00807 |  |
| Left ParaCentral Lobule | Right Lateral Occipital | 0.70 | 0.31 | 0.02325 |  |
| Left ParaCentral Lobule | Left Cuneus | 0.60 | 0.11 | 0.02515 |  |
| Left Insula | Left PostCentral | -0.08 | 0.62 | 0.03417 |  |
| Left Insula | Left Lingual | -0.05 | 0.69 | 0.03322 |  |

**Table S6.** Group differences in the strength of inter-regional correlations in cortical thickness between healthy controls (HCs) and youths with childhood-onset conduct disorder (CO-CD) in the Cambridge sample (N=83) after removing variability between groups in age, intelligence quotient, and attention/deficit-hyperactivity disorder symptoms.

|  |  | **Metric** | **Metric** |  |  |
| --- | --- | --- | --- | --- | --- |
| **Region1** | **Region2** | **HCs** | **CO-CD** | **Corr P values** |  |
| Right PreCentral | Right CA Cingulate | -0.25 | 0.41 | 0.00759 | **frontal cortices** |
| Right ParsTriangularis | Right Entorhinal | -0.06 | 0.62 | 0.00000 | **temporal cortices** |
| Left PreCentral | Right Transverse Temporal | 0.67 | 0.08 | 0.02911 | **parietal cortices** |
| Left Superior Frontal | Right Temporal Pole | 0.24 | 0.71 | 0.04429 | **occipital cortices** |
| Left Orbital Frontal | Left Pericalcarine | 0.59 | -0.03 | 0.02278 |  |
| Left CA Cingulate | Left Insula | 0.67 | 0.05 | 0.00506 |  |
| Left Inferior Temporal | Right ParsOrbitalis | -0.23 | 0.66 | 0.00000 |  |
| Left Entorhinal | Right Caudal Middle Frontal | -0.15 | 0.43 | 0.03164 |  |
| Left Temporal Pole | Left Inferior Temporal | 0.01 | 0.60 | 0.01139 |  |
| Right Entorhinal | Right Inferior Temporal | -0.03 | 0.59 | 0.01266 |  |
| Left Parahippocampal | Right Temporal Pole | -0.08 | 0.45 | 0.04303 |  |
| Right Banks Superior Temporal | Right SupraMarginal | 0.04 | 0.66 | 0.00000 |  |
| Right Parahippocampal | Right PostCentral | -0.28 | 0.49 | 0.00127 |  |
| Left Superior Parietal | Right Entorhinal | -0.09 | 0.56 | 0.00506 |  |
| Left Pericalcarine | Right Transverse Temporal | 0.57 | -0.14 | 0.00127 |  |
| Left Lateral Occipital | Right Entorhinal | -0.16 | 0.51 | 0.01139 |  |
| Left PreCuneus | Right Temporal Pole | 0.07 | 0.57 | 0.03164 |  |
| Left Lingual | Right Isthmus | 0.52 | -0.25 | 0.00633 |  |

**Table S7.** Group differences in the strength of inter-regional correlations in cortical thickness between youths with childhood-onset conduct disorder (CO-CD) and adolescence-onset conduct disorder (AO-CD) in the Southampton sample (N=69) after removing variability between groups in intelligence quotient and attention/deficit-hyperactivity disorder symptoms.

|  |  | **Metric** | **Metric** |  |  |
| --- | --- | --- | --- | --- | --- |
| **Region1** | **Region2** | **CO-CD** | **AO-CD** | **Corr P values** |  |
| Left Frontal Pole | Right CA Cingulate | 0.51 | -0.55 | 0.00402 | **frontal cortices** |
| Right ParsOpercularis | Right Orbital Frontal | 0.86 | 0.03 | 0.03585 | **temporal cortices** |
| Right ParsOpercularis | Right Inferior Temporal | 0.73 | -0.10 | 0.00436 | **parietal cortices** |
| Left ParsOpercularis | Right Entorhinal | 0.46 | -0.37 | 0.02580 | **occipital cortices** |
| Left ParsTriangularis | Right Temporal Pole | 0.61 | -0.19 | 0.03886 |  |
| Left PreCentral | Left Temporal Pole | 0.31 | -0.59 | 0.04657 |  |
| Left Temporal Pole | Right PreCentral | 0.38 | -0.69 | 0.00871 |  |
| Left Parahippocampal | Right Superior Frontal | 0.58 | -0.25 | 0.01608 |  |
| Left Parahippocampal | Right PreCentral | 0.57 | -0.22 | 0.02948 |  |
| Left Parahippocampal | Right Caudal Middle Frontal | 0.59 | -0.17 | 0.03618 |  |
| Left Lingual | Right CA Cingulate | 0.62 | -0.41 | 0.01106 |  |
| Left Pericalcarine | Right RA cingulate | 0.25 | -0.52 | 0.02345 |  |
| Right Temporal Pole | Right Inferior Temporal | 0.74 | -0.03 | 0.00670 |  |
| Left Temporal Pole | Right Middle Temporal | 0.58 | -0.72 | 0.01139 |  |
| Left Parahippocampal | Left Middle Temporal | 0.60 | -0.22 | 0.01307 |  |
| Left Temporal Pole | Left Middle Temporal | 0.63 | -0.59 | 0.02379 |  |
| Left Entorhinal | Right Middle Temporal | 0.55 | -0.42 | 0.02412 |  |
| Left Superior Temporal | Left Banks Superior Temporal | 0.65 | -0.25 | 0.02881 |  |
| Left Entorhinal | Right Fusiform | 0.50 | -0.28 | 0.04322 |  |
| Left Entorhinal | Left Middle Temporal | 0.64 | -0.29 | 0.04389 |  |
| Left Temporal Pole | Right Superior Parietal | 0.47 | -0.72 | 0.00134 |  |
| Left Temporal Pole | Right Inferior Parietal | 0.54 | -0.59 | 0.00335 |  |
| Left Temporal Pole | Left Inferior Parietal | 0.57 | -0.55 | 0.00637 |  |
| Left Parahippocampal | Right PostCentral | 0.39 | -0.50 | 0.01106 |  |
| Left Entorhinal | Right Inferior Parietal | 0.65 | -0.32 | 0.01240 |  |
| Left Temporal Pole | Left Superior Parietal | 0.56 | -0.61 | 0.01742 |  |
| Left Entorhinal | Left Superior Parietal | 0.44 | -0.54 | 0.02245 |  |
| Left Parahippocampal | Right SupraMarginal | 0.57 | -0.35 | 0.02446 |  |
| Left Temporal Pole | Right SupraMarginal | 0.35 | -0.59 | 0.02580 |  |
| Left Entorhinal | Right Superior Parietal | 0.33 | -0.53 | 0.02781 |  |
| Left Parahippocampal | Left PostCentral | 0.43 | -0.39 | 0.02881 |  |
| Right Temporal Pole | Right Inferior Parietal | 0.60 | -0.28 | 0.02948 |  |
| Left Entorhinal | Right ParaCentral Lobule | 0.41 | -0.47 | 0.04188 |  |

| Left Entorhinal | Left Inferior Parietal | 0.54 | -0.41 | 0.04255 |  |
| --- | --- | --- | --- | --- | --- |
| Right Temporal Pole | Right Superior Parietal | 0.63 | -0.34 | 0.04322 |  |
| Left Superior Temporal | Right Inferior Parietal | 0.80 | 0.23 | 0.04724 |  |
| Right Temporal Pole | Right Pericalcarine | 0.60 | -0.49 | 0.00000 |  |
| Left Temporal Pole | Right Lingual | 0.48 | -0.73 | 0.00067 |  |
| Left Entorhinal | Right Lateral Occipital | 0.44 | -0.59 | 0.00201 |  |
| Left Entorhinal | Left Lateral Occipital | 0.40 | -0.54 | 0.00235 |  |
| Left Temporal Pole | Left Lingual | 0.41 | -0.63 | 0.00235 |  |
| Left Temporal Pole | Right Lateral Occipital | 0.41 | -0.80 | 0.00235 |  |
| Left Temporal Pole | Left Lateral Occipital | 0.34 | -0.76 | 0.00469 |  |
| Left Temporal Pole | Left Cuneus | 0.39 | -0.61 | 0.00771 |  |
| Left Entorhinal | Right Lingual | 0.48 | -0.48 | 0.00838 |  |
| Right Temporal Pole | Right Lingual | 0.42 | -0.53 | 0.00838 |  |
| Left Entorhinal | Left Lingual | 0.28 | -0.59 | 0.00871 |  |
| Left Temporal Pole | Right PreCuneus | 0.55 | -0.50 | 0.00938 |  |
| Right Temporal Pole | Right Cuneus | 0.21 | -0.68 | 0.01173 |  |
| Left Temporal Pole | Left PreCuneus | 0.62 | -0.38 | 0.02077 |  |
| Left Superior Temporal | Left Lingual | 0.80 | 0.09 | 0.02312 |  |
| Left Entorhinal | Right PreCuneus | 0.59 | -0.32 | 0.03015 |  |
| Left Entorhinal | Right Pericalcarine | 0.38 | -0.44 | 0.03551 |  |
| Right Inferior Temporal | Right Pericalcarine | 0.51 | -0.13 | 0.03853 |  |
| Right Temporal Pole | Right PreCuneus | 0.60 | -0.22 | 0.03886 |  |
| Right Isthmus | Right Temporal Pole | 0.58 | -0.33 | 0.00268 |  |
| Right Isthmus | Right Parahippocampal | 0.57 | -0.39 | 0.01776 |  |
| Left ParaCentral Lobule | Right Temporal Pole | 0.55 | -0.35 | 0.02714 |  |
| Right ParaCentral Lobule | Right Temporal Pole | 0.53 | -0.29 | 0.04456 |  |
| Left Pericalcarine | Right Temporal Pole | 0.63 | -0.42 | 0.00034 |  |
| Left Lingual | Right Temporal Pole | 0.62 | -0.52 | 0.00067 |  |
| Left Cuneus | Right Temporal Pole | 0.54 | -0.52 | 0.00201 |  |
| Left Lateral Occipital | Right Temporal Pole | 0.43 | -0.41 | 0.04657 |  |
| Right ParaCentral Lobule | Right Superior Parietal | 0.51 | 0.93 | 0.02680 |  |
| Left ParaCentral Lobule | Left Lateral Occipital | 0.41 | 0.88 | 0.01340 |  |
| Right ParaCentral Lobule | Right Lateral Occipital | 0.47 | 0.92 | 0.01776 |  |
| Left Lateral Occipital | Right ParaCentral Lobule | 0.56 | 0.93 | 0.04858 |  |
| Left Cuneus | Right Cuneus | 0.51 | 0.86 | 0.02278 |  |
| **Table S8.** Group differences in the strength of inter-regional correlations in cortical thickness between healthy controls (HCs) and youths with adolescence-onset conduct disorder (AO-CD) in the Southampton sample (N=69) after removing variability between groups in intelligence quotient and attention/deficit-hyperactivity disorder symptoms. | | | | |  |
|  |  |  |  |  |  |
|  |  | **Metric** | **Metric** |  |  |
| **Region1** | **Region2** | **HCs** | **AO-CD** | **Corr P values** |  |
| Left PreCentral | Right Caudal Middle Frontal | 0.11 | 0.88 | 0.00000 | **frontal cortices** |
| Left Caudal Middle Frontal | Left PreCentral | 0.17 | 0.82 | 0.00285 | **temporal cortices** |
| Left PreCentral | Right PreCentral | 0.32 | 0.86 | 0.00522 | **parietal cortices** |
| Right Frontal Pole | Right RA cingulate | -0.18 | 0.67 | 0.00902 | **occipital cortices** |
| Left Frontal Pole | Right CA Cingulate | 0.34 | -0.55 | 0.01946 |  |
| Left PreCentral | Right ParsTriangularis | -0.06 | 0.68 | 0.02088 |  |
| Left ParsOpercularis | Left RA cingulate | -0.21 | 0.52 | 0.03654 |  |
| Left PreCentral | Right Middle Temporal | -0.03 | 0.77 | 0.00142 |  |
| Left Caudal Middle Frontal | Right Transverse Temporal | -0.09 | 0.69 | 0.03464 |  |
| Right PreCentral | Right Middle Temporal | 0.32 | 0.82 | 0.04698 |  |
| Left PreCentral | Left Inferior Parietal | -0.04 | 0.74 | 0.01186 |  |
| Left Superior Frontal | Right SupraMarginal | 0.44 | 0.90 | 0.03322 |  |
| Left PreCentral | Right Superior Parietal | -0.11 | 0.63 | 0.03559 |  |
| Left CA Cingulate | Left PreCuneus | 0.09 | 0.75 | 0.02515 |  |
| Left Frontal Pole | Left Pericalcarine | 0.36 | -0.35 | 0.04129 |  |
| Left Transverse Temporal | Right ParsTriangularis | -0.34 | 0.54 | 0.00854 |  |
| Left Temporal Pole | Right Middle Temporal | 0.48 | -0.72 | 0.00000 |  |
| Left Middle Temporal | Left Transverse Temporal | 0.00 | 0.73 | 0.00617 |  |
| Left Transverse Temporal | Right Transverse Temporal | 0.31 | 0.85 | 0.01139 |  |
| Left Fusiform | Right Inferior Temporal | 0.35 | 0.88 | 0.04414 |  |
| Left Temporal Pole | Right Fusiform | 0.33 | -0.46 | 0.04651 |  |
| Left Temporal Pole | Left Middle Temporal | 0.18 | -0.59 | 0.04983 |  |
| Right Middle Temporal | Right Superior Parietal | -0.03 | 0.86 | 0.00095 |  |

| Left Temporal Pole | Right ParaCentral Lobule | 0.32 | -0.69 | 0.00617 |  |
| --- | --- | --- | --- | --- | --- |
| Left Middle Temporal | Right Superior Parietal | 0.19 | 0.84 | 0.00617 |  |
| Right Middle Temporal | Right SupraMarginal | 0.37 | 0.87 | 0.00997 |  |
| Left Parahippocampal | Right PostCentral | 0.33 | -0.50 | 0.01376 |  |
| Left Temporal Pole | Right Inferior Parietal | 0.24 | -0.59 | 0.01614 |  |
| Left Banks Superior Temporal | Right ParaCentral Lobule | 0.02 | 0.69 | 0.01709 |  |
| Left Temporal Pole | Right Superior Parietal | -0.04 | -0.72 | 0.04081 |  |
| Left Temporal Pole | Left Lateral Occipital | 0.15 | -0.76 | 0.00237 |  |
| Left Temporal Pole | Left Lingual | 0.26 | -0.63 | 0.00332 |  |
| Left Temporal Pole | Right Lateral Occipital | 0.10 | -0.80 | 0.00380 |  |
| Left Entorhinal | Left Lingual | 0.25 | -0.59 | 0.00997 |  |
| Left Temporal Pole | Right Lingual | -0.03 | -0.73 | 0.01993 |  |
| Left Transverse Temporal | Right Insula | -0.10 | 0.73 | 0.00047 |  |
| Left ParaCentral Lobule | Right Middle Temporal | 0.31 | 0.89 | 0.00332 |  |
| Right Posterior Cingulate | Right Transverse Temporal | -0.36 | 0.54 | 0.02278 |  |
| Left PreCuneus | Right Middle Temporal | 0.15 | 0.79 | 0.03607 |  |
| Left Insula | Left Transverse Temporal | -0.30 | 0.53 | 0.01044 |  |
| Right ParaCentral Lobule | Right Superior Parietal | 0.40 | 0.93 | 0.00095 |  |
| Left ParaCentral Lobule | Right Superior Parietal | 0.35 | 0.92 | 0.00142 |  |
| Right Superior Parietal | Right PreCuneus | 0.54 | 0.94 | 0.00000 |  |
| Left ParaCentral Lobule | Left PreCuneus | 0.40 | 0.94 | 0.00617 |  |
| Left ParaCentral Lobule | Right PreCuneus | 0.40 | 0.90 | 0.01234 |  |
| Right ParaCentral Lobule | Right PreCuneus | 0.44 | 0.89 | 0.01234 |  |
| Left Superior Parietal | Right PreCuneus | 0.50 | 0.90 | 0.04651 |  |
| Left Cuneus | Right Cuneus | 0.42 | 0.86 | 0.00949 |  |
| **Table S9.** Group differences in the strength of inter-regional correlations in cortical thickness between healthy controls (HCs) and youths with childhood-onset conduct disorder (CO-CD) in the Southampton sample (N=69) after removing variability between groups in intelligence quotient and attention/deficit-hyperactivity disorder symptoms. | | | | |  |
|  |  |  |  |  |  |
|  |  | **Metric** | **Metric** |  |  |
| **Region1** | **Region2** | **HCs** | **CO-CD** | **Corr P values** |  |
| Left PreCentral | Right PreCentral | 0.32 | 0.86 | 0.00000 | **frontal cortices** |
| Left ParsOpercularis | Left ParsTriangularis | 0.35 | 0.79 | 0.00027 | **temporal cortices** |
| Left PreCentral | Right Caudal Middle Frontal | 0.11 | 0.68 | 0.00032 | **parietal cortices** |
| Left PreCentral | Right ParsTriangularis | -0.06 | 0.62 | 0.00032 | **occipital cortices** |
| Right ParsOpercularis | Right Orbital Frontal | 0.40 | 0.86 | 0.00049 |  |
| Right Medial Orbital Frontal | Right RA cingulate | 0.71 | 0.01 | 0.00076 |  |
| Left PreCentral | Right Superior Frontal | 0.07 | 0.76 | 0.00086 |  |
| Left Superior Frontal | Right PreCentral | 0.28 | 0.81 | 0.00092 |  |
| Left PreCentral | Left CA Cingulate | -0.35 | 0.42 | 0.00119 |  |
| Right ParsTriangularis | Right PreCentral | 0.28 | 0.77 | 0.00119 |  |
| Left ParsTriangularis | Right PreCentral | 0.35 | 0.77 | 0.00157 |  |
| Left Caudal Middle Frontal | Left PreCentral | 0.17 | 0.67 | 0.00297 |  |
| Left Caudal Middle Frontal | Right PreCentral | 0.38 | 0.77 | 0.00432 |  |
| Left Superior Frontal | Left PreCentral | 0.16 | 0.70 | 0.00475 |  |
| Right Superior Frontal | Right PreCentral | 0.37 | 0.78 | 0.00810 |  |
| Right Caudal Middle Frontal | Right PreCentral | 0.57 | 0.83 | 0.01085 |  |
| Left Orbital Frontal | Left PreCentral | 0.13 | 0.66 | 0.01387 |  |
| Left CA Cingulate | Right PreCentral | -0.15 | 0.40 | 0.01414 |  |
| Left PreCentral | Right Orbital Frontal | -0.04 | 0.49 | 0.01490 |  |
| Left ParsTriangularis | Left PreCentral | 0.24 | 0.66 | 0.01695 |  |
| Left ParsOpercularis | Right PreCentral | 0.34 | 0.72 | 0.01911 |  |
| Left ParsTriangularis | Right Superior Frontal | 0.35 | 0.69 | 0.02159 |  |
| Left Superior Frontal | Left ParsTriangularis | 0.38 | 0.72 | 0.02262 |  |
| Left ParsOpercularis | Left CA Cingulate | -0.24 | 0.44 | 0.02413 |  |
| Left Rostral Middle Frontal | Right Medial Orbital Frontal | 0.72 | 0.27 | 0.02489 |  |
| Left Medial Orbital Frontal | Left PreCentral | -0.04 | 0.41 | 0.02737 |  |
| Left CA Cingulate | Right Frontal Pole | 0.39 | -0.15 | 0.02758 |  |
| Left Caudal Middle Frontal | Left ParsOpercularis | 0.28 | 0.67 | 0.02818 |  |
| Left ParsTriangularis | Right ParsOpercularis | 0.29 | 0.69 | 0.02915 |  |
| Right ParsOrbitalis | Right RA cingulate | 0.36 | -0.11 | 0.03336 |  |
| Left ParsTriangularis | Right Caudal Middle Frontal | 0.33 | 0.65 | 0.03876 |  |
| Left Superior Frontal | Right ParsOpercularis | 0.54 | 0.81 | 0.04648 |  |
| Left Frontal Pole | Left CA Cingulate | -0.07 | 0.50 | 0.04928 |  |
| Left Superior Frontal | Right Temporal Pole | -0.06 | 0.67 | 0.00000 |  |

| Left ParsOpercularis | Right Parahippocampal | -0.16 | 0.66 | 0.00000 |
| --- | --- | --- | --- | --- |
| Right ParsOpercularis | Right Temporal Pole | -0.06 | 0.63 | 0.00000 |
| Left Caudal Middle Frontal | Right Transverse Temporal | -0.09 | 0.68 | 0.00005 |
| Left ParsTriangularis | Left Transverse Temporal | -0.25 | 0.67 | 0.00005 |
| Left PreCentral | Left Parahippocampal | -0.14 | 0.57 | 0.00005 |
| Right PreCentral | Right Temporal Pole | -0.21 | 0.58 | 0.00005 |
| Left Caudal Middle Frontal | Right Parahippocampal | -0.26 | 0.58 | 0.00011 |
| Left ParsTriangularis | Right Parahippocampal | -0.15 | 0.74 | 0.00011 |
| Left PreCentral | Left Middle Temporal | 0.13 | 0.76 | 0.00011 |
| Left PreCentral | Right Parahippocampal | -0.31 | 0.62 | 0.00011 |
| Right Caudal Middle Frontal | Right Temporal Pole | -0.09 | 0.64 | 0.00016 |
| Left Rostral Middle Frontal | Left Transverse Temporal | -0.23 | 0.56 | 0.00022 |
| Left ParsTriangularis | Right Entorhinal | -0.04 | 0.69 | 0.00022 |
| Left ParsTriangularis | Left Parahippocampal | -0.15 | 0.65 | 0.00027 |
| Right Caudal Middle Frontal | Right Parahippocampal | -0.02 | 0.67 | 0.00027 |
| Left ParsTriangularis | Right Transverse Temporal | -0.03 | 0.66 | 0.00032 |
| Left Caudal Middle Frontal | Right Temporal Pole | 0.05 | 0.62 | 0.00049 |
| Left ParsOpercularis | Left Transverse Temporal | -0.23 | 0.57 | 0.00054 |
| Right PreCentral | Right Transverse Temporal | 0.29 | 0.79 | 0.00059 |
| Left ParsOpercularis | Left Parahippocampal | -0.04 | 0.60 | 0.00065 |
| Right PreCentral | Right Parahippocampal | 0.12 | 0.67 | 0.00065 |
| Left Superior Frontal | Right Entorhinal | -0.13 | 0.60 | 0.00070 |
| Left PreCentral | Right Entorhinal | -0.15 | 0.52 | 0.00070 |
| Left Orbital Frontal | Right Temporal Pole | 0.06 | 0.63 | 0.00092 |
| Left Superior Frontal | Left Transverse Temporal | 0.03 | 0.60 | 0.00097 |
| Left PreCentral | Right Transverse Temporal | 0.03 | 0.68 | 0.00124 |
| Right PreCentral | Right Banks Superior Temporal | 0.21 | 0.69 | 0.00130 |
| Left PreCentral | Right Middle Temporal | -0.03 | 0.62 | 0.00135 |
| Right CA Cingulate | Right Temporal Pole | 0.08 | 0.60 | 0.00140 |
| Left PreCentral | Right Temporal Pole | -0.19 | 0.46 | 0.00157 |
| Left ParsOpercularis | Right Entorhinal | -0.09 | 0.46 | 0.00178 |
| Left Orbital Frontal | Left Transverse Temporal | -0.09 | 0.62 | 0.00184 |
| Left PreCentral | Left Transverse Temporal | 0.06 | 0.69 | 0.00200 |
| Right PreCentral | Right Middle Temporal | 0.32 | 0.76 | 0.00216 |
| Right Superior Frontal | Right Temporal Pole | 0.14 | 0.61 | 0.00227 |
| Left PreCentral | Right Banks Superior Temporal | -0.02 | 0.56 | 0.00318 |
| Right ParsOpercularis | Right Parahippocampal | -0.19 | 0.54 | 0.00318 |
| Right Caudal Middle Frontal | Right Transverse Temporal | 0.28 | 0.77 | 0.00356 |
| Left Medial Orbital Frontal | Left Parahippocampal | -0.22 | 0.49 | 0.00372 |
| Right ParsTriangularis | Right Parahippocampal | -0.02 | 0.58 | 0.00378 |
| Left ParsOpercularis | Left Superior Temporal | 0.30 | 0.78 | 0.00432 |
| Left Caudal Middle Frontal | Left Parahippocampal | -0.13 | 0.44 | 0.00497 |
| Right PreCentral | Right Entorhinal | 0.00 | 0.58 | 0.00545 |
| Left Superior Frontal | Right Transverse Temporal | 0.00 | 0.62 | 0.00594 |
| Right ParsOpercularis | Right Middle Temporal | 0.23 | 0.71 | 0.00788 |
| Left ParsOpercularis | Right Banks Superior Temporal | -0.12 | 0.47 | 0.00794 |
| Left RA cingulate | Left Transverse Temporal | -0.24 | 0.32 | 0.00891 |
| Left ParsOpercularis | Right Transverse Temporal | -0.14 | 0.53 | 0.00945 |
| Right ParsOpercularis | Right Superior Temporal | 0.35 | 0.82 | 0.00961 |
| Right Superior Frontal | Right Parahippocampal | 0.10 | 0.65 | 0.00966 |
| Left ParsOpercularis | Left Middle Temporal | 0.35 | 0.73 | 0.01015 |
| Right ParsTriangularis | Right Transverse Temporal | 0.13 | 0.65 | 0.01053 |
| Right ParsOpercularis | Right Entorhinal | -0.05 | 0.48 | 0.01258 |
| Left Frontal Pole | Left Parahippocampal | -0.40 | 0.20 | 0.01414 |
| Left Frontal Pole | Right Parahippocampal | -0.18 | 0.35 | 0.01420 |
| Left Superior Frontal | Right Parahippocampal | -0.05 | 0.50 | 0.01441 |
| Left Rostral Middle Frontal | Right Transverse Temporal | -0.16 | 0.40 | 0.01549 |
| Left ParsTriangularis | Right Middle Temporal | 0.45 | 0.78 | 0.01582 |
| Right Rostral Middle Frontal | Right Parahippocampal | 0.02 | 0.53 | 0.01625 |
| Left Caudal Middle Frontal | Left Superior Temporal | 0.32 | 0.72 | 0.01706 |
| Right ParsOpercularis | Right Banks Superior Temporal | 0.16 | 0.55 | 0.01706 |
| Left Caudal Middle Frontal | Right Superior Temporal | 0.29 | 0.68 | 0.01943 |
| Left ParsOpercularis | Left Fusiform | 0.02 | 0.64 | 0.02078 |
| Right ParsTriangularis | Right Banks Superior Temporal | 0.28 | 0.65 | 0.02332 |
| Left Medial Orbital Frontal | Right Parahippocampal | 0.02 | 0.56 | 0.02391 |
| Right ParsTriangularis | Right Superior Temporal | 0.45 | 0.77 | 0.02397 |

| Left ParsOpercularis | Right Temporal Pole | 0.06 | 0.45 | 0.02462 |
| --- | --- | --- | --- | --- |
| Left Orbital Frontal | Left Temporal Pole | 0.19 | 0.68 | 0.02472 |
| Left Medial Orbital Frontal | Left Transverse Temporal | -0.37 | 0.16 | 0.02505 |
| Left ParsTriangularis | Left Entorhinal | 0.05 | 0.58 | 0.02548 |
| Right ParsOpercularis | Right Transverse Temporal | 0.23 | 0.67 | 0.02613 |
| Left Rostral Middle Frontal | Left Parahippocampal | -0.27 | 0.27 | 0.02796 |
| Left Frontal Pole | Left Transverse Temporal | -0.21 | 0.35 | 0.02845 |
| Left ParsTriangularis | Left Fusiform | 0.07 | 0.61 | 0.03050 |
| Left PreCentral | Right Superior Temporal | 0.01 | 0.56 | 0.03401 |
| Left Orbital Frontal | Right Entorhinal | 0.00 | 0.46 | 0.03406 |
| Left ParsTriangularis | Left Temporal Pole | -0.04 | 0.50 | 0.03563 |
| Right ParsOpercularis | Right Inferior Temporal | 0.49 | 0.73 | 0.04610 |
| Left Medial Orbital Frontal | Right Transverse Temporal | 0.01 | 0.43 | 0.04804 |
| Right ParsTriangularis | Right Middle Temporal | 0.41 | 0.70 | 0.04864 |
| Left ParsOrbitalis | Left Transverse Temporal | -0.03 | 0.38 | 0.04982 |
| Left ParsTriangularis | Right Isthmus | -0.11 | 0.75 | 0.00000 |
| Left PreCentral | Left ParaCentral Lobule | 0.24 | 0.84 | 0.00000 |
| Left PreCentral | Left Inferior Parietal | -0.04 | 0.73 | 0.00000 |
| Left PreCentral | Right ParaCentral Lobule | 0.14 | 0.77 | 0.00000 |
| Left CA Cingulate | Left Posterior Cingulate | 0.12 | 0.70 | 0.00032 |
| Right PreCentral | Right ParaCentral Lobule | 0.29 | 0.81 | 0.00049 |
| Right ParsTriangularis | Right ParaCentral Lobule | 0.33 | 0.86 | 0.00130 |
| Left ParsTriangularis | Left SupraMarginal | 0.28 | 0.74 | 0.00140 |
| Left ParsTriangularis | Left Superior Parietal | -0.06 | 0.53 | 0.00194 |
| Left PreCentral | Left Posterior Cingulate | -0.16 | 0.47 | 0.00200 |
| Left PreCentral | Right SupraMarginal | 0.07 | 0.66 | 0.00238 |
| Right Orbital Frontal | Right Superior Parietal | 0.12 | 0.72 | 0.00259 |
| Left ParsTriangularis | Right Superior Parietal | -0.01 | 0.48 | 0.00329 |
| Left ParsTriangularis | Right Inferior Parietal | 0.26 | 0.71 | 0.00372 |
| Right Rostral Middle Frontal | Right PostCentral | 0.25 | 0.72 | 0.00389 |
| Left PreCentral | Left Superior Parietal | 0.02 | 0.58 | 0.00529 |
| Right ParsTriangularis | Right Superior Parietal | 0.27 | 0.73 | 0.00599 |
| Left PreCentral | Right Superior Parietal | -0.11 | 0.47 | 0.00713 |
| Left ParsTriangularis | Left Isthmus | 0.10 | 0.66 | 0.00858 |
| Left ParsOpercularis | Right ParaCentral Lobule | 0.13 | 0.61 | 0.00874 |
| Left PreCentral | Left SupraMarginal | 0.31 | 0.72 | 0.00885 |
| Left ParsTriangularis | Right ParaCentral Lobule | 0.20 | 0.66 | 0.01112 |
| Right PreCentral | Right Superior Parietal | 0.26 | 0.66 | 0.01123 |
| Right ParsOpercularis | Right Superior Parietal | 0.41 | 0.80 | 0.01252 |
| Left Rostral Middle Frontal | Right Isthmus | 0.09 | 0.63 | 0.01279 |
| Left ParsTriangularis | Left Inferior Parietal | 0.33 | 0.69 | 0.01409 |
| Right CA Cingulate | Right Superior Parietal | -0.12 | 0.39 | 0.02111 |
| Right PreCentral | Right Isthmus | 0.11 | 0.55 | 0.02121 |
| Left ParsOpercularis | Left Posterior Cingulate | -0.10 | 0.43 | 0.02138 |
| Right RA cingulate | Right Superior Parietal | -0.29 | 0.20 | 0.02856 |
| Left PreCentral | Left Isthmus | 0.04 | 0.54 | 0.03093 |
| Left Superior Frontal | Right Superior Parietal | 0.35 | 0.73 | 0.03541 |
| Left PreCentral | Right Inferior Parietal | 0.09 | 0.59 | 0.03768 |
| Left Rostral Middle Frontal | Right Superior Parietal | -0.01 | 0.45 | 0.04453 |
| Left Orbital Frontal | Left Posterior Cingulate | 0.11 | 0.58 | 0.04621 |
| Left ParsOpercularis | Left ParaCentral Lobule | 0.13 | 0.48 | 0.04626 |
| Right Frontal Pole | Right PostCentral | -0.10 | 0.35 | 0.04713 |
| Left CA Cingulate | Right Isthmus | -0.17 | 0.39 | 0.04761 |
| Right PreCentral | Right Inferior Parietal | 0.31 | 0.68 | 0.04993 |
| Left ParsTriangularis | Right PreCuneus | 0.04 | 0.71 | 0.00059 |
| Right PreCentral | Right PreCuneus | 0.41 | 0.81 | 0.00059 |
| Left PreCentral | Right Pericalcarine | -0.29 | 0.44 | 0.00070 |
| Left PreCentral | Right PreCuneus | 0.02 | 0.68 | 0.00081 |
| Right Medial Orbital Frontal | Right Pericalcarine | -0.22 | 0.50 | 0.00081 |
| Left Caudal Middle Frontal | Left Cuneus | 0.06 | 0.66 | 0.00259 |
| Left PreCentral | Left PreCuneus | 0.21 | 0.74 | 0.00389 |
| Left ParsTriangularis | Left PreCuneus | 0.12 | 0.64 | 0.00572 |
| Right Frontal Pole | Right Cuneus | -0.07 | 0.48 | 0.01150 |
| Right RA cingulate | Right Pericalcarine | -0.34 | 0.23 | 0.01371 |
| Left ParsTriangularis | Right Lingual | 0.06 | 0.53 | 0.01398 |
| Left Superior Frontal | Right Pericalcarine | 0.16 | 0.62 | 0.01414 |

| Left PreCentral | Left Pericalcarine | -0.11 | 0.45 | 0.01641 |
| --- | --- | --- | --- | --- |
| Left ParsOpercularis | Right PreCuneus | 0.21 | 0.67 | 0.01738 |
| Right Frontal Pole | Right Lingual | -0.22 | 0.23 | 0.02240 |
| Left Superior Frontal | Left PreCuneus | 0.42 | 0.73 | 0.02343 |
| Left Rostral Middle Frontal | Right Pericalcarine | -0.06 | 0.50 | 0.02462 |
| Left CA Cingulate | Left PreCuneus | 0.09 | 0.53 | 0.02575 |
| Right PreCentral | Right Pericalcarine | 0.05 | 0.44 | 0.02575 |
| Left Caudal Middle Frontal | Right PreCuneus | 0.48 | 0.79 | 0.02580 |
| Left ParsTriangularis | Right Lateral Occipital | 0.23 | 0.63 | 0.02758 |
| Right CA Cingulate | Right Pericalcarine | -0.24 | 0.22 | 0.02861 |
| Left ParsTriangularis | Left Lingual | 0.22 | 0.62 | 0.02904 |
| Left ParsTriangularis | Left Lateral Occipital | 0.26 | 0.71 | 0.03476 |
| Right Rostral Middle Frontal | Right Cuneus | 0.09 | 0.51 | 0.03649 |
| Left ParsOpercularis | Left Lateral Occipital | 0.31 | 0.68 | 0.04135 |
| Right Orbital Frontal | Right Pericalcarine | 0.07 | 0.42 | 0.04545 |
| Right ParsOrbitalis | Right Lingual | 0.13 | 0.60 | 0.04551 |
| Left Caudal Middle Frontal | Left PreCuneus | 0.38 | 0.74 | 0.04632 |
| Left PreCentral | Right Insula | 0.04 | 0.74 | 0.00000 |
| Left ParsOpercularis | Right Insula | 0.19 | 0.83 | 0.00022 |
| Right ParsOpercularis | Right Insula | 0.33 | 0.76 | 0.01344 |
| Left Caudal Middle Frontal | Right Insula | 0.16 | 0.63 | 0.02942 |
| Left PreCentral | Left Insula | 0.11 | 0.57 | 0.03196 |
| Left ParsOpercularis | Left Insula | 0.24 | 0.69 | 0.03422 |
| Left Middle Temporal | Right PreCentral | 0.32 | 0.84 | 0.00000 |
| Left Transverse Temporal | Right ParsTriangularis | -0.34 | 0.46 | 0.00022 |
| Left Transverse Temporal | Right ParsOpercularis | -0.06 | 0.58 | 0.00130 |
| Left Parahippocampal | Right Caudal Middle Frontal | 0.06 | 0.59 | 0.00259 |
| Left Transverse Temporal | Right PreCentral | 0.00 | 0.59 | 0.00275 |
| Left Parahippocampal | Right PreCentral | 0.14 | 0.57 | 0.00664 |
| Left Entorhinal | Right ParsOpercularis | -0.06 | 0.45 | 0.01209 |
| Left Superior Temporal | Right Orbital Frontal | 0.20 | 0.62 | 0.01895 |
| Left Transverse Temporal | Right Medial Orbital Frontal | -0.21 | 0.23 | 0.02089 |
| Left Parahippocampal | Right Superior Frontal | 0.10 | 0.58 | 0.02748 |
| Left Transverse Temporal | Right Superior Frontal | 0.15 | 0.57 | 0.02883 |
| Left Temporal Pole | Right PreCentral | -0.15 | 0.38 | 0.02985 |
| Left Transverse Temporal | Right Frontal Pole | -0.33 | 0.19 | 0.03190 |
| Left Superior Temporal | Right ParsTriangularis | 0.38 | 0.70 | 0.03655 |
| Left Fusiform | Right RA cingulate | 0.06 | 0.57 | 0.03962 |
| Left Temporal Pole | Right ParsOpercularis | -0.08 | 0.39 | 0.04194 |
| Left Entorhinal | Right Caudal Middle Frontal | -0.04 | 0.46 | 0.04885 |
| Left ParaCentral Lobule | Right PreCentral | 0.28 | 0.77 | 0.00130 |
| Left Inferior Parietal | Right PreCentral | 0.40 | 0.82 | 0.00146 |
| Left SupraMarginal | Right PreCentral | 0.52 | 0.81 | 0.00459 |
| Left SupraMarginal | Right ParsTriangularis | 0.32 | 0.71 | 0.00540 |
| Left Isthmus | Right CA Cingulate | 0.07 | 0.64 | 0.00567 |
| Left Posterior Cingulate | Right Medial Orbital Frontal | 0.37 | -0.11 | 0.01069 |
| Left Superior Parietal | Right PreCentral | 0.11 | 0.62 | 0.01069 |
| Left Isthmus | Right Orbital Frontal | 0.17 | 0.68 | 0.01420 |
| Left PostCentral | Right Superior Frontal | 0.40 | 0.74 | 0.01641 |
| Right ParaCentral Lobule | Right RA cingulate | 0.30 | -0.20 | 0.02478 |
| Left ParaCentral Lobule | Right ParsTriangularis | 0.22 | 0.66 | 0.02645 |
| Left PostCentral | Right Frontal Pole | -0.15 | 0.30 | 0.02845 |
| Left Superior Parietal | Right ParsTriangularis | 0.30 | 0.72 | 0.02888 |
| Left PreCuneus | Right PreCentral | 0.31 | 0.79 | 0.00011 |
| Left Pericalcarine | Right RA cingulate | -0.27 | 0.25 | 0.00686 |
| Left Pericalcarine | Right CA Cingulate | -0.13 | 0.43 | 0.00815 |
| Left Lingual | Right ParsTriangularis | 0.30 | 0.68 | 0.03099 |
| Left Lingual | Right Rostral Middle Frontal | 0.42 | 0.73 | 0.03120 |
| Left Fusiform | Left Transverse Temporal | -0.09 | 0.63 | 0.00000 |
| Left Banks Superior Temporal | Right Parahippocampal | -0.28 | 0.51 | 0.00000 |
| Right Parahippocampal | Right Banks Superior Temporal | -0.21 | 0.62 | 0.00000 |
| Left Transverse Temporal | Right Temporal Pole | -0.28 | 0.55 | 0.00005 |
| Left Middle Temporal | Right Transverse Temporal | 0.01 | 0.66 | 0.00011 |
| Right Parahippocampal | Right Transverse Temporal | 0.05 | 0.77 | 0.00027 |
| Right Temporal Pole | Right Inferior Temporal | 0.24 | 0.74 | 0.00032 |
| Left Transverse Temporal | Right Inferior Temporal | -0.01 | 0.72 | 0.00038 |

| Right Middle Temporal | Right Banks Superior Temporal | 0.25 | 0.83 | 0.00043 |
| --- | --- | --- | --- | --- |
| Left Middle Temporal | Right Parahippocampal | -0.09 | 0.68 | 0.00108 |
| Right Parahippocampal | Right Superior Temporal | 0.05 | 0.68 | 0.00119 |
| Left Superior Temporal | Right Parahippocampal | -0.11 | 0.64 | 0.00124 |
| Left Banks Superior Temporal | Right Middle Temporal | 0.31 | 0.81 | 0.00124 |
| Left Middle Temporal | Right Temporal Pole | 0.10 | 0.60 | 0.00140 |
| Left Parahippocampal | Left Middle Temporal | -0.01 | 0.60 | 0.00194 |
| Right Temporal Pole | Right Superior Temporal | 0.18 | 0.61 | 0.00356 |
| Right Middle Temporal | Right Transverse Temporal | 0.18 | 0.67 | 0.00383 |
| Left Inferior Temporal | Right Banks Superior Temporal | 0.07 | 0.60 | 0.00410 |
| Left Fusiform | Right Banks Superior Temporal | 0.12 | 0.63 | 0.00421 |
| Left Parahippocampal | Right Banks Superior Temporal | -0.07 | 0.50 | 0.00529 |
| Left Banks Superior Temporal | Right Banks Superior Temporal | 0.50 | 0.85 | 0.00615 |
| Left Middle Temporal | Right Middle Temporal | 0.59 | 0.87 | 0.00675 |
| Left Superior Temporal | Right Temporal Pole | 0.21 | 0.68 | 0.00858 |
| Right Temporal Pole | Right Middle Temporal | 0.13 | 0.56 | 0.01009 |
| Left Inferior Temporal | Left Banks Superior Temporal | 0.21 | 0.65 | 0.01166 |
| Left Middle Temporal | Right Entorhinal | 0.19 | 0.64 | 0.01188 |
| Left Parahippocampal | Left Banks Superior Temporal | -0.10 | 0.46 | 0.01242 |
| Left Middle Temporal | Right Banks Superior Temporal | 0.35 | 0.74 | 0.01323 |
| Left Transverse Temporal | Right Entorhinal | -0.01 | 0.42 | 0.01393 |
| Right Entorhinal | Right Inferior Temporal | 0.12 | 0.52 | 0.01603 |
| Left Parahippocampal | Left Temporal Pole | -0.13 | 0.42 | 0.01657 |
| Left Banks Superior Temporal | Right Transverse Temporal | 0.13 | 0.56 | 0.01798 |
| Left Superior Temporal | Right Superior Temporal | 0.63 | 0.84 | 0.01857 |
| Left Inferior Temporal | Right Entorhinal | 0.18 | 0.57 | 0.01906 |
| Left Fusiform | Right Entorhinal | -0.08 | 0.54 | 0.02229 |
| Left Middle Temporal | Left Transverse Temporal | 0.00 | 0.53 | 0.02310 |
| Left Superior Temporal | Right Entorhinal | -0.02 | 0.44 | 0.02596 |
| Left Entorhinal | Left Transverse Temporal | 0.00 | 0.53 | 0.02775 |
| Right Entorhinal | Right Fusiform | 0.39 | 0.69 | 0.02807 |
| Left Middle Temporal | Left Banks Superior Temporal | 0.38 | 0.73 | 0.03039 |
| Left Transverse Temporal | Right Middle Temporal | 0.03 | 0.53 | 0.03196 |
| Right Inferior Temporal | Right Banks Superior Temporal | 0.22 | 0.64 | 0.03331 |
| Right Fusiform | Right Banks Superior Temporal | 0.41 | 0.72 | 0.03584 |
| Left Inferior Temporal | Right Temporal Pole | 0.15 | 0.57 | 0.03779 |
| Left Banks Superior Temporal | Right Entorhinal | -0.22 | 0.40 | 0.03789 |
| Left Parahippocampal | Right Middle Temporal | 0.07 | 0.48 | 0.03973 |
| Right Inferior Temporal | Right Transverse Temporal | 0.26 | 0.62 | 0.04076 |
| Left Superior Temporal | Left Transverse Temporal | -0.01 | 0.48 | 0.04151 |
| Left Entorhinal | Left Parahippocampal | 0.09 | 0.54 | 0.04659 |
| Right Parahippocampal | Right Temporal Pole | -0.02 | 0.45 | 0.04713 |
| Left Entorhinal | Left Banks Superior Temporal | 0.01 | 0.53 | 0.04740 |
| Right Temporal Pole | Right SupraMarginal | -0.08 | 0.60 | 0.00005 |
| Right Temporal Pole | Right Superior Parietal | -0.29 | 0.63 | 0.00005 |
| Right Middle Temporal | Right Superior Parietal | -0.03 | 0.67 | 0.00005 |
| Right Parahippocampal | Right SupraMarginal | 0.03 | 0.69 | 0.00032 |
| Left Parahippocampal | Left SupraMarginal | -0.09 | 0.57 | 0.00043 |
| Right Transverse Temporal | Right SupraMarginal | 0.29 | 0.81 | 0.00043 |
| Right Middle Temporal | Right SupraMarginal | 0.37 | 0.82 | 0.00076 |
| Left Transverse Temporal | Right SupraMarginal | -0.11 | 0.53 | 0.00092 |
| Right Middle Temporal | Right Inferior Parietal | 0.43 | 0.87 | 0.00103 |
| Right Temporal Pole | Right Inferior Parietal | -0.03 | 0.60 | 0.00151 |
| Left Superior Temporal | Left SupraMarginal | 0.31 | 0.79 | 0.00189 |
| Left Middle Temporal | Left SupraMarginal | 0.46 | 0.80 | 0.00265 |
| Left Banks Superior Temporal | Right Inferior Parietal | 0.38 | 0.79 | 0.00270 |
| Left Parahippocampal | Right SupraMarginal | -0.02 | 0.57 | 0.00313 |
| Left Superior Temporal | Right Isthmus | 0.14 | 0.64 | 0.00340 |
| Left Parahippocampal | Right ParaCentral Lobule | -0.10 | 0.48 | 0.00389 |
| Left Middle Temporal | Left Inferior Parietal | 0.49 | 0.81 | 0.00583 |
| Right Entorhinal | Right Inferior Parietal | 0.13 | 0.65 | 0.00707 |
| Right Entorhinal | Right Superior Parietal | -0.28 | 0.31 | 0.00713 |
| Right Temporal Pole | Right PostCentral | -0.34 | 0.38 | 0.00982 |
| Right Banks Superior Temporal | Right SupraMarginal | 0.41 | 0.73 | 0.00982 |
| Left Transverse Temporal | Left Inferior Parietal | 0.10 | 0.63 | 0.01036 |
| Left Middle Temporal | Right Inferior Parietal | 0.53 | 0.82 | 0.01042 |

| Left Fusiform | Right Inferior Parietal | 0.45 | 0.83 | 0.01080 |
| --- | --- | --- | --- | --- |
| Left Entorhinal | Left Inferior Parietal | -0.05 | 0.54 | 0.01236 |
| Right Inferior Temporal | Right Superior Parietal | 0.14 | 0.56 | 0.01328 |
| Left Superior Temporal | Right Inferior Parietal | 0.47 | 0.80 | 0.01382 |
| Left Temporal Pole | Right Superior Parietal | -0.04 | 0.47 | 0.01393 |
| Left Transverse Temporal | Left SupraMarginal | 0.08 | 0.60 | 0.01484 |
| Left Superior Temporal | Right Posterior Cingulate | -0.19 | 0.38 | 0.01722 |
| Left Temporal Pole | Left Inferior Parietal | 0.08 | 0.57 | 0.01841 |
| Left Middle Temporal | Right Superior Parietal | 0.19 | 0.57 | 0.01857 |
| Left Banks Superior Temporal | Left SupraMarginal | 0.36 | 0.74 | 0.01857 |
| Left Parahippocampal | Right Isthmus | 0.10 | 0.52 | 0.01997 |
| Left Superior Temporal | Left Superior Parietal | 0.18 | 0.67 | 0.02251 |
| Left Entorhinal | Right Inferior Parietal | 0.23 | 0.65 | 0.02510 |
| Left Middle Temporal | Right Isthmus | 0.08 | 0.53 | 0.02510 |
| Left Inferior Temporal | Right Inferior Parietal | 0.43 | 0.72 | 0.02721 |
| Right Superior Temporal | Right SupraMarginal | 0.47 | 0.80 | 0.02785 |
| Left Fusiform | Left Inferior Parietal | 0.32 | 0.72 | 0.03304 |
| Right Superior Temporal | Right Inferior Parietal | 0.47 | 0.81 | 0.03703 |
| Left Superior Temporal | Left PostCentral | 0.26 | 0.64 | 0.04399 |
| Left Superior Temporal | Right Lingual | -0.12 | 0.66 | 0.00000 |
| Left Middle Temporal | Left PreCuneus | 0.32 | 0.87 | 0.00005 |
| Right Temporal Pole | Right Lingual | -0.22 | 0.42 | 0.00011 |
| Left Superior Temporal | Left Lingual | 0.35 | 0.80 | 0.00135 |
| Right Temporal Pole | Right Lateral Occipital | -0.33 | 0.39 | 0.00178 |
| Left Transverse Temporal | Left Pericalcarine | -0.02 | 0.62 | 0.00243 |
| Left Middle Temporal | Right PreCuneus | 0.40 | 0.84 | 0.00248 |
| Left Temporal Pole | Right Lingual | -0.03 | 0.48 | 0.00389 |
| Left Banks Superior Temporal | Right Lingual | 0.09 | 0.65 | 0.00459 |
| Right Superior Temporal | Right Lingual | 0.19 | 0.76 | 0.00583 |
| Right Temporal Pole | Right PreCuneus | 0.09 | 0.60 | 0.00848 |
| Right Inferior Temporal | Right Pericalcarine | -0.11 | 0.51 | 0.00961 |
| Right Temporal Pole | Right Pericalcarine | 0.07 | 0.60 | 0.00988 |
| Right Transverse Temporal | Right PreCuneus | 0.35 | 0.72 | 0.01004 |
| Left Entorhinal | Right Lingual | 0.03 | 0.48 | 0.01015 |
| Right Parahippocampal | Right PreCuneus | 0.27 | 0.70 | 0.01269 |
| Right Entorhinal | Right Lateral Occipital | 0.04 | 0.46 | 0.01484 |
| Left Superior Temporal | Right PreCuneus | 0.23 | 0.66 | 0.01630 |
| Right Entorhinal | Right Lingual | 0.06 | 0.50 | 0.01652 |
| Left Banks Superior Temporal | Left Lateral Occipital | 0.21 | 0.58 | 0.02035 |
| Left Banks Superior Temporal | Left Lingual | 0.04 | 0.46 | 0.02165 |
| Left Temporal Pole | Left PreCuneus | 0.02 | 0.62 | 0.02235 |
| Left Banks Superior Temporal | Right Lateral Occipital | 0.20 | 0.58 | 0.02569 |
| Right Banks Superior Temporal | Right Lingual | 0.04 | 0.52 | 0.02596 |
| Right Inferior Temporal | Right Lingual | 0.15 | 0.53 | 0.02877 |
| Left Superior Temporal | Right Lateral Occipital | 0.28 | 0.67 | 0.03039 |
| Left Transverse Temporal | Right Pericalcarine | 0.21 | 0.59 | 0.03395 |
| Left Fusiform | Right Lingual | 0.19 | 0.63 | 0.03590 |
| Left Banks Superior Temporal | Left PreCuneus | 0.23 | 0.62 | 0.03973 |
| Right Transverse Temporal | Right Lingual | -0.07 | 0.44 | 0.04291 |
| Right Middle Temporal | Right Lingual | 0.25 | 0.65 | 0.04313 |
| Left Entorhinal | Right PreCuneus | 0.09 | 0.59 | 0.04545 |
| Left Superior Temporal | Left PreCuneus | 0.30 | 0.68 | 0.04972 |
| Left Superior Temporal | Right Insula | 0.39 | 0.82 | 0.00216 |
| Left Transverse Temporal | Right Insula | -0.10 | 0.60 | 0.00345 |
| Left SupraMarginal | Right Parahippocampal | -0.18 | 0.68 | 0.00000 |
| Left SupraMarginal | Right Temporal Pole | -0.20 | 0.63 | 0.00000 |
| Right Isthmus | Right Temporal Pole | -0.03 | 0.58 | 0.00043 |
| Left Superior Parietal | Right Temporal Pole | -0.22 | 0.60 | 0.00076 |
| Right Isthmus | Right Parahippocampal | -0.11 | 0.57 | 0.00130 |
| Left ParaCentral Lobule | Right Temporal Pole | -0.08 | 0.55 | 0.00146 |
| Left PostCentral | Right Temporal Pole | -0.19 | 0.53 | 0.00162 |
| Left Inferior Parietal | Right Temporal Pole | -0.08 | 0.52 | 0.00173 |
| Left Isthmus | Right Entorhinal | 0.14 | 0.64 | 0.00297 |
| Left SupraMarginal | Right Middle Temporal | 0.45 | 0.86 | 0.00464 |
| Left Inferior Parietal | Right Transverse Temporal | 0.15 | 0.69 | 0.00470 |
| Left Superior Parietal | Right Middle Temporal | 0.15 | 0.70 | 0.00529 |

| Right ParaCentral Lobule | Right Temporal Pole | -0.01 | 0.53 | 0.00588 |
| --- | --- | --- | --- | --- |
| Left SupraMarginal | Right Transverse Temporal | 0.23 | 0.68 | 0.00718 |
| Left Superior Parietal | Right Inferior Temporal | 0.21 | 0.66 | 0.00858 |
| Left Inferior Parietal | Right Entorhinal | 0.03 | 0.55 | 0.01015 |
| Left SupraMarginal | Right Entorhinal | 0.04 | 0.48 | 0.01090 |
| Left ParaCentral Lobule | Left Parahippocampal | -0.10 | 0.41 | 0.01128 |
| Right ParaCentral Lobule | Right Parahippocampal | 0.11 | 0.61 | 0.01139 |
| Left SupraMarginal | Right Banks Superior Temporal | 0.36 | 0.74 | 0.01182 |
| Left ParaCentral Lobule | Right Inferior Temporal | 0.32 | 0.68 | 0.01215 |
| Right Isthmus | Right Entorhinal | 0.01 | 0.49 | 0.01252 |
| Left ParaCentral Lobule | Right Parahippocampal | -0.13 | 0.45 | 0.01312 |
| Left Inferior Parietal | Right Parahippocampal | 0.04 | 0.53 | 0.01387 |
| Left Posterior Cingulate | Left Parahippocampal | -0.21 | 0.27 | 0.01900 |
| Left Inferior Parietal | Right Banks Superior Temporal | 0.43 | 0.76 | 0.02051 |
| Left Superior Parietal | Right Superior Temporal | 0.29 | 0.71 | 0.02505 |
| Left Isthmus | Right Temporal Pole | 0.07 | 0.48 | 0.03055 |
| Left SupraMarginal | Right Inferior Temporal | 0.41 | 0.74 | 0.03142 |
| Left SupraMarginal | Right Superior Temporal | 0.50 | 0.83 | 0.03293 |
| Left Superior Parietal | Right Parahippocampal | -0.01 | 0.44 | 0.03520 |
| Left Isthmus | Left Entorhinal | -0.03 | 0.41 | 0.03671 |
| Left Inferior Parietal | Right Middle Temporal | 0.50 | 0.80 | 0.03757 |
| Left Lingual | Right Temporal Pole | -0.17 | 0.62 | 0.00011 |
| Left Cuneus | Right Temporal Pole | -0.15 | 0.54 | 0.00054 |
| Left Lateral Occipital | Right Temporal Pole | -0.26 | 0.43 | 0.00059 |
| Left Pericalcarine | Right Temporal Pole | -0.12 | 0.63 | 0.00081 |
| Left PreCuneus | Right Middle Temporal | 0.15 | 0.75 | 0.00108 |
| Left PreCuneus | Right Entorhinal | -0.23 | 0.46 | 0.00173 |
| Left PreCuneus | Right Temporal Pole | -0.14 | 0.49 | 0.00421 |
| Left PreCuneus | Right Transverse Temporal | 0.23 | 0.65 | 0.00448 |
| Left PreCuneus | Right Banks Superior Temporal | 0.27 | 0.70 | 0.00572 |
| Left PreCuneus | Right Parahippocampal | -0.06 | 0.55 | 0.00702 |
| Left Pericalcarine | Right Inferior Temporal | 0.18 | 0.60 | 0.00821 |
| Left Lateral Occipital | Right Entorhinal | 0.00 | 0.49 | 0.00907 |
| Left Lateral Occipital | Right Middle Temporal | 0.48 | 0.77 | 0.03514 |
| Left Insula | Left Transverse Temporal | -0.30 | 0.48 | 0.00065 |
| Right Insula | Right Temporal Pole | 0.09 | 0.55 | 0.00993 |
| Left Insula | Right Temporal Pole | 0.16 | 0.55 | 0.02235 |
| Left Insula | Right Banks Superior Temporal | -0.04 | 0.44 | 0.02596 |
| Left SupraMarginal | Right SupraMarginal | 0.46 | 0.92 | 0.00000 |
| Left SupraMarginal | Right ParaCentral Lobule | 0.33 | 0.67 | 0.00605 |
| Left SupraMarginal | Right Inferior Parietal | 0.51 | 0.83 | 0.01868 |
| Left PostCentral | Left SupraMarginal | 0.32 | 0.74 | 0.02715 |
| Left SupraMarginal | Left PreCuneus | 0.37 | 0.79 | 0.00227 |
| Left Superior Parietal | Left Cuneus | 0.48 | 0.82 | 0.00540 |
| Left ParaCentral Lobule | Left PreCuneus | 0.40 | 0.76 | 0.00561 |
| Right Inferior Parietal | Right Lingual | 0.34 | 0.75 | 0.02397 |
| Left Inferior Parietal | Left PreCuneus | 0.63 | 0.84 | 0.02947 |
| Right SupraMarginal | Right PreCuneus | 0.49 | 0.78 | 0.03957 |
| Right Isthmus | Right Insula | 0.14 | 0.76 | 0.00059 |
| Left Posterior Cingulate | Right Insula | 0.14 | 0.61 | 0.00567 |
| Left ParaCentral Lobule | Right Insula | 0.25 | 0.68 | 0.01161 |
| Right Posterior Cingulate | Right Insula | 0.01 | 0.60 | 0.01576 |
| Right ParaCentral Lobule | Right Insula | 0.37 | 0.65 | 0.02094 |
| Left Posterior Cingulate | Left Insula | 0.02 | 0.51 | 0.03293 |
| Left PreCuneus | Right SupraMarginal | 0.37 | 0.69 | 0.02094 |
| Left PreCuneus | Right PreCuneus | 0.60 | 0.86 | 0.00675 |
| Left Lateral Occipital | Right Lateral Occipital | 0.87 | 0.67 | 0.03347 |
| Left Pericalcarine | Right Insula | 0.32 | 0.64 | 0.04885 |

**Table S10.** Group differences in the strength of inter-regional correlations in cortical thickness between youths with childhood-onset conduct disorder (CO-CD) and adolescence-onset conduct disorder (AO-CD) in the Cambridge sample (N=83) *when not* including any covariates in the statistical model.

|  |  |  |  |  |  |
| --- | --- | --- | --- | --- | --- |
|  |  | **Metric** | **Metric** |  |  |
| **Region1** | **Region2** | **CO-CD** | **AO-CD** | **Corr P values** |  |
| Left Orbital Frontal | Left PreCentral | 0.57 | 0.04 | 0.00478 | **frontal cortices** |
| Right Orbital Frontal | Right PreCentral | 0.66 | 0.02 | 0.00534 | **temporal cortices** |
| Left Orbital Frontal | Right PreCentral | 0.70 | 0.08 | 0.01097 | **parietal cortices** |
| Left Orbital Frontal | Right ParsTriangularis | 0.68 | 0.21 | 0.02531 | **occipital cortices** |
| Left Orbital Frontal | Left Frontal Pole | 0.39 | -0.08 | 0.02981 |  |
| Left Rostral Middle Frontal | Right ParsOpercularis | 0.60 | 0.17 | 0.03065 |  |
| Right ParsOrbitalis | Right Frontal Pole | 0.38 | -0.14 | 0.04612 |  |
| Left ParsOrbitalis | Left Superior Temporal | 0.49 | -0.22 | 0.00309 |  |
| Left ParsOpercularis | Left Middle Temporal | 0.71 | 0.09 | 0.00422 |  |
| Left PreCentral | Right Temporal Pole | 0.51 | -0.09 | 0.01744 |  |
| Right PreCentral | Right Middle Temporal | 0.61 | 0.08 | 0.01856 |  |
| Left Medial Orbital Frontal | Left Parahippocampal | -0.03 | 0.59 | 0.02137 |  |
| Left Orbital Frontal | Left Middle Temporal | 0.55 | 0.07 | 0.02475 |  |
| Left Orbital Frontal | Left Superior Temporal | 0.60 | 0.15 | 0.02812 |  |
| Left Orbital Frontal | Right Parahippocampal | 0.41 | -0.13 | 0.03150 |  |
| Left Orbital Frontal | Right Transverse Temporal | 0.38 | -0.11 | 0.04022 |  |
| Left ParsTriangularis | Right Middle Temporal | 0.63 | 0.19 | 0.04134 |  |
| Right ParsOrbitalis | Right Parahippocampal | 0.47 | -0.08 | 0.04809 |  |
| Right PreCentral | Right PostCentral | 0.75 | -0.01 | 0.00169 |  |
| Left Caudal Middle Frontal | Right Isthmus | -0.08 | 0.58 | 0.00450 |  |
| Left PreCentral | Right PostCentral | 0.69 | 0.11 | 0.00562 |  |
| Left Orbital Frontal | Right ParaCentral Lobule | 0.60 | 0.02 | 0.00647 |  |
| Right Superior Frontal | Right Isthmus | 0.17 | 0.62 | 0.02053 |  |
| Left Orbital Frontal | Right PostCentral | 0.58 | 0.03 | 0.04219 |  |
| Right Frontal Pole | Right Pericalcarine | 0.29 | -0.30 | 0.02306 |  |
| Right RA cingulate | Right Pericalcarine | -0.22 | 0.36 | 0.02334 |  |
| Right Frontal Pole | Right Lateral Occipital | 0.50 | -0.04 | 0.03459 |  |
| Left Orbital Frontal | Right Insula | 0.60 | 0.02 | 0.00731 |  |
| Left Middle Temporal | Right CA Cingulate | 0.05 | 0.63 | 0.01097 |  |
| Left Middle Temporal | Right ParsTriangularis | 0.60 | 0.09 | 0.02362 |  |
| Left Transverse Temporal | Right Caudal Middle Frontal | 0.17 | 0.58 | 0.03459 |  |
| Left Middle Temporal | Right PreCentral | 0.59 | 0.22 | 0.04612 |  |
| Left SupraMarginal | Right CA Cingulate | 0.16 | 0.61 | 0.01237 |  |
| Left ParaCentral Lobule | Right Caudal Middle Frontal | 0.69 | 0.23 | 0.01406 |  |
| Left Superior Parietal | Right Frontal Pole | 0.49 | 0.00 | 0.02840 |  |
| Left Superior Temporal | Left Inferior Temporal | 0.67 | 0.05 | 0.00000 |  |
| Left Temporal Pole | Right Superior Temporal | 0.60 | -0.05 | 0.00028 |  |
| Left Middle Temporal | Right Superior Temporal | 0.68 | 0.02 | 0.00028 |  |
| Right Parahippocampal | Right Temporal Pole | 0.56 | -0.22 | 0.00084 |  |
| Left Superior Temporal | Right Temporal Pole | 0.60 | -0.12 | 0.00197 |  |
| Left Superior Temporal | Right Superior Temporal | 0.85 | 0.48 | 0.00281 |  |
| Left Fusiform | Left Inferior Temporal | 0.73 | 0.19 | 0.00366 |  |
| Left Middle Temporal | Right Middle Temporal | 0.70 | 0.22 | 0.00534 |  |
| Left Banks Superior Temporal | Right Temporal Pole | 0.06 | -0.55 | 0.00703 |  |
| Left Inferior Temporal | Left Transverse Temporal | 0.43 | -0.16 | 0.00787 |  |
| Left Superior Temporal | Left Middle Temporal | 0.71 | 0.13 | 0.00928 |  |
| Left Inferior Temporal | Right Entorhinal | 0.38 | -0.17 | 0.01406 |  |
| Right Temporal Pole | Right Superior Temporal | 0.63 | 0.13 | 0.01519 |  |
| Right Temporal Pole | Right Fusiform | 0.47 | -0.20 | 0.01603 |  |
| Right Entorhinal | Right Banks Superior Temporal | 0.33 | -0.16 | 0.01659 |  |
| Left Middle Temporal | Right Temporal Pole | 0.51 | -0.05 | 0.01744 |  |
| Left Superior Temporal | Right Middle Temporal | 0.60 | 0.11 | 0.02334 |  |
| Left Temporal Pole | Right Inferior Temporal | 0.59 | 0.13 | 0.03881 |  |
| Left Temporal Pole | Left Superior Temporal | 0.52 | 0.01 | 0.04978 |  |
| Right Temporal Pole | Right PostCentral | 0.53 | -0.26 | 0.00028 |  |
| Left Temporal Pole | Right PostCentral | 0.43 | -0.22 | 0.02250 |  |
| Left Temporal Pole | Right Cuneus | 0.41 | -0.22 | 0.01266 |  |
| Right Temporal Pole | Right Lateral Occipital | 0.46 | -0.09 | 0.02053 |  |
| Right Temporal Pole | Right PreCuneus | 0.49 | -0.04 | 0.03431 |  |
| Left Middle Temporal | Right Insula | 0.47 | -0.02 | 0.04584 |  |
| Left Isthmus | Right Entorhinal | -0.34 | 0.41 | 0.00112 |  |

| Left PostCentral | Right Temporal Pole | 0.45 | -0.21 | 0.00506 |
| --- | --- | --- | --- | --- |
| Left Isthmus | Left Temporal Pole | -0.13 | 0.50 | 0.00562 |
| Right Isthmus | Right Entorhinal | -0.19 | 0.44 | 0.01012 |
| Left Isthmus | Left Entorhinal | -0.12 | 0.46 | 0.01097 |
| Left Isthmus | Right Temporal Pole | -0.09 | 0.52 | 0.01659 |
| Right Isthmus | Right Inferior Temporal | -0.09 | 0.54 | 0.01969 |
| Right Isthmus | Right Fusiform | 0.02 | 0.62 | 0.02306 |
| Left Cuneus | Right Temporal Pole | 0.32 | -0.40 | 0.00141 |
| Left Lateral Occipital | Right Temporal Pole | 0.57 | -0.05 | 0.00309 |
| Left Cuneus | Right Entorhinal | 0.40 | -0.18 | 0.03319 |
| Right Insula | Right Temporal Pole | 0.50 | -0.13 | 0.00956 |
| Left Insula | Right Temporal Pole | 0.47 | -0.15 | 0.02953 |
| Left SupraMarginal | Right Isthmus | 0.07 | 0.59 | 0.00759 |
| Left Superior Parietal | Right ParaCentral Lobule | 0.79 | 0.36 | 0.03009 |
| Left PostCentral | Right PostCentral | 0.85 | 0.50 | 0.03094 |
| Left Superior Parietal | Right PostCentral | 0.71 | 0.24 | 0.04078 |
| Left Isthmus | Right PostCentral | 0.24 | -0.23 | 0.04219 |
| Right Isthmus | Right Lingual | -0.14 | 0.49 | 0.00422 |
| Left Lingual | Right Isthmus | -0.19 | 0.41 | 0.00422 |
| Left PreCuneus | Left Lingual | 0.38 | 0.79 | 0.00647 |

| **Table S11.** Group differences in the strength of inter-regional correlations in cortical thickness between healthy controls (HCs) and youths with adolescence-onset conduct disorder (AO-CD) in the Cambridge sample (N=83) *when not* including any covariates in the statistical model. | | | | |  |
| --- | --- | --- | --- | --- | --- |
|  |  |  |  |  |  |
|  |  | **Metric** | **Metric** |  |  |
| **Region1** | **Region2** | **HCs** | **AO-CD** | **Corr P values** |  |
| Left ParsOrbitalis | Left CA Cingulate | 0.18 | -0.39 | 0.00380 | **frontal cortices** |
| Right Frontal Pole | Right CA Cingulate | -0.10 | 0.58 | 0.00418 | **temporal cortices** |
| Right PreCentral | Right CA Cingulate | -0.30 | 0.37 | 0.00570 | **parietal cortices** |
| Left Rostral Middle Frontal | Right CA Cingulate | -0.14 | 0.47 | 0.01709 | **occipital cortices** |
| Left ParsOrbitalis | Right PreCentral | 0.55 | -0.01 | 0.02240 |  |
| Right ParsOrbitalis | Right Inferior Temporal | -0.10 | 0.57 | 0.00418 |  |
| Left Orbital Frontal | Left Banks Superior Temporal | 0.45 | -0.23 | 0.00456 |  |
| Left PreCentral | Right Middle Temporal | 0.63 | 0.03 | 0.00911 |  |
| Left Rostral Middle Frontal | Left Parahippocampal | 0.02 | 0.56 | 0.00949 |  |
| Right PreCentral | Right Middle Temporal | 0.60 | 0.08 | 0.02582 |  |
| Right RA cingulate | Right Banks Superior Temporal | -0.13 | 0.45 | 0.02923 |  |
| Left CA Cingulate | Right Inferior Temporal | 0.44 | -0.08 | 0.03759 |  |
| Left RA cingulate | Left Isthmus | -0.14 | 0.62 | 0.00190 |  |
| Left RA cingulate | Right Isthmus | -0.27 | 0.51 | 0.00304 |  |
| Left Medial Orbital Frontal | Left Isthmus | -0.16 | 0.43 | 0.00797 |  |
| Right Superior Frontal | Right Isthmus | 0.03 | 0.62 | 0.00835 |  |
| Left CA Cingulate | Right Inferior Parietal | 0.43 | -0.07 | 0.00911 |  |
| Left Rostral Middle Frontal | Right Isthmus | -0.20 | 0.40 | 0.02620 |  |
| Right Medial Orbital Frontal | Right Isthmus | 0.10 | 0.65 | 0.03265 |  |
| Right RA cingulate | Right Isthmus | -0.22 | 0.42 | 0.03607 |  |
| Left Superior Frontal | Right SupraMarginal | 0.47 | 0.81 | 0.03645 |  |
| Right RA cingulate | Right Lingual | -0.24 | 0.48 | 0.00683 |  |
| Left Caudal Middle Frontal | Right Pericalcarine | -0.12 | 0.41 | 0.00835 |  |
| Right RA cingulate | Right Cuneus | -0.22 | 0.45 | 0.01101 |  |
| Left Caudal Middle Frontal | Right Lingual | 0.01 | 0.65 | 0.01822 |  |
| Left ParsOrbitalis | Right Lingual | -0.31 | 0.30 | 0.02164 |  |
| Right RA cingulate | Right Pericalcarine | -0.25 | 0.36 | 0.03645 |  |
| Left CA Cingulate | Left Insula | 0.71 | 0.13 | 0.04100 |  |
| Left Banks Superior Temporal | Right Orbital Frontal | 0.55 | -0.10 | 0.00076 |  |
| Left Banks Superior Temporal | Right ParsTriangularis | 0.41 | -0.17 | 0.01291 |  |
| Left Middle Temporal | Right ParsTriangularis | 0.69 | 0.09 | 0.01329 |  |
| Left Middle Temporal | Right CA Cingulate | 0.01 | 0.63 | 0.02734 |  |
| Left Banks Superior Temporal | Right ParsOrbitalis | 0.30 | -0.29 | 0.04860 |  |
| Left Isthmus | Right CA Cingulate | -0.06 | 0.49 | 0.02392 |  |
| Left Middle Temporal | Right Middle Temporal | 0.77 | 0.22 | 0.00304 |  |
| Left Superior Temporal | Left Inferior Temporal | 0.60 | 0.05 | 0.01974 |  |
| Left Fusiform | Left Superior Temporal | 0.63 | 0.15 | 0.02544 |  |
| Left Middle Temporal | Right Superior Temporal | 0.54 | 0.02 | 0.02620 |  |

| Left Entorhinal | Left Inferior Temporal | -0.37 | 0.22 | 0.02772 |  |
| --- | --- | --- | --- | --- | --- |
| Left Banks Superior Temporal | Right Temporal Pole | 0.07 | -0.55 | 0.03987 |  |
| Left Fusiform | Left Middle Temporal | 0.71 | 0.35 | 0.04556 |  |
| Left Banks Superior Temporal | Right Superior Parietal | 0.70 | 0.16 | 0.00152 |  |
| Left Entorhinal | Right Isthmus | -0.42 | 0.30 | 0.00190 |  |
| Left Banks Superior Temporal | Left Inferior Parietal | 0.67 | -0.01 | 0.00797 |  |
| Left Parahippocampal | Right ParaCentral Lobule | -0.06 | 0.40 | 0.02772 |  |
| Left Transverse Temporal | Right Insula | -0.07 | 0.58 | 0.04024 |  |
| Left Isthmus | Left Entorhinal | -0.51 | 0.46 | 0.00000 |  |
| Left Posterior Cingulate | Right Temporal Pole | -0.05 | 0.55 | 0.02202 |  |
| Right Isthmus | Right SupraMarginal | -0.21 | 0.55 | 0.00190 |  |
| Left SupraMarginal | Right Isthmus | -0.09 | 0.59 | 0.01746 |  |
| Left ParaCentral Lobule | Left Pericalcarine | 0.52 | 0.04 | 0.01215 |  |
| Left ParaCentral Lobule | Right Insula | 0.58 | 0.06 | 0.00987 |  |
| Left PreCuneus | Right SupraMarginal | 0.31 | 0.75 | 0.01633 |  |
| Left Lingual | Right SupraMarginal | 0.01 | 0.64 | 0.03189 |  |
| Left Insula | Left PostCentral | -0.16 | 0.59 | 0.01709 |  |
| Left Insula | Left SupraMarginal | 0.09 | 0.64 | 0.02999 |  |
| Right Pericalcarine | Right Lateral Occipital | -0.11 | 0.60 | 0.01746 |  |
| Right Cuneus | Right Lateral Occipital | 0.28 | 0.73 | 0.03379 |  |
| Left Insula | Left Lingual | -0.09 | 0.70 | 0.00380 |  |
| Left Insula | Right Lateral Occipital | -0.02 | 0.64 | 0.04784 |  |
| **Table S12.** Group differences in the strength of inter-regional correlations in cortical thickness between healthy controls (HCs) and youths with childhood-onset conduct disorder (CO-CD) in the Cambridge sample (N=83) *when not* including any covariates in the statistical model. | | | | |  |
|  |  |  |  |  |  |
|  |  | **Metric** | **Metric** |  |  |
| **Region1** | **Region2** | **HCs** | **CO-CD** | **Corr P values** |  |
| Right PreCentral | Right CA Cingulate | -0.30 | 0.48 | 0.00220 | **frontal cortices** |
| Right ParsTriangularis | Right Entorhinal | -0.07 | 0.52 | 0.01984 | **temporal cortices** |
| Left ParsOpercularis | Left Entorhinal | -0.30 | 0.37 | 0.02205 | **parietal cortices** |
| Left ParsOrbitalis | Right Lingual | -0.31 | 0.27 | 0.01764 | **occipital cortices** |
| Left Caudal Middle Frontal | Right Pericalcarine | -0.12 | 0.45 | 0.02058 |  |
| Left Orbital Frontal | Left Pericalcarine | 0.59 | 0.04 | 0.04189 |  |
| Left CA Cingulate | Left Insula | 0.71 | 0.13 | 0.00294 |  |
| Left Inferior Temporal | Right ParsOrbitalis | -0.14 | 0.65 | 0.00000 |  |
| Left Entorhinal | Right Caudal Middle Frontal | -0.19 | 0.48 | 0.00367 |  |
| Left Insula | Right ParsOrbitalis | -0.14 | 0.55 | 0.00882 |  |
| Left Insula | Right PreCentral | -0.02 | 0.55 | 0.01176 |  |
| Left Temporal Pole | Left Inferior Temporal | -0.06 | 0.56 | 0.00147 |  |
| Left Parahippocampal | Right Temporal Pole | -0.11 | 0.45 | 0.02425 |  |
| Right Parahippocampal | Right Inferior Temporal | -0.21 | 0.35 | 0.02572 |  |
| Left Parahippocampal | Right Inferior Temporal | -0.34 | 0.23 | 0.03968 |  |
| Left Entorhinal | Left Inferior Temporal | -0.37 | 0.21 | 0.04629 |  |
| Right Parahippocampal | Right PostCentral | -0.30 | 0.48 | 0.00147 |  |
| Right Banks Superior Temporal | Right SupraMarginal | 0.00 | 0.67 | 0.00220 |  |
| Left Temporal Pole | Left Lateral Occipital | -0.13 | 0.43 | 0.01249 |  |
| Right Fusiform | Right Cuneus | 0.04 | 0.61 | 0.01249 |  |
| Left Superior Parietal | Right Entorhinal | -0.19 | 0.40 | 0.00661 |  |
| Left Lateral Occipital | Right Entorhinal | -0.28 | 0.41 | 0.00147 |  |
| Left PostCentral | Left Superior Parietal | 0.41 | 0.76 | 0.03527 |  |
| Left Inferior Parietal | Right SupraMarginal | 0.39 | 0.80 | 0.04262 |  |
| Left Superior Parietal | Right PostCentral | 0.22 | 0.71 | 0.04850 |  |
| Right Isthmus | Right Lingual | 0.49 | -0.14 | 0.03674 |  |
| Left Lingual | Right Isthmus | 0.50 | -0.19 | 0.00220 |  |
| Left PreCuneus | Right SupraMarginal | 0.31 | 0.81 | 0.01543 |  |
| Left Insula | Left PostCentral | -0.16 | 0.46 | 0.00588 |  |
| Left Insula | Right PostCentral | -0.01 | 0.50 | 0.01911 |  |
| Left Insula | Right Lateral Occipital | -0.02 | 0.63 | 0.00661 |  |

**Table S13.** Group differences in the strength of inter-regional correlations in cortical thickness between youths with childhood-onset conduct disorder (CO-CD) and adolescence-onset conduct disorder (AO-CD) in the Southampton sample (N=69) *when not* including any covariates in the statistical model.

|  |  |  |  |  |  |
| --- | --- | --- | --- | --- | --- |
|  |  | **Metric** | **Metric** |  |  |
| **Region1** | **Region2** | **CO-CD** | **AO-CD** | **Corr P values** |  |
| Left Frontal Pole | Right ParsTriangularis | 0.58 | -0.55 | 0.00000 | **frontal cortices** |
| Right ParsTriangularis | Right Frontal Pole | 0.45 | -0.61 | 0.00000 | **temporal cortices** |
| Right ParsTriangularis | Right PreCentral | 0.78 | -0.07 | 0.00000 | **parietal cortices** |
| Left ParsOrbitalis | Left Rostral Middle Frontal | 0.44 | -0.49 | 0.00015 | **occipital cortices** |
| Left ParsTriangularis | Right Frontal Pole | 0.44 | -0.71 | 0.00015 |  |
| Left ParsOrbitalis | Right Superior Frontal | 0.56 | -0.44 | 0.00170 |  |
| Right ParsTriangularis | Right Superior Frontal | 0.62 | -0.26 | 0.00170 |  |
| Left Frontal Pole | Right ParsOrbitalis | 0.52 | -0.33 | 0.00217 |  |
| Left RA cingulate | Right ParsTriangularis | 0.40 | -0.47 | 0.00263 |  |
| Right ParsOrbitalis | Right Superior Frontal | 0.65 | -0.13 | 0.00341 |  |
| Right ParsOrbitalis | Right RA cingulate | 0.58 | -0.17 | 0.00542 |  |
| Left ParsOpercularis | Right Frontal Pole | 0.39 | -0.43 | 0.00945 |  |
| Right ParsOpercularis | Right PreCentral | 0.51 | -0.20 | 0.01131 |  |
| Left Orbital Frontal | Right ParsTriangularis | 0.54 | -0.34 | 0.01286 |  |
| Left ParsTriangularis | Right Superior Frontal | 0.29 | -0.51 | 0.01348 |  |
| Right CA Cingulate | Right ParsOrbitalis | 0.43 | -0.34 | 0.01519 |  |
| Left Superior Frontal | Right ParsTriangularis | 0.68 | -0.02 | 0.01550 |  |
| Right ParsOrbitalis | Right Frontal Pole | 0.39 | -0.35 | 0.01550 |  |
| Right Orbital Frontal | Right ParsOrbitalis | 0.46 | -0.22 | 0.01565 |  |
| Left ParsTriangularis | Left Frontal Pole | 0.34 | -0.40 | 0.01751 |  |
| Left ParsTriangularis | Left RA cingulate | 0.41 | -0.49 | 0.02015 |  |
| Left ParsOrbitalis | Right Rostral Middle Frontal | 0.27 | -0.46 | 0.02324 |  |
| Left Caudal Middle Frontal | Right ParsTriangularis | 0.59 | -0.04 | 0.02402 |  |
| Left ParsOrbitalis | Right Caudal Middle Frontal | 0.40 | -0.29 | 0.02448 |  |
| Left ParsOrbitalis | Right PreCentral | 0.48 | -0.35 | 0.02510 |  |
| Right Orbital Frontal | Right ParsTriangularis | 0.51 | -0.26 | 0.02665 |  |
| Left ParsTriangularis | Left Rostral Middle Frontal | 0.25 | -0.58 | 0.02882 |  |
| Left ParsOrbitalis | Right Frontal Pole | 0.26 | -0.38 | 0.03223 |  |
| Left Frontal Pole | Right Medial Orbital Frontal | 0.54 | -0.27 | 0.03409 |  |
| Right Caudal Middle Frontal | Right ParsTriangularis | 0.59 | -0.15 | 0.03564 |  |
| Right Caudal Middle Frontal | Right ParsOrbitalis | 0.48 | -0.18 | 0.04076 |  |
| Left Rostral Middle Frontal | Right ParsTriangularis | 0.55 | -0.15 | 0.04571 |  |
| Right ParsTriangularis | Right Superior Temporal | 0.64 | -0.38 | 0.00077 |  |
| Left ParsTriangularis | Right Superior Temporal | 0.50 | -0.54 | 0.00155 |  |
| Right ParsTriangularis | Right Transverse Temporal | 0.21 | -0.63 | 0.00201 |  |
| Left ParsTriangularis | Left Superior Temporal | 0.48 | -0.52 | 0.00356 |  |
| Right ParsTriangularis | Right Temporal Pole | 0.40 | -0.38 | 0.00620 |  |
| Left ParsOrbitalis | Right Banks Superior Temporal | 0.47 | -0.34 | 0.00806 |  |
| Left ParsOrbitalis | Right Parahippocampal | 0.55 | -0.09 | 0.01023 |  |
| Right Frontal Pole | Right Transverse Temporal | 0.18 | 0.69 | 0.01333 |  |
| Right ParsOrbitalis | Right Superior Temporal | 0.43 | -0.26 | 0.01426 |  |
| Left ParsOpercularis | Right Superior Temporal | 0.59 | -0.25 | 0.01519 |  |
| Left ParsOrbitalis | Right Superior Temporal | 0.32 | -0.45 | 0.01829 |  |
| Left ParsTriangularis | Left Transverse Temporal | 0.33 | -0.48 | 0.01875 |  |
| Left Orbital Frontal | Right Transverse Temporal | 0.31 | 0.72 | 0.02355 |  |
| Left ParsTriangularis | Right Temporal Pole | 0.28 | -0.38 | 0.02402 |  |
| Left ParsOpercularis | Right Inferior Temporal | 0.40 | -0.23 | 0.02944 |  |
| Left Frontal Pole | Right Transverse Temporal | 0.19 | 0.67 | 0.03921 |  |
| Left ParsTriangularis | Right Banks Superior Temporal | 0.50 | -0.28 | 0.04184 |  |
| Left ParsOrbitalis | Left Superior Temporal | 0.27 | -0.44 | 0.04277 |  |
| Left ParsOpercularis | Left Superior Temporal | 0.49 | -0.34 | 0.04386 |  |
| Left ParsTriangularis | Left Superior Parietal | 0.51 | -0.66 | 0.00031 |  |
| Left ParsTriangularis | Right Superior Parietal | 0.39 | -0.71 | 0.00046 |  |
| Left ParsOrbitalis | Left Posterior Cingulate | 0.60 | -0.37 | 0.00062 |  |
| Left ParsTriangularis | Right Posterior Cingulate | 0.49 | -0.74 | 0.00155 |  |
| Right ParsTriangularis | Right Posterior Cingulate | 0.61 | -0.33 | 0.00294 |  |
| Left ParsOpercularis | Right Posterior Cingulate | 0.52 | -0.48 | 0.00341 |  |
| Left ParsTriangularis | Right SupraMarginal | 0.52 | -0.43 | 0.00418 |  |
| Left ParsTriangularis | Left SupraMarginal | 0.58 | -0.43 | 0.00496 |  |
| Right ParsTriangularis | Right Superior Parietal | 0.49 | -0.44 | 0.00620 |  |
| Left ParsOrbitalis | Right Posterior Cingulate | 0.53 | -0.35 | 0.00697 |  |

| Left ParsTriangularis | Left Posterior Cingulate | 0.58 | -0.61 | 0.00697 |
| --- | --- | --- | --- | --- |
| Right ParsTriangularis | Right SupraMarginal | 0.58 | -0.26 | 0.00883 |
| Left ParsOpercularis | Left Superior Parietal | 0.38 | -0.56 | 0.00961 |
| Left ParsOrbitalis | Right ParaCentral Lobule | 0.56 | -0.21 | 0.01116 |
| Right ParsOpercularis | Right Posterior Cingulate | 0.64 | -0.16 | 0.02030 |
| Left ParsOpercularis | Left Posterior Cingulate | 0.60 | -0.35 | 0.02092 |
| Right ParsOrbitalis | Right SupraMarginal | 0.59 | -0.10 | 0.02278 |
| Left ParsOpercularis | Right Superior Parietal | 0.27 | -0.49 | 0.03285 |
| Left ParsOrbitalis | Right SupraMarginal | 0.53 | -0.23 | 0.03347 |
| Right Orbital Frontal | Right Superior Parietal | 0.48 | 0.86 | 0.03533 |
| Left ParsOpercularis | Left SupraMarginal | 0.49 | -0.32 | 0.04153 |
| Left ParsTriangularis | Right Lateral Occipital | 0.36 | -0.58 | 0.00713 |
| Right ParsOrbitalis | Right PreCuneus | 0.71 | -0.19 | 0.00961 |
| Left ParsOpercularis | Left Pericalcarine | 0.67 | -0.24 | 0.01240 |
| Left ParsOrbitalis | Right Lateral Occipital | 0.41 | -0.36 | 0.01596 |
| Left ParsOpercularis | Right Pericalcarine | 0.43 | -0.29 | 0.02170 |
| Left ParsTriangularis | Right Pericalcarine | 0.27 | -0.54 | 0.02681 |
| Left ParsTriangularis | Right Insula | 0.37 | -0.68 | 0.00031 |
| Left ParsTriangularis | Left Insula | 0.27 | -0.72 | 0.00124 |
| Right ParsTriangularis | Right Insula | 0.43 | -0.51 | 0.00341 |
| Left Orbital Frontal | Left Insula | 0.37 | 0.89 | 0.00356 |
| Left ParsOrbitalis | Right Insula | 0.23 | -0.55 | 0.00945 |
| Left ParsOpercularis | Right Insula | 0.39 | -0.40 | 0.01085 |
| Left ParsOrbitalis | Left Insula | 0.36 | -0.40 | 0.01178 |
| Left ParsOpercularis | Left Insula | 0.29 | -0.47 | 0.01472 |
| Right Orbital Frontal | Right Insula | 0.44 | 0.85 | 0.02015 |
| Right ParsOrbitalis | Right Insula | 0.34 | -0.37 | 0.02510 |
| Left Transverse Temporal | Right ParsTriangularis | 0.53 | -0.60 | 0.00000 |
| Right Parahippocampal | Right ParsOrbitalis | 0.58 | -0.38 | 0.00093 |
| Right Parahippocampal | Right ParsTriangularis | 0.61 | -0.20 | 0.00124 |
| Left Temporal Pole | Right ParsTriangularis | 0.63 | -0.26 | 0.00170 |
| Right Banks Superior Temporal | Right ParsOrbitalis | 0.52 | -0.45 | 0.00217 |
| Left Banks Superior Temporal | Right ParsTriangularis | 0.66 | -0.23 | 0.00279 |
| Left Superior Temporal | Right ParsTriangularis | 0.48 | -0.42 | 0.00294 |
| Left Banks Superior Temporal | Left ParsTriangularis | 0.45 | -0.41 | 0.00666 |
| Left Middle Temporal | Right ParsOpercularis | 0.41 | -0.27 | 0.00697 |
| Right Parahippocampal | Right ParsOpercularis | 0.57 | -0.21 | 0.00775 |
| Left Entorhinal | Right ParsTriangularis | 0.58 | -0.24 | 0.00852 |
| Left Parahippocampal | Right ParsOpercularis | 0.68 | 0.01 | 0.00992 |
| Right Banks Superior Temporal | Right ParsTriangularis | 0.58 | -0.15 | 0.01038 |
| Right Entorhinal | Right ParsOrbitalis | 0.57 | -0.19 | 0.01178 |
| Left Banks Superior Temporal | Left ParsOrbitalis | 0.28 | -0.48 | 0.01317 |
| Left Temporal Pole | Right PreCentral | 0.64 | 0.01 | 0.01519 |
| Right Entorhinal | Right ParsTriangularis | 0.60 | -0.23 | 0.02588 |
| Right Inferior Temporal | Right ParsTriangularis | 0.41 | -0.44 | 0.02789 |
| Left Inferior Temporal | Left Frontal Pole | 0.60 | -0.20 | 0.03177 |
| Left Transverse Temporal | Right Frontal Pole | 0.30 | 0.78 | 0.04680 |
| Left Isthmus | Right Medial Orbital Frontal | 0.48 | -0.55 | 0.00093 |
| Left Posterior Cingulate | Right ParsTriangularis | 0.63 | -0.32 | 0.00325 |
| Right Inferior Parietal | Right PreCentral | 0.63 | -0.06 | 0.00449 |
| Right ParaCentral Lobule | Right ParsOrbitalis | 0.57 | -0.22 | 0.00635 |
| Left Posterior Cingulate | Right ParsOrbitalis | 0.67 | -0.12 | 0.00682 |
| Left Superior Parietal | Right ParsTriangularis | 0.56 | -0.36 | 0.00821 |
| Left PostCentral | Right ParsTriangularis | 0.68 | 0.09 | 0.01658 |
| Left SupraMarginal | Right ParsTriangularis | 0.52 | -0.34 | 0.01705 |
| Left Isthmus | Left Medial Orbital Frontal | 0.40 | -0.48 | 0.01953 |
| Left Isthmus | Right ParsOpercularis | 0.33 | -0.44 | 0.01953 |
| Left PostCentral | Left RA cingulate | 0.66 | -0.04 | 0.02030 |
| Right Inferior Parietal | Right Frontal Pole | 0.53 | -0.36 | 0.02154 |
| Right Cuneus | Right PreCentral | 0.61 | -0.23 | 0.00170 |
| Left Lingual | Right ParsOpercularis | 0.41 | -0.56 | 0.00852 |
| Right Lateral Occipital | Right ParsTriangularis | 0.55 | -0.31 | 0.01209 |
| Left Cuneus | Right ParsOrbitalis | 0.52 | -0.17 | 0.01999 |
| Left Lateral Occipital | Right Caudal Middle Frontal | 0.66 | 0.92 | 0.02572 |
| Left Lateral Occipital | Right ParsOrbitalis | 0.48 | -0.30 | 0.03099 |
| Left Cuneus | Right Orbital Frontal | 0.58 | 0.07 | 0.03487 |

| Right Lateral Occipital | Right ParsOrbitalis | 0.51 | -0.18 | 0.03673 |  |
| --- | --- | --- | --- | --- | --- |
| Left Cuneus | Right Medial Orbital Frontal | 0.57 | -0.34 | 0.03967 |  |
| Left Lateral Occipital | Left ParsOrbitalis | 0.44 | -0.24 | 0.04138 |  |
| Left Cuneus | Right ParsTriangularis | 0.44 | -0.27 | 0.04695 |  |
| Left Insula | Right ParsTriangularis | 0.55 | -0.47 | 0.00077 |  |
| Left Insula | Right ParsOrbitalis | 0.47 | -0.21 | 0.01224 |  |
| Left Transverse Temporal | Right Transverse Temporal | 0.50 | 0.81 | 0.02015 |  |
| Left Parahippocampal | Right Entorhinal | 0.58 | -0.10 | 0.03363 |  |
| Left Entorhinal | Right Parahippocampal | 0.67 | 0.11 | 0.04231 |  |
| Left Transverse Temporal | Right Inferior Parietal | 0.54 | -0.31 | 0.00573 |  |
| Left Temporal Pole | Right Lingual | 0.34 | -0.26 | 0.04649 |  |
| Left Isthmus | Left Parahippocampal | 0.48 | -0.27 | 0.04510 |  |
| Left Insula | Right Transverse Temporal | 0.03 | 0.68 | 0.00108 |  |
| Left Insula | Right Temporal Pole | -0.04 | 0.59 | 0.00666 |  |
| Right Superior Parietal | Right SupraMarginal | 0.60 | 0.92 | 0.01441 |  |
| Right Inferior Parietal | Right Posterior Cingulate | 0.64 | -0.12 | 0.03750 |  |
| Left Posterior Cingulate | Right Inferior Parietal | 0.59 | -0.08 | 0.04277 |  |
| Right Inferior Parietal | Right Insula | 0.51 | -0.36 | 0.02402 |  |
| Left Insula | Right Superior Parietal | 0.41 | 0.94 | 0.00821 |  |
| Left Pericalcarine | Right Pericalcarine | 0.35 | 0.81 | 0.01162 |  |
| **Table S14.** Group differences in the strength of inter-regional correlations in cortical thickness between healthy controls (HCs) and youths with adolescence-onset conduct disorder (AO-CD) in the Southampton sample (N=69) *when not* including any covariates in the statistical model. | | | | |  |
|  |  |  |  |  |  |
|  |  | **Metric** | **Metric** |  |  |
| **Region1** | **Region2** | **HCs** | **AO-CD** | **Corr P values** |  |
| Left ParsTriangularis | Right Frontal Pole | -0.01 | -0.71 | 0.00479 | **frontal cortices** |
| Left Caudal Middle Frontal | Right Caudal Middle Frontal | 0.59 | 0.90 | 0.00995 | **temporal cortices** |
| Left ParsTriangularis | Right Orbital Frontal | 0.27 | -0.55 | 0.01129 | **parietal cortices** |
| Left ParsOrbitalis | Right Rostral Middle Frontal | 0.27 | -0.46 | 0.01302 | **occipital cortices** |
| Left ParsTriangularis | Right PreCentral | 0.33 | -0.32 | 0.02489 |  |
| Left Caudal Middle Frontal | Left Superior Frontal | 0.41 | 0.81 | 0.02508 |  |
| Left Orbital Frontal | Right Superior Frontal | 0.39 | 0.82 | 0.02642 |  |
| Left Orbital Frontal | Left RA cingulate | 0.10 | 0.59 | 0.04077 |  |
| Left ParsTriangularis | Right Superior Frontal | 0.06 | -0.51 | 0.04211 |  |
| Left Orbital Frontal | Left Superior Frontal | 0.44 | 0.82 | 0.04250 |  |
| Left ParsTriangularis | Right Superior Temporal | 0.33 | -0.54 | 0.00077 |  |
| Left ParsTriangularis | Right Transverse Temporal | 0.38 | -0.51 | 0.00230 |  |
| Left ParsTriangularis | Left Superior Temporal | 0.16 | -0.52 | 0.01129 |  |
| Left Caudal Middle Frontal | Right Banks Superior Temporal | 0.47 | 0.86 | 0.01857 |  |
| Right Frontal Pole | Right Transverse Temporal | 0.26 | 0.69 | 0.01991 |  |
| Right ParsTriangularis | Right Transverse Temporal | 0.01 | -0.63 | 0.03656 |  |
| Right Superior Frontal | Right Superior Temporal | 0.58 | 0.87 | 0.04097 |  |
| Left Orbital Frontal | Left Transverse Temporal | 0.25 | 0.67 | 0.04211 |  |
| Left Caudal Middle Frontal | Right Parahippocampal | 0.03 | 0.57 | 0.04231 |  |
| Left CA Cingulate | Right Parahippocampal | 0.06 | 0.62 | 0.04288 |  |
| Left Orbital Frontal | Right Superior Parietal | 0.36 | 0.91 | 0.00000 |  |
| Left Orbital Frontal | Right SupraMarginal | 0.24 | 0.91 | 0.00000 |  |
| Left ParsTriangularis | Right Posterior Cingulate | 0.51 | -0.74 | 0.00000 |  |
| Left ParsTriangularis | Left Posterior Cingulate | 0.34 | -0.61 | 0.00019 |  |
| Right Orbital Frontal | Right SupraMarginal | 0.34 | 0.88 | 0.00038 |  |
| Left Orbital Frontal | Right Posterior Cingulate | 0.25 | 0.82 | 0.00077 |  |
| Left Orbital Frontal | Left SupraMarginal | 0.34 | 0.89 | 0.00096 |  |
| Left ParsTriangularis | Right Superior Parietal | 0.10 | -0.71 | 0.00172 |  |
| Left Medial Orbital Frontal | Right SupraMarginal | -0.03 | 0.69 | 0.00459 |  |
| Left ParsOpercularis | Right Posterior Cingulate | 0.47 | -0.48 | 0.00632 |  |
| Left ParsTriangularis | Right SupraMarginal | 0.29 | -0.43 | 0.00632 |  |
| Left Medial Orbital Frontal | Left SupraMarginal | 0.14 | 0.77 | 0.01436 |  |
| Left ParsOpercularis | Left Superior Parietal | 0.21 | -0.56 | 0.01455 |  |
| Left Orbital Frontal | Left Posterior Cingulate | 0.48 | 0.86 | 0.01551 |  |
| Left ParsTriangularis | Left Superior Parietal | 0.06 | -0.66 | 0.01570 |  |
| Left RA cingulate | Left Superior Parietal | 0.29 | 0.76 | 0.01665 |  |
| Left RA cingulate | Right Superior Parietal | 0.30 | 0.69 | 0.02718 |  |
| Right Orbital Frontal | Right Superior Parietal | 0.49 | 0.86 | 0.03580 |  |
| Left ParsOpercularis | Left Posterior Cingulate | 0.33 | -0.35 | 0.03867 |  |

| Left Orbital Frontal | Left Superior Parietal | 0.55 | 0.85 | 0.04479 |
| --- | --- | --- | --- | --- |
| Left Caudal Middle Frontal | Left Lateral Occipital | 0.19 | 0.89 | 0.00000 |
| Right Caudal Middle Frontal | Right Lateral Occipital | 0.47 | 0.87 | 0.00115 |
| Left ParsTriangularis | Right Pericalcarine | 0.32 | -0.54 | 0.00364 |
| Left PreCentral | Right Cuneus | 0.43 | -0.35 | 0.00479 |
| Left CA Cingulate | Left Lateral Occipital | 0.11 | 0.60 | 0.03273 |
| Left Orbital Frontal | Right Lateral Occipital | 0.37 | 0.78 | 0.03369 |
| Left ParsOpercularis | Left Pericalcarine | 0.46 | -0.24 | 0.04901 |
| Left ParsTriangularis | Left Insula | 0.17 | -0.72 | 0.00019 |
| Left ParsTriangularis | Right Insula | 0.19 | -0.68 | 0.00211 |
| Right Frontal Pole | Right Insula | 0.42 | 0.81 | 0.00536 |
| Left ParsOrbitalis | Right Insula | 0.06 | -0.55 | 0.00823 |
| Left Orbital Frontal | Left Insula | 0.55 | 0.89 | 0.00938 |
| Left RA cingulate | Right Insula | 0.15 | 0.65 | 0.01493 |
| Left ParsOpercularis | Left Insula | 0.26 | -0.47 | 0.02163 |
| Left Banks Superior Temporal | Left Orbital Frontal | 0.37 | 0.85 | 0.00172 |
| Left Inferior Temporal | Left Frontal Pole | 0.48 | -0.20 | 0.01015 |
| Left Banks Superior Temporal | Left ParsTriangularis | 0.22 | -0.41 | 0.04097 |
| Left Isthmus | Right Medial Orbital Frontal | 0.32 | -0.55 | 0.00096 |
| Left PostCentral | Left Superior Frontal | 0.26 | 0.72 | 0.00555 |
| Left Inferior Parietal | Right Rostral Middle Frontal | 0.37 | -0.19 | 0.00938 |
| Right Superior Parietal | Right Frontal Pole | 0.32 | 0.70 | 0.01761 |
| Right Inferior Parietal | Right Frontal Pole | 0.42 | -0.36 | 0.02852 |
| Left Isthmus | Right ParsOpercularis | 0.21 | -0.44 | 0.02967 |
| Right Posterior Cingulate | Right Superior Frontal | 0.41 | 0.80 | 0.03254 |
| Left Isthmus | Left Medial Orbital Frontal | 0.08 | -0.48 | 0.03675 |
| Left Isthmus | Left ParsOpercularis | 0.14 | -0.39 | 0.03867 |
| Left Lateral Occipital | Right Caudal Middle Frontal | 0.02 | 0.92 | 0.00000 |
| Left Lateral Occipital | Right CA Cingulate | -0.05 | 0.68 | 0.00191 |
| Left Pericalcarine | Left PreCentral | 0.37 | 0.80 | 0.00479 |
| Left Lingual | Left ParsOpercularis | 0.34 | -0.44 | 0.00689 |
| Right Cuneus | Right PreCentral | 0.60 | -0.23 | 0.01187 |
| Right Lateral Occipital | Right Frontal Pole | 0.31 | 0.73 | 0.02297 |
| Left Lateral Occipital | Right Superior Frontal | 0.14 | 0.66 | 0.02412 |
| Left Lateral Occipital | Right Frontal Pole | 0.25 | 0.66 | 0.03561 |
| Right PreCuneus | Right Frontal Pole | 0.01 | 0.53 | 0.04020 |
| Left Pericalcarine | Right PreCentral | 0.43 | 0.85 | 0.04479 |
| Left PreCuneus | Right Superior Frontal | 0.07 | 0.60 | 0.04786 |
| Left Insula | Right Frontal Pole | 0.43 | 0.80 | 0.00383 |
| Left Middle Temporal | Left Parahippocampal | 0.28 | 0.74 | 0.01914 |
| Left Banks Superior Temporal | Left Lateral Occipital | 0.10 | 0.66 | 0.00957 |
| Left Entorhinal | Left PreCuneus | -0.11 | 0.50 | 0.02316 |
| Left Lateral Occipital | Right Entorhinal | -0.09 | 0.69 | 0.00019 |
| Left Lateral Occipital | Left Superior Temporal | 0.12 | 0.74 | 0.00421 |
| Left Lateral Occipital | Right Banks Superior Temporal | 0.03 | 0.72 | 0.00555 |
| Right Lateral Occipital | Right Temporal Pole | 0.00 | 0.54 | 0.01340 |
| Left Lateral Occipital | Right Superior Temporal | 0.08 | 0.58 | 0.03599 |
| Right Superior Parietal | Right SupraMarginal | 0.53 | 0.92 | 0.00077 |
| Left Posterior Cingulate | Right Superior Parietal | 0.31 | 0.85 | 0.00096 |
| Right Posterior Cingulate | Right Superior Parietal | 0.24 | 0.84 | 0.00211 |
| Left SupraMarginal | Right Posterior Cingulate | 0.28 | 0.80 | 0.01780 |
| Left Posterior Cingulate | Left SupraMarginal | 0.42 | 0.82 | 0.01838 |
| Left Posterior Cingulate | Left Superior Parietal | 0.42 | 0.78 | 0.02527 |
| Left Superior Parietal | Right Posterior Cingulate | 0.26 | 0.75 | 0.03120 |
| Left Posterior Cingulate | Right Posterior Cingulate | 0.60 | 0.88 | 0.03829 |
| Left Superior Parietal | Right SupraMarginal | 0.47 | 0.83 | 0.04384 |
| Left Posterior Cingulate | Left PreCuneus | 0.02 | 0.72 | 0.00057 |
| Right Isthmus | Right Lingual | -0.21 | 0.69 | 0.00096 |
| Left Posterior Cingulate | Right Lateral Occipital | 0.25 | 0.75 | 0.00995 |
| Left Posterior Cingulate | Right PreCuneus | 0.07 | 0.70 | 0.01627 |
| Left ParaCentral Lobule | Left PreCuneus | -0.05 | 0.54 | 0.01799 |
| Right ParaCentral Lobule | Right PreCuneus | 0.21 | 0.72 | 0.02010 |
| Left SupraMarginal | Right Pericalcarine | 0.39 | 0.77 | 0.03848 |
| Left Superior Parietal | Right Lateral Occipital | 0.25 | 0.71 | 0.04537 |
| Right Superior Parietal | Right Insula | 0.53 | 0.88 | 0.01819 |
| Left Lateral Occipital | Left Posterior Cingulate | 0.03 | 0.66 | 0.00230 |

| Left PreCuneus | Right ParaCentral Lobule | -0.06 | 0.58 | 0.00651 |
| --- | --- | --- | --- | --- |
| Left Lateral Occipital | Right SupraMarginal | -0.04 | 0.61 | 0.00957 |
| Left Lateral Occipital | Right ParaCentral Lobule | -0.14 | 0.52 | 0.01704 |
| Left Lateral Occipital | Right Superior Parietal | 0.14 | 0.64 | 0.03331 |
| Left Insula | Right Superior Parietal | 0.68 | 0.94 | 0.00536 |
| Left Insula | Right SupraMarginal | 0.47 | 0.84 | 0.04403 |
| Left PreCuneus | Right PreCuneus | 0.39 | 0.87 | 0.00172 |
| Left Lateral Occipital | Right Lateral Occipital | 0.52 | 0.89 | 0.00268 |
| Left Pericalcarine | Left PreCuneus | 0.06 | 0.58 | 0.03695 |
| Left Pericalcarine | Right Pericalcarine | 0.49 | 0.81 | 0.04097 |
| Left Lateral Occipital | Right Insula | 0.07 | 0.67 | 0.00651 |
| Right Lateral Occipital | Right Insula | 0.22 | 0.79 | 0.01072 |
| Left Lateral Occipital | Left Insula | 0.22 | 0.71 | 0.02450 |
| Left Insula | Right Lateral Occipital | 0.30 | 0.81 | 0.00268 |

**Table S15.** Group differences in the strength of inter-regional correlations in cortical thickness between healthy controls (HCs) and youths with childhood-onset conduct disorder (CO-CD) in the Southampton sample (N=69) *when not* including any covariates in the statistical model.

|  |  |  |  |  |  |
| --- | --- | --- | --- | --- | --- |
|  |  | **Metric** | **Metric** |  |  |
| **Region1** | **Region2** | **HCs** | **CO-CD** | **Corr P values** |  |
| Left Superior Frontal | Right ParsTriangularis | -0.16 | 0.61 | 0.00000 | **frontal cortices** |
| Right ParsOrbitalis | Right RA cingulate | -0.19 | 0.58 | 0.00000 | **temporal cortices** |
| Left Superior Frontal | Right ParsOrbitalis | -0.12 | 0.63 | 0.00013 | **parietal cortices** |
| Left Frontal Pole | Right ParsTriangularis | -0.16 | 0.59 | 0.00065 | **occipital cortices** |
| Right Caudal Middle Frontal | Right ParsTriangularis | -0.06 | 0.60 | 0.00065 |  |
| Left Superior Frontal | Right Superior Frontal | 0.51 | 0.90 | 0.00117 |  |
| Left RA cingulate | Right ParsOrbitalis | -0.21 | 0.43 | 0.00169 |  |
| Right ParsTriangularis | Right Frontal Pole | -0.22 | 0.38 | 0.00221 |  |
| Right ParsOrbitalis | Right Superior Frontal | 0.03 | 0.65 | 0.00273 |  |
| Right ParsTriangularis | Right Superior Frontal | -0.04 | 0.55 | 0.00312 |  |
| Left Caudal Middle Frontal | Right ParsTriangularis | 0.08 | 0.63 | 0.00364 |  |
| Left Caudal Middle Frontal | Right ParsOrbitalis | -0.22 | 0.50 | 0.00377 |  |
| Right Caudal Middle Frontal | Right ParsOrbitalis | 0.03 | 0.65 | 0.00495 |  |
| Right ParsTriangularis | Right PreCentral | 0.28 | 0.73 | 0.00703 |  |
| Left ParsOrbitalis | Left Superior Frontal | -0.06 | 0.52 | 0.00859 |  |
| Left RA cingulate | Right RA cingulate | 0.51 | 0.86 | 0.00885 |  |
| Left ParsOrbitalis | Right Orbital Frontal | -0.12 | 0.47 | 0.01159 |  |
| Left Rostral Middle Frontal | Right ParsTriangularis | -0.16 | 0.50 | 0.01874 |  |
| Left ParsOrbitalis | Right RA cingulate | -0.07 | 0.47 | 0.02356 |  |
| Left PreCentral | Right RA cingulate | 0.11 | 0.60 | 0.02356 |  |
| Left ParsTriangularis | Right Frontal Pole | -0.05 | 0.41 | 0.02369 |  |
| Left ParsOrbitalis | Right Caudal Middle Frontal | 0.08 | 0.58 | 0.02382 |  |
| Right Medial Orbital Frontal | Right ParsTriangularis | 0.12 | 0.57 | 0.02942 |  |
| Right CA Cingulate | Right Rostral Middle Frontal | 0.26 | 0.68 | 0.03384 |  |
| Left Orbital Frontal | Right ParsTriangularis | -0.02 | 0.49 | 0.03476 |  |
| Right Orbital Frontal | Right ParsOrbitalis | 0.10 | 0.60 | 0.04192 |  |
| Right CA Cingulate | Right ParsOrbitalis | 0.03 | 0.52 | 0.04465 |  |
| Left ParsOrbitalis | Right Superior Frontal | -0.04 | 0.50 | 0.04816 |  |
| Left Medial Orbital Frontal | Right Transverse Temporal | 0.29 | -0.28 | 0.00325 |  |
| Right ParsTriangularis | Right Superior Temporal | -0.03 | 0.62 | 0.00325 |  |
| Left CA Cingulate | Right Inferior Temporal | 0.71 | 0.10 | 0.01093 |  |
| Right CA Cingulate | Right Entorhinal | 0.84 | 0.49 | 0.02226 |  |
| Left RA cingulate | Right Superior Temporal | 0.45 | 0.75 | 0.03983 |  |
| Right ParsOpercularis | Right Superior Temporal | 0.14 | 0.65 | 0.04061 |  |
| Left Medial Orbital Frontal | Left Middle Temporal | 0.16 | 0.63 | 0.04152 |  |
| Right ParsTriangularis | Right Superior Parietal | -0.25 | 0.55 | 0.00052 |  |
| Left Medial Orbital Frontal | Right Isthmus | 0.42 | -0.34 | 0.00495 |  |
| Right ParsOrbitalis | Right PostCentral | 0.08 | 0.62 | 0.00807 |  |
| Left RA cingulate | Right Posterior Cingulate | 0.40 | 0.78 | 0.02005 |  |
| Right ParsTriangularis | Right PostCentral | 0.21 | 0.60 | 0.02616 |  |
| Left Superior Frontal | Right Posterior Cingulate | 0.50 | 0.83 | 0.03450 |  |
| Left CA Cingulate | Left Posterior Cingulate | 0.67 | 0.21 | 0.04738 |  |
| Right ParsTriangularis | Right SupraMarginal | 0.10 | 0.58 | 0.04999 |  |
| Left Caudal Middle Frontal | Right PreCuneus | -0.04 | 0.67 | 0.00104 |  |
| Right ParsOrbitalis | Right PreCuneus | 0.06 | 0.75 | 0.00182 |  |

| Left CA Cingulate | Left PreCuneus | -0.20 | 0.49 | 0.00508 |
| --- | --- | --- | --- | --- |
| Right Orbital Frontal | Right Lingual | 0.29 | -0.31 | 0.01848 |
| Right Caudal Middle Frontal | Right PreCuneus | 0.32 | 0.76 | 0.03293 |
| Left Orbital Frontal | Right Lateral Occipital | 0.38 | 0.77 | 0.04634 |
| Right ParsTriangularis | Right Insula | -0.31 | 0.36 | 0.00469 |
| Left Entorhinal | Right ParsOrbitalis | -0.15 | 0.72 | 0.00000 |
| Left Banks Superior Temporal | Right ParsTriangularis | -0.02 | 0.62 | 0.00078 |
| Left Entorhinal | Right ParsOpercularis | -0.02 | 0.65 | 0.00260 |
| Left Entorhinal | Left ParsOrbitalis | -0.14 | 0.63 | 0.00325 |
| Left Transverse Temporal | Right ParsTriangularis | -0.16 | 0.50 | 0.00391 |
| Left Entorhinal | Left Superior Frontal | 0.31 | 0.70 | 0.00664 |
| Left Banks Superior Temporal | Right ParsOpercularis | -0.09 | 0.54 | 0.00677 |
| Left Superior Temporal | Right ParsTriangularis | -0.07 | 0.49 | 0.00755 |
| Left Temporal Pole | Right ParsTriangularis | -0.12 | 0.54 | 0.00768 |
| Right Parahippocampal | Right ParsOrbitalis | -0.11 | 0.51 | 0.00781 |
| Right Parahippocampal | Right ParsTriangularis | 0.02 | 0.57 | 0.00950 |
| Right Entorhinal | Right Orbital Frontal | 0.37 | 0.85 | 0.01705 |
| Left Fusiform | Right CA Cingulate | 0.49 | -0.10 | 0.01992 |
| Right Banks Superior Temporal | Right ParsTriangularis | 0.16 | 0.58 | 0.02577 |
| Right Entorhinal | Right ParsOrbitalis | 0.01 | 0.54 | 0.02994 |
| Left Parahippocampal | Right ParsOpercularis | 0.17 | 0.61 | 0.03163 |
| Left Parahippocampal | Right Medial Orbital Frontal | 0.14 | 0.61 | 0.04803 |
| Left PostCentral | Right Frontal Pole | -0.10 | 0.57 | 0.00065 |
| Right Posterior Cingulate | Right RA cingulate | 0.29 | 0.83 | 0.00273 |
| Left Superior Parietal | Right ParsTriangularis | -0.22 | 0.59 | 0.00364 |
| Right Posterior Cingulate | Right Superior Frontal | 0.40 | 0.79 | 0.00391 |
| Left Inferior Parietal | Right ParsTriangularis | 0.10 | 0.63 | 0.00534 |
| Left Inferior Parietal | Left ParsTriangularis | 0.21 | 0.75 | 0.00742 |
| Left PostCentral | Right ParsOrbitalis | -0.12 | 0.58 | 0.01041 |
| Left SupraMarginal | Right ParsOpercularis | -0.17 | 0.41 | 0.01041 |
| Left Posterior Cingulate | Right ParsOrbitalis | -0.04 | 0.61 | 0.02083 |
| Left Superior Parietal | Right PreCentral | 0.25 | 0.67 | 0.02109 |
| Left PostCentral | Left Superior Frontal | 0.36 | 0.76 | 0.02161 |
| Left Posterior Cingulate | Right ParsTriangularis | 0.12 | 0.59 | 0.02708 |
| Left Posterior Cingulate | Right RA cingulate | 0.40 | 0.77 | 0.02838 |
| Left PostCentral | Right ParsTriangularis | 0.26 | 0.71 | 0.02877 |
| Left Lateral Occipital | Right ParsOrbitalis | -0.33 | 0.57 | 0.00039 |
| Left Cuneus | Right ParsOrbitalis | -0.16 | 0.62 | 0.00052 |
| Left Lateral Occipital | Left Orbital Frontal | 0.31 | 0.83 | 0.00078 |
| Right Lateral Occipital | Right ParsTriangularis | -0.18 | 0.52 | 0.00130 |
| Right Cuneus | Right ParsTriangularis | -0.03 | 0.58 | 0.00143 |
| Left PreCuneus | Right ParsTriangularis | -0.19 | 0.50 | 0.00169 |
| Right PreCuneus | Right Superior Frontal | 0.32 | 0.79 | 0.00182 |
| Left PreCuneus | Right PreCentral | 0.05 | 0.68 | 0.00299 |
| Left Lateral Occipital | Left ParsOrbitalis | -0.18 | 0.53 | 0.00377 |
| Right Lateral Occipital | Right ParsOrbitalis | 0.15 | 0.64 | 0.00495 |
| Right Cuneus | Right ParsOrbitalis | -0.20 | 0.50 | 0.00521 |
| Left Lateral Occipital | Right Orbital Frontal | 0.17 | 0.73 | 0.00638 |
| Left Cuneus | Left ParsOrbitalis | -0.04 | 0.59 | 0.00677 |
| Left Lateral Occipital | Right ParsOpercularis | -0.10 | 0.48 | 0.00885 |
| Left PreCuneus | Right Superior Frontal | -0.05 | 0.50 | 0.00898 |
| Right Lateral Occipital | Right Orbital Frontal | 0.31 | 0.81 | 0.01237 |
| Left Pericalcarine | Right RA cingulate | 0.15 | 0.64 | 0.01627 |
| Left Cuneus | Right ParsOpercularis | -0.04 | 0.44 | 0.01666 |
| Left Lateral Occipital | Right RA cingulate | 0.05 | 0.55 | 0.01770 |
| Left Cuneus | Right RA cingulate | -0.07 | 0.48 | 0.02317 |
| Right Lateral Occipital | Right RA cingulate | 0.27 | 0.67 | 0.02317 |
| Left Lateral Occipital | Right ParsTriangularis | -0.15 | 0.41 | 0.02682 |
| Left Lateral Occipital | Right Superior Frontal | 0.07 | 0.60 | 0.03163 |
| Left Lateral Occipital | Right Caudal Middle Frontal | 0.18 | 0.61 | 0.03306 |
| Left Lingual | Right Orbital Frontal | 0.26 | -0.22 | 0.03358 |
| Left Lateral Occipital | Left Medial Orbital Frontal | -0.26 | 0.33 | 0.03905 |
| Left Insula | Right ParsTriangularis | -0.26 | 0.39 | 0.00391 |
| Left Entorhinal | Right Parahippocampal | -0.09 | 0.70 | 0.00026 |
| Left Middle Temporal | Right Inferior Temporal | 0.34 | -0.48 | 0.00195 |
| Right Inferior Temporal | Right Temporal Pole | -0.19 | 0.40 | 0.01575 |

| Left Middle Temporal | Left Temporal Pole | 0.27 | -0.26 | 0.01731 |
| --- | --- | --- | --- | --- |
| Left Inferior Temporal | Right Inferior Temporal | 0.64 | 0.15 | 0.03306 |
| Left Entorhinal | Right Superior Temporal | 0.30 | 0.67 | 0.03918 |
| Left Entorhinal | Left Parahippocampal | 0.14 | 0.63 | 0.04608 |
| Left Entorhinal | Right SupraMarginal | 0.10 | 0.68 | 0.00469 |
| Right Parahippocampal | Right ParaCentral Lobule | 0.20 | 0.74 | 0.00690 |
| Left Entorhinal | Left Superior Parietal | -0.02 | 0.49 | 0.00742 |
| Left Entorhinal | Right Superior Parietal | 0.02 | 0.42 | 0.02252 |
| Left Fusiform | Left Isthmus | 0.43 | -0.08 | 0.02864 |
| Left Entorhinal | Left PreCuneus | -0.25 | 0.62 | 0.00013 |
| Left Entorhinal | Right PreCuneus | -0.01 | 0.63 | 0.00065 |
| Right Inferior Temporal | Right Lingual | 0.70 | -0.05 | 0.00117 |
| Left Banks Superior Temporal | Left PreCuneus | 0.08 | 0.56 | 0.00872 |
| Left Entorhinal | Right Lateral Occipital | 0.36 | 0.73 | 0.01172 |
| Left Superior Temporal | Right Lateral Occipital | 0.43 | 0.78 | 0.01302 |
| Left Banks Superior Temporal | Right Lateral Occipital | 0.34 | 0.76 | 0.02616 |
| Left Superior Temporal | Right PreCuneus | 0.17 | 0.67 | 0.03176 |
| Left Temporal Pole | Right Lingual | -0.29 | 0.18 | 0.03345 |
| Right Entorhinal | Right Lateral Occipital | 0.35 | 0.73 | 0.03827 |
| Right Fusiform | Right Lingual | 0.39 | -0.12 | 0.04283 |
| Left ParaCentral Lobule | Right Transverse Temporal | 0.47 | -0.02 | 0.01809 |
| Right Posterior Cingulate | Right Superior Temporal | 0.49 | 0.84 | 0.02057 |
| Right ParaCentral Lobule | Right Transverse Temporal | 0.47 | -0.01 | 0.02682 |
| Right Isthmus | Right Transverse Temporal | -0.02 | 0.52 | 0.03046 |
| Right Inferior Parietal | Right Inferior Temporal | 0.67 | 0.11 | 0.03306 |
| Left Lateral Occipital | Left Superior Temporal | -0.02 | 0.68 | 0.00039 |
| Left Lateral Occipital | Right Banks Superior Temporal | 0.07 | 0.72 | 0.00495 |
| Left Lateral Occipital | Right Entorhinal | -0.01 | 0.55 | 0.00833 |
| Left Cuneus | Left Entorhinal | 0.34 | 0.73 | 0.00898 |
| Left PreCuneus | Right Entorhinal | -0.25 | 0.37 | 0.01172 |
| Left Lateral Occipital | Left Middle Temporal | -0.17 | 0.33 | 0.02070 |
| Left Lingual | Right Inferior Temporal | 0.34 | -0.25 | 0.02603 |
| Left Lingual | Right Transverse Temporal | 0.44 | -0.21 | 0.02642 |
| Left Lateral Occipital | Right Temporal Pole | -0.24 | 0.31 | 0.04517 |
| Left PreCuneus | Right Banks Superior Temporal | 0.16 | 0.56 | 0.04777 |
| Right Posterior Cingulate | Right Superior Parietal | 0.06 | 0.59 | 0.01432 |
| Left SupraMarginal | Right Posterior Cingulate | 0.22 | 0.69 | 0.02083 |
| Left Posterior Cingulate | Left SupraMarginal | 0.42 | 0.82 | 0.02148 |
| Left Inferior Parietal | Right Isthmus | 0.45 | -0.10 | 0.02304 |
| Left Posterior Cingulate | Right PreCuneus | 0.08 | 0.63 | 0.00456 |
| Left Inferior Parietal | Left PreCuneus | -0.09 | 0.57 | 0.00690 |
| Left SupraMarginal | Right Lateral Occipital | 0.36 | 0.74 | 0.00820 |
| Left Posterior Cingulate | Right Lateral Occipital | 0.43 | 0.77 | 0.01744 |
| Right Posterior Cingulate | Right PreCuneus | 0.22 | 0.63 | 0.02877 |
| Left Lateral Occipital | Right ParaCentral Lobule | 0.12 | 0.69 | 0.00417 |
| Left Lateral Occipital | Left Posterior Cingulate | 0.19 | 0.70 | 0.00521 |
| Left Lateral Occipital | Right Posterior Cingulate | 0.00 | 0.56 | 0.00612 |
| Left Cuneus | Right ParaCentral Lobule | 0.25 | 0.76 | 0.00638 |
| Right Cuneus | Right Inferior Parietal | 0.42 | 0.81 | 0.01445 |
| Left Lateral Occipital | Right SupraMarginal | 0.09 | 0.62 | 0.01497 |
| Right Cuneus | Right Posterior Cingulate | 0.25 | 0.67 | 0.02278 |
| Left Lateral Occipital | Left Superior Parietal | 0.04 | 0.54 | 0.02773 |
| Left Lateral Occipital | Left SupraMarginal | 0.21 | 0.69 | 0.02994 |
| Right Cuneus | Right Superior Parietal | 0.45 | 0.78 | 0.04335 |
| Left Pericalcarine | Left PreCuneus | -0.05 | 0.60 | 0.00026 |
| Left Lateral Occipital | Right Lateral Occipital | 0.32 | 0.82 | 0.00078 |
| Left PreCuneus | Right Cuneus | -0.07 | 0.53 | 0.00247 |
| Left Cuneus | Left PreCuneus | -0.17 | 0.51 | 0.00351 |
| Left Lateral Occipital | Left PreCuneus | 0.10 | 0.66 | 0.00638 |
| Right Lateral Occipital | Right PreCuneus | 0.31 | 0.77 | 0.00859 |
| Left Lateral Occipital | Right PreCuneus | 0.06 | 0.65 | 0.01692 |
| Left PreCuneus | Right Lateral Occipital | 0.04 | 0.54 | 0.01966 |
| Left Cuneus | Right PreCuneus | -0.09 | 0.48 | 0.03645 |
| Left Insula | Right Insula | 0.86 | 0.59 | 0.01953 |

**Table S16.** Cross-cortical correlations in the healthy controls group showing overlap between the significant inter-regional correlations identified using structural covariance methods in the Cambridge and Southampton samples when including the covariates in the statistical models

| Left Superior Frontal | Left Rostral Middle Frontal |
| --- | --- |
| Left Superior Frontal | Left Caudal Middle Frontal |
| Left Superior Frontal | Left ParsOpercularis |
| Left Superior Frontal | Left Orbital Frontal |
| Left Superior Frontal | Left Medial Orbital Frontal |
| Left Superior Frontal | Right Superior Frontal |
| Left Superior Frontal | Right Rostral Middle Frontal |
| Left Superior Frontal | Right Caudal Middle Frontal |
| Left Superior Frontal | Right ParsOpercularis |
| Left Superior Frontal | Right Orbital Frontal |
| Left Rostral Middle Frontal | Left Caudal Middle Frontal |
| Left Rostral Middle Frontal | Left ParsTriangularis |
| Left Rostral Middle Frontal | Right Superior Frontal |
| Left Rostral Middle Frontal | Right Rostral Middle Frontal |
| Left Rostral Middle Frontal | Right Caudal Middle Frontal |
| Left Rostral Middle Frontal | Right ParsOpercularis |
| Left Rostral Middle Frontal | Right ParsTriangularis |
| Left Caudal Middle Frontal | Right Superior Frontal |
| Left Caudal Middle Frontal | Right Rostral Middle Frontal |
| Left Caudal Middle Frontal | Right Caudal Middle Frontal |
| Left Caudal Middle Frontal | Right ParsOpercularis |
| Left ParsOrbitalis | Right Superior Frontal |
| Left Orbital Frontal | Right Superior Frontal |
| Left Orbital Frontal | Right Rostral Middle Frontal |
| Left Orbital Frontal | Right Caudal Middle Frontal |
| Left Orbital Frontal | Right ParsOpercularis |
| Left Orbital Frontal | Right Orbital Frontal |
| Left Orbital Frontal | Right Medial Orbital Frontal |
| Left Medial Orbital Frontal | Right Orbital Frontal |
| Left Medial Orbital Frontal | Right Medial Orbital Frontal |
| Right Superior Frontal | Right Rostral Middle Frontal |
| Right Superior Frontal | Right Caudal Middle Frontal |
| Right Superior Frontal | Right ParsOpercularis |
| Right Superior Frontal | Right ParsTriangularis |
| Right Rostral Middle Frontal | Right Caudal Middle Frontal |
| Right Rostral Middle Frontal | Right ParsOpercularis |
| Right Rostral Middle Frontal | Right ParsTriangularis |
| Right Caudal Middle Frontal | Right ParsOpercularis |
| Right Caudal Middle Frontal | Right ParsTriangularis |
| Right Caudal Middle Frontal | Right Orbital Frontal |
| Right Caudal Middle Frontal | Right PreCentral |
| Right ParsOpercularis | Right ParsTriangularis |
| Right ParsOpercularis | Right PreCentral |
| Right ParsOrbitalis | Right Orbital Frontal |
| Right Orbital Frontal | Right Medial Orbital Frontal |
| Right Medial Orbital Frontal | Right CA Cingulate |
| Left Superior Frontal | Right Superior Temporal |
| Left Superior Frontal | Right Middle Temporal |
| Left Rostral Middle Frontal | Right Superior Temporal |
| Left Rostral Middle Frontal | Right Middle Temporal |
| Left ParsTriangularis | Left Superior Temporal |
| Left ParsOrbitalis | Right Middle Temporal |
| Left Orbital Frontal | Right Fusiform |
| Left Orbital Frontal | Right Middle Temporal |
| Right Superior Frontal | Right Fusiform |
| Right Superior Frontal | Right Superior Temporal |
| Right Superior Frontal | Right Middle Temporal |
| Right Rostral Middle Frontal | Right Superior Temporal |
| Right Rostral Middle Frontal | Right Middle Temporal |
| Right Caudal Middle Frontal | Right Fusiform |
| Right Caudal Middle Frontal | Right Inferior Temporal |
| Right ParsTriangularis | Right Fusiform |
| Right ParsTriangularis | Right Superior Temporal |

| Right Orbital Frontal | Right Inferior Temporal |
| --- | --- |
| Left Superior Frontal | Left SupraMarginal |
| Left Superior Frontal | Left Superior Parietal |
| Left Superior Frontal | Right ParaCentral Lobule |
| Left Superior Frontal | Right Inferior Parietal |
| Left Rostral Middle Frontal | Left SupraMarginal |
| Left Rostral Middle Frontal | Left Inferior Parietal |
| Left Caudal Middle Frontal | Left PostCentral |
| Left Caudal Middle Frontal | Left Superior Parietal |
| Left Caudal Middle Frontal | Left Inferior Parietal |
| Left Caudal Middle Frontal | Right ParaCentral Lobule |
| Left Caudal Middle Frontal | Right Superior Parietal |
| Left Orbital Frontal | Right SupraMarginal |
| Left Orbital Frontal | Right Inferior Parietal |
| Right Superior Frontal | Right ParaCentral Lobule |
| Right Superior Frontal | Right SupraMarginal |
| Right Superior Frontal | Right Inferior Parietal |
| Right Rostral Middle Frontal | Right SupraMarginal |
| Right Rostral Middle Frontal | Right Inferior Parietal |
| Right Caudal Middle Frontal | Right ParaCentral Lobule |
| Right Caudal Middle Frontal | Right Inferior Parietal |
| Left Superior Frontal | Right PreCuneus |
| Left Orbital Frontal | Right PreCuneus |
| Right Superior Frontal | Right PreCuneus |
| Right Rostral Middle Frontal | Right PreCuneus |
| Right Caudal Middle Frontal | Right PreCuneus |
| Right ParsTriangularis | Right PreCuneus |
| Right ParsOrbitalis | Right PreCuneus |
| Right Orbital Frontal | Right PreCuneus |
| Left CA Cingulate | Left Insula |
| Right Orbital Frontal | Right Insula |
| Left Superior Temporal | Right Superior Frontal |
| Left Middle Temporal | Right Superior Frontal |
| Left Middle Temporal | Right Caudal Middle Frontal |
| Left Middle Temporal | Right ParsTriangularis |
| Left ParaCentral Lobule | Right Caudal Middle Frontal |
| Left SupraMarginal | Right Superior Frontal |
| Left SupraMarginal | Right Rostral Middle Frontal |
| Left SupraMarginal | Right Caudal Middle Frontal |
| Left SupraMarginal | Right ParsOpercularis |
| Left Superior Parietal | Right Superior Frontal |
| Left Superior Parietal | Right ParsOpercularis |
| Left Inferior Parietal | Right Superior Frontal |
| Left Inferior Parietal | Right Rostral Middle Frontal |
| Left Inferior Parietal | Right Caudal Middle Frontal |
| Left Inferior Parietal | Right ParsTriangularis |
| Left PreCuneus | Right Caudal Middle Frontal |
| Left PreCuneus | Right ParsOpercularis |
| Left Parahippocampal | Right Parahippocampal |
| Left Fusiform | Left Superior Temporal |
| Left Fusiform | Right Fusiform |
| Left Superior Temporal | Left Middle Temporal |
| Left Superior Temporal | Left Inferior Temporal |
| Left Superior Temporal | Right Superior Temporal |
| Left Superior Temporal | Right Middle Temporal |
| Left Superior Temporal | Right Inferior Temporal |
| Left Middle Temporal | Left Inferior Temporal |
| Left Middle Temporal | Right Fusiform |
| Left Middle Temporal | Right Superior Temporal |
| Left Middle Temporal | Right Middle Temporal |
| Left Middle Temporal | Right Inferior Temporal |
| Left Inferior Temporal | Right Fusiform |
| Left Inferior Temporal | Right Superior Temporal |
| Left Inferior Temporal | Right Middle Temporal |
| Left Inferior Temporal | Right Inferior Temporal |
| Left Banks Superior Temporal | Right Banks Superior Temporal |

| Right Fusiform | Right Superior Temporal |
| --- | --- |
| Right Fusiform | Right Middle Temporal |
| Right Fusiform | Right Inferior Temporal |
| Right Superior Temporal | Right Middle Temporal |
| Right Superior Temporal | Right Inferior Temporal |
| Right Middle Temporal | Right Inferior Temporal |
| Left Fusiform | Right Inferior Parietal |
| Left Superior Temporal | Right SupraMarginal |
| Left Superior Temporal | Right Inferior Parietal |
| Left Middle Temporal | Left PostCentral |
| Left Middle Temporal | Left SupraMarginal |
| Left Middle Temporal | Left Inferior Parietal |
| Left Middle Temporal | Right ParaCentral Lobule |
| Left Middle Temporal | Right Inferior Parietal |
| Left Inferior Temporal | Left Inferior Parietal |
| Right Superior Temporal | Right SupraMarginal |
| Right Inferior Temporal | Right Inferior Parietal |
| Right Banks Superior Temporal | Right Inferior Parietal |
| Left Middle Temporal | Left Lateral Occipital |
| Right Fusiform | Right PreCuneus |
| Right Fusiform | Right Lateral Occipital |
| Right Superior Temporal | Right PreCuneus |
| Right Superior Temporal | Right Lateral Occipital |
| Right Inferior Temporal | Right PreCuneus |
| Right Inferior Temporal | Right Lateral Occipital |
| Left ParaCentral Lobule | Right Fusiform |
| Left SupraMarginal | Right Superior Temporal |
| Left SupraMarginal | Right Middle Temporal |
| Left Inferior Parietal | Right Superior Temporal |
| Left Inferior Parietal | Right Middle Temporal |
| Right ParaCentral Lobule | Right Fusiform |
| Right ParaCentral Lobule | Right Superior Temporal |
| Right ParaCentral Lobule | Right Middle Temporal |
| Left Lateral Occipital | Right Fusiform |
| Left Lateral Occipital | Right Inferior Temporal |
| Left Insula | Left Superior Temporal |
| Left Insula | Left Inferior Temporal |
| Left Insula | Right Inferior Temporal |
| Right Insula | Right Fusiform |
| Left ParaCentral Lobule | Left PostCentral |
| Left ParaCentral Lobule | Right ParaCentral Lobule |
| Left PostCentral | Left Superior Parietal |
| Left PostCentral | Left Inferior Parietal |
| Left PostCentral | Right ParaCentral Lobule |
| Left PostCentral | Right PostCentral |
| Left PostCentral | Right SupraMarginal |
| Left SupraMarginal | Left Inferior Parietal |
| Left SupraMarginal | Right SupraMarginal |
| Left SupraMarginal | Right Inferior Parietal |
| Left Superior Parietal | Left Inferior Parietal |
| Left Superior Parietal | Right Superior Parietal |
| Left Superior Parietal | Right Inferior Parietal |
| Left Inferior Parietal | Right ParaCentral Lobule |
| Left Inferior Parietal | Right Superior Parietal |
| Left Inferior Parietal | Right Inferior Parietal |
| Right ParaCentral Lobule | Right SupraMarginal |
| Right ParaCentral Lobule | Right Inferior Parietal |
| Right Superior Parietal | Right Inferior Parietal |
| Left ParaCentral Lobule | Left Lateral Occipital |
| Left ParaCentral Lobule | Right Lateral Occipital |
| Left PostCentral | Left PreCuneus |
| Left PostCentral | Right PreCuneus |
| Left PostCentral | Right Lateral Occipital |
| Left SupraMarginal | Right PreCuneus |
| Left Superior Parietal | Left PreCuneus |
| Left Superior Parietal | Left Lateral Occipital |

| Left Superior Parietal | Right PreCuneus |
| --- | --- |
| Left Inferior Parietal | Left Lateral Occipital |
| Left Inferior Parietal | Right PreCuneus |
| Right ParaCentral Lobule | Right Lateral Occipital |
| Right Superior Parietal | Right PreCuneus |
| Right Superior Parietal | Right Lateral Occipital |
| Right Inferior Parietal | Right PreCuneus |
| Left PreCuneus | Right Superior Parietal |
| Left PreCuneus | Right Inferior Parietal |
| Left Lingual | Right Isthmus |
| Left Lingual | Right Inferior Parietal |
| Left Cuneus | Right Superior Parietal |
| Left Lateral Occipital | Right Superior Parietal |
| Left Lateral Occipital | Right Inferior Parietal |
| Left PreCuneus | Left Lingual |
| Left PreCuneus | Left Cuneus |
| Left PreCuneus | Left Lateral Occipital |
| Left PreCuneus | Right PreCuneus |
| Left PreCuneus | Right Cuneus |
| Left PreCuneus | Right Lateral Occipital |
| Left Lingual | Right Lingual |
| Left Pericalcarine | Left Cuneus |
| Left Pericalcarine | Right Pericalcarine |
| Left Pericalcarine | Right Lateral Occipital |
| Left Lateral Occipital | Right PreCuneus |
| Left Lateral Occipital | Right Lateral Occipital |
| Right PreCuneus | Right Lateral Occipital |

**Table S17.** Cross-cortical correlations in the childhood-onset conduct disorder (CO-CD) group showing overlap between the significant inter-regional correlations identified using structural covariance methods in the Cambridge and Southampton samples when including the covariates in the statistical models

| Left Superior Frontal | Left Rostral Middle Frontal |
| --- | --- |
| Left Superior Frontal | Left Caudal Middle Frontal |
| Left Superior Frontal | Left ParsOpercularis |
| Left Superior Frontal | Left ParsTriangularis |
| Left Superior Frontal | Left Orbital Frontal |
| Left Superior Frontal | Left PreCentral |
| Left Superior Frontal | Right Superior Frontal |
| Left Superior Frontal | Right Rostral Middle Frontal |
| Left Superior Frontal | Right Caudal Middle Frontal |
| Left Superior Frontal | Right ParsOpercularis |
| Left Superior Frontal | Right ParsTriangularis |
| Left Superior Frontal | Right Orbital Frontal |
| Left Superior Frontal | Right Medial Orbital Frontal |
| Left Superior Frontal | Right PreCentral |
| Left Superior Frontal | Right CA Cingulate |
| Left Rostral Middle Frontal | Left Caudal Middle Frontal |
| Left Rostral Middle Frontal | Left ParsOpercularis |
| Left Rostral Middle Frontal | Left ParsTriangularis |
| Left Rostral Middle Frontal | Left Orbital Frontal |
| Left Rostral Middle Frontal | Right Superior Frontal |
| Left Rostral Middle Frontal | Right Rostral Middle Frontal |
| Left Rostral Middle Frontal | Right ParsOpercularis |
| Left Caudal Middle Frontal | Left ParsOpercularis |
| Left Caudal Middle Frontal | Left ParsTriangularis |
| Left Caudal Middle Frontal | Left Orbital Frontal |
| Left Caudal Middle Frontal | Left PreCentral |
| Left Caudal Middle Frontal | Right Superior Frontal |
| Left Caudal Middle Frontal | Right Rostral Middle Frontal |
| Left Caudal Middle Frontal | Right Caudal Middle Frontal |
| Left Caudal Middle Frontal | Right ParsOpercularis |
| Left Caudal Middle Frontal | Right ParsTriangularis |
| Left Caudal Middle Frontal | Right PreCentral |
| Left ParsOpercularis | Left ParsTriangularis |
| Left ParsOpercularis | Left Orbital Frontal |

| Left ParsOpercularis | Left PreCentral |
| --- | --- |
| Left ParsOpercularis | Right Superior Frontal |
| Left ParsOpercularis | Right Rostral Middle Frontal |
| Left ParsOpercularis | Right Caudal Middle Frontal |
| Left ParsOpercularis | Right ParsOpercularis |
| Left ParsOpercularis | Right ParsTriangularis |
| Left ParsOpercularis | Right Orbital Frontal |
| Left ParsOpercularis | Right PreCentral |
| Left ParsTriangularis | Left Orbital Frontal |
| Left ParsTriangularis | Left PreCentral |
| Left ParsTriangularis | Right Superior Frontal |
| Left ParsTriangularis | Right Rostral Middle Frontal |
| Left ParsTriangularis | Right Caudal Middle Frontal |
| Left ParsTriangularis | Right ParsOpercularis |
| Left ParsTriangularis | Right ParsTriangularis |
| Left ParsTriangularis | Right Orbital Frontal |
| Left ParsTriangularis | Right PreCentral |
| Left ParsOrbitalis | Right Frontal Pole |
| Left Orbital Frontal | Left Medial Orbital Frontal |
| Left Orbital Frontal | Left Frontal Pole |
| Left Orbital Frontal | Left PreCentral |
| Left Orbital Frontal | Right Superior Frontal |
| Left Orbital Frontal | Right Caudal Middle Frontal |
| Left Orbital Frontal | Right Orbital Frontal |
| Left Orbital Frontal | Right PreCentral |
| Left Orbital Frontal | Right CA Cingulate |
| Left Frontal Pole | Left CA Cingulate |
| Left PreCentral | Right Superior Frontal |
| Left PreCentral | Right Caudal Middle Frontal |
| Left PreCentral | Right ParsOpercularis |
| Left PreCentral | Right ParsTriangularis |
| Left PreCentral | Right PreCentral |
| Left CA Cingulate | Right CA Cingulate |
| Right Superior Frontal | Right Rostral Middle Frontal |
| Right Superior Frontal | Right Caudal Middle Frontal |
| Right Superior Frontal | Right ParsOpercularis |
| Right Superior Frontal | Right ParsTriangularis |
| Right Superior Frontal | Right Orbital Frontal |
| Right Superior Frontal | Right Medial Orbital Frontal |
| Right Superior Frontal | Right PreCentral |
| Right Rostral Middle Frontal | Right Caudal Middle Frontal |
| Right Rostral Middle Frontal | Right ParsOpercularis |
| Right Rostral Middle Frontal | Right ParsTriangularis |
| Right Rostral Middle Frontal | Right Orbital Frontal |
| Right Rostral Middle Frontal | Right Medial Orbital Frontal |
| Right Caudal Middle Frontal | Right ParsOpercularis |
| Right Caudal Middle Frontal | Right Orbital Frontal |
| Right Caudal Middle Frontal | Right PreCentral |
| Right ParsOpercularis | Right ParsTriangularis |
| Right ParsOpercularis | Right Orbital Frontal |
| Right ParsOpercularis | Right PreCentral |
| Right ParsTriangularis | Right ParsOrbitalis |
| Right ParsTriangularis | Right Orbital Frontal |
| Right ParsTriangularis | Right PreCentral |
| Right ParsOrbitalis | Right Orbital Frontal |
| Right Orbital Frontal | Right PreCentral |
| Left Superior Frontal | Left Fusiform |
| Left Superior Frontal | Left Superior Temporal |
| Left Superior Frontal | Left Middle Temporal |
| Left Superior Frontal | Left Inferior Temporal |
| Left Superior Frontal | Right Entorhinal |
| Left Superior Frontal | Right Parahippocampal |
| Left Superior Frontal | Right Temporal Pole |
| Left Superior Frontal | Right Superior Temporal |
| Left Superior Frontal | Right Middle Temporal |
| Left Superior Frontal | Right Inferior Temporal |

| Left Rostral Middle Frontal | Left Superior Temporal |
| --- | --- |
| Left Rostral Middle Frontal | Right Superior Temporal |
| Left Rostral Middle Frontal | Right Inferior Temporal |
| Left Caudal Middle Frontal | Left Superior Temporal |
| Left Caudal Middle Frontal | Left Middle Temporal |
| Left Caudal Middle Frontal | Left Inferior Temporal |
| Left Caudal Middle Frontal | Right Parahippocampal |
| Left Caudal Middle Frontal | Right Temporal Pole |
| Left Caudal Middle Frontal | Right Superior Temporal |
| Left Caudal Middle Frontal | Right Middle Temporal |
| Left Caudal Middle Frontal | Right Inferior Temporal |
| Left ParsOpercularis | Left Fusiform |
| Left ParsOpercularis | Left Superior Temporal |
| Left ParsOpercularis | Left Middle Temporal |
| Left ParsOpercularis | Left Inferior Temporal |
| Left ParsOpercularis | Left Transverse Temporal |
| Left ParsOpercularis | Right Entorhinal |
| Left ParsOpercularis | Right Superior Temporal |
| Left ParsOpercularis | Right Middle Temporal |
| Left ParsOpercularis | Right Inferior Temporal |
| Left ParsOpercularis | Right Banks Superior Temporal |
| Left ParsTriangularis | Left Fusiform |
| Left ParsTriangularis | Left Superior Temporal |
| Left ParsTriangularis | Left Middle Temporal |
| Left ParsTriangularis | Left Inferior Temporal |
| Left ParsTriangularis | Right Entorhinal |
| Left ParsTriangularis | Right Temporal Pole |
| Left ParsTriangularis | Right Fusiform |
| Left ParsTriangularis | Right Superior Temporal |
| Left ParsTriangularis | Right Middle Temporal |
| Left Orbital Frontal | Left Temporal Pole |
| Left Orbital Frontal | Left Fusiform |
| Left Orbital Frontal | Left Superior Temporal |
| Left Orbital Frontal | Left Middle Temporal |
| Left Orbital Frontal | Left Inferior Temporal |
| Left Orbital Frontal | Right Entorhinal |
| Left Orbital Frontal | Right Parahippocampal |
| Left Orbital Frontal | Right Temporal Pole |
| Left Orbital Frontal | Right Superior Temporal |
| Left Orbital Frontal | Right Middle Temporal |
| Left Orbital Frontal | Right Inferior Temporal |
| Left PreCentral | Left Superior Temporal |
| Left PreCentral | Left Middle Temporal |
| Left PreCentral | Right Entorhinal |
| Left PreCentral | Right Superior Temporal |
| Left PreCentral | Right Middle Temporal |
| Left PreCentral | Right Inferior Temporal |
| Right Superior Frontal | Right Temporal Pole |
| Right Superior Frontal | Right Fusiform |
| Right Superior Frontal | Right Superior Temporal |
| Right Superior Frontal | Right Middle Temporal |
| Right Superior Frontal | Right Inferior Temporal |
| Right Rostral Middle Frontal | Right Inferior Temporal |
| Right Caudal Middle Frontal | Right Temporal Pole |
| Right Caudal Middle Frontal | Right Superior Temporal |
| Right ParsOpercularis | Right Middle Temporal |
| Right ParsOpercularis | Right Inferior Temporal |
| Right ParsTriangularis | Right Entorhinal |
| Right ParsTriangularis | Right Parahippocampal |
| Right ParsTriangularis | Right Temporal Pole |
| Right ParsTriangularis | Right Fusiform |
| Right ParsTriangularis | Right Superior Temporal |
| Right ParsTriangularis | Right Middle Temporal |
| Right ParsTriangularis | Right Inferior Temporal |
| Right ParsTriangularis | Right Banks Superior Temporal |
| Right Orbital Frontal | Right Entorhinal |

| Right Orbital Frontal | Right Temporal Pole |
| --- | --- |
| Right Orbital Frontal | Right Fusiform |
| Right Orbital Frontal | Right Superior Temporal |
| Right Orbital Frontal | Right Middle Temporal |
| Right Orbital Frontal | Right Inferior Temporal |
| Right Orbital Frontal | Right Transverse Temporal |
| Right PreCentral | Right Entorhinal |
| Right PreCentral | Right Parahippocampal |
| Right PreCentral | Right Temporal Pole |
| Right PreCentral | Right Fusiform |
| Right PreCentral | Right Superior Temporal |
| Right PreCentral | Right Middle Temporal |
| Right PreCentral | Right Inferior Temporal |
| Right PreCentral | Right Transverse Temporal |
| Right PreCentral | Right Banks Superior Temporal |
| Left Superior Frontal | Left ParaCentral Lobule |
| Left Superior Frontal | Left PostCentral |
| Left Superior Frontal | Left SupraMarginal |
| Left Superior Frontal | Left Superior Parietal |
| Left Superior Frontal | Left Inferior Parietal |
| Left Superior Frontal | Right ParaCentral Lobule |
| Left Superior Frontal | Right SupraMarginal |
| Left Superior Frontal | Right Superior Parietal |
| Left Superior Frontal | Right Inferior Parietal |
| Left Rostral Middle Frontal | Left SupraMarginal |
| Left Rostral Middle Frontal | Left Superior Parietal |
| Left Rostral Middle Frontal | Left Inferior Parietal |
| Left Rostral Middle Frontal | Right SupraMarginal |
| Left Rostral Middle Frontal | Right Inferior Parietal |
| Left Caudal Middle Frontal | Left ParaCentral Lobule |
| Left Caudal Middle Frontal | Left PostCentral |
| Left Caudal Middle Frontal | Left SupraMarginal |
| Left Caudal Middle Frontal | Left Superior Parietal |
| Left Caudal Middle Frontal | Left Inferior Parietal |
| Left Caudal Middle Frontal | Right SupraMarginal |
| Left Caudal Middle Frontal | Right Superior Parietal |
| Left Caudal Middle Frontal | Right Inferior Parietal |
| Left ParsOpercularis | Left ParaCentral Lobule |
| Left ParsOpercularis | Left PostCentral |
| Left ParsOpercularis | Left SupraMarginal |
| Left ParsOpercularis | Left Inferior Parietal |
| Left ParsOpercularis | Right ParaCentral Lobule |
| Left ParsOpercularis | Right SupraMarginal |
| Left ParsOpercularis | Right Inferior Parietal |
| Left ParsTriangularis | Left SupraMarginal |
| Left ParsTriangularis | Left Superior Parietal |
| Left ParsTriangularis | Left Inferior Parietal |
| Left ParsTriangularis | Right ParaCentral Lobule |
| Left ParsTriangularis | Right Superior Parietal |
| Left ParsTriangularis | Right Inferior Parietal |
| Left Orbital Frontal | Left ParaCentral Lobule |
| Left Orbital Frontal | Left SupraMarginal |
| Left Orbital Frontal | Left Superior Parietal |
| Left Orbital Frontal | Left Inferior Parietal |
| Left Orbital Frontal | Right ParaCentral Lobule |
| Left Orbital Frontal | Right Posterior Cingulate |
| Left Orbital Frontal | Right SupraMarginal |
| Left Orbital Frontal | Right Superior Parietal |
| Left Orbital Frontal | Right Inferior Parietal |
| Left PreCentral | Left ParaCentral Lobule |
| Left PreCentral | Left PostCentral |
| Left PreCentral | Left SupraMarginal |
| Left PreCentral | Left Superior Parietal |
| Left PreCentral | Right ParaCentral Lobule |
| Left PreCentral | Right SupraMarginal |
| Left PreCentral | Right Superior Parietal |

| Left PreCentral | Right Inferior Parietal |
| --- | --- |
| Right Superior Frontal | Right ParaCentral Lobule |
| Right Superior Frontal | Right PostCentral |
| Right Superior Frontal | Right SupraMarginal |
| Right Superior Frontal | Right Superior Parietal |
| Right Superior Frontal | Right Inferior Parietal |
| Right Rostral Middle Frontal | Right SupraMarginal |
| Right Rostral Middle Frontal | Right Superior Parietal |
| Right Caudal Middle Frontal | Right ParaCentral Lobule |
| Right Caudal Middle Frontal | Right PostCentral |
| Right Caudal Middle Frontal | Right SupraMarginal |
| Right Caudal Middle Frontal | Right Superior Parietal |
| Right Caudal Middle Frontal | Right Inferior Parietal |
| Right ParsOpercularis | Right PostCentral |
| Right ParsOpercularis | Right SupraMarginal |
| Right ParsOpercularis | Right Inferior Parietal |
| Right ParsTriangularis | Right ParaCentral Lobule |
| Right ParsTriangularis | Right PostCentral |
| Right ParsTriangularis | Right SupraMarginal |
| Right ParsTriangularis | Right Superior Parietal |
| Right ParsTriangularis | Right Inferior Parietal |
| Right Orbital Frontal | Right ParaCentral Lobule |
| Right Orbital Frontal | Right PostCentral |
| Right Orbital Frontal | Right SupraMarginal |
| Right Orbital Frontal | Right Superior Parietal |
| Right Orbital Frontal | Right Inferior Parietal |
| Right PreCentral | Right ParaCentral Lobule |
| Right PreCentral | Right SupraMarginal |
| Right PreCentral | Right Superior Parietal |
| Right PreCentral | Right Inferior Parietal |
| Left Superior Frontal | Left PreCuneus |
| Left Superior Frontal | Left Cuneus |
| Left Superior Frontal | Left Lateral Occipital |
| Left Superior Frontal | Right PreCuneus |
| Left Superior Frontal | Right Lateral Occipital |
| Left Rostral Middle Frontal | Left PreCuneus |
| Left Rostral Middle Frontal | Right Pericalcarine |
| Left Rostral Middle Frontal | Right Lateral Occipital |
| Left Caudal Middle Frontal | Left PreCuneus |
| Left Caudal Middle Frontal | Left Pericalcarine |
| Left Caudal Middle Frontal | Left Lateral Occipital |
| Left Caudal Middle Frontal | Right Pericalcarine |
| Left Caudal Middle Frontal | Right Lateral Occipital |
| Left ParsOpercularis | Left PreCuneus |
| Left ParsOpercularis | Left Lateral Occipital |
| Left ParsOpercularis | Right Lateral Occipital |
| Left ParsTriangularis | Left PreCuneus |
| Left ParsTriangularis | Left Lingual |
| Left ParsTriangularis | Left Lateral Occipital |
| Left ParsTriangularis | Right Lingual |
| Left ParsTriangularis | Right Lateral Occipital |
| Left Orbital Frontal | Left PreCuneus |
| Left Orbital Frontal | Left Lateral Occipital |
| Left Orbital Frontal | Right PreCuneus |
| Left Orbital Frontal | Right Lateral Occipital |
| Left PreCentral | Left PreCuneus |
| Left PreCentral | Left Lateral Occipital |
| Right Superior Frontal | Right PreCuneus |
| Right Superior Frontal | Right Lateral Occipital |
| Right Rostral Middle Frontal | Right Lateral Occipital |
| Right Caudal Middle Frontal | Right PreCuneus |
| Right ParsTriangularis | Right PreCuneus |
| Right ParsTriangularis | Right Lateral Occipital |
| Right Orbital Frontal | Right PreCuneus |
| Right Orbital Frontal | Right Lateral Occipital |
| Right PreCentral | Right PreCuneus |

| Right PreCentral | Right Lingual |
| --- | --- |
| Right PreCentral | Right Lateral Occipital |
| Left Superior Frontal | Left Insula |
| Left Superior Frontal | Right Insula |
| Left Caudal Middle Frontal | Left Insula |
| Left ParsOpercularis | Left Insula |
| Left ParsOpercularis | Right Insula |
| Left ParsTriangularis | Left Insula |
| Left ParsTriangularis | Right Insula |
| Left ParsOrbitalis | Left Insula |
| Left Orbital Frontal | Left Insula |
| Left Orbital Frontal | Right Insula |
| Left PreCentral | Left Insula |
| Right Superior Frontal | Right Insula |
| Right Rostral Middle Frontal | Right Insula |
| Right Caudal Middle Frontal | Right Insula |
| Right ParsOpercularis | Right Insula |
| Right ParsTriangularis | Right Insula |
| Right Orbital Frontal | Right Insula |
| Right PreCentral | Right Insula |
| Left Entorhinal | Right Caudal Middle Frontal |
| Left Fusiform | Right ParsOpercularis |
| Left Fusiform | Right Orbital Frontal |
| Left Fusiform | Right PreCentral |
| Left Fusiform | Right RA cingulate |
| Left Superior Temporal | Right Superior Frontal |
| Left Superior Temporal | Right Rostral Middle Frontal |
| Left Superior Temporal | Right Caudal Middle Frontal |
| Left Superior Temporal | Right ParsTriangularis |
| Left Superior Temporal | Right Orbital Frontal |
| Left Superior Temporal | Right PreCentral |
| Left Middle Temporal | Right Superior Frontal |
| Left Middle Temporal | Right Caudal Middle Frontal |
| Left Middle Temporal | Right ParsOpercularis |
| Left Middle Temporal | Right ParsTriangularis |
| Left Middle Temporal | Right Orbital Frontal |
| Left Middle Temporal | Right PreCentral |
| Left Inferior Temporal | Right ParsTriangularis |
| Left Inferior Temporal | Right PreCentral |
| Left ParaCentral Lobule | Right Superior Frontal |
| Left ParaCentral Lobule | Right Caudal Middle Frontal |
| Left ParaCentral Lobule | Right PreCentral |
| Left PostCentral | Right Superior Frontal |
| Left PostCentral | Right Caudal Middle Frontal |
| Left PostCentral | Right ParsOpercularis |
| Left PostCentral | Right ParsTriangularis |
| Left PostCentral | Right Orbital Frontal |
| Left PostCentral | Right PreCentral |
| Left SupraMarginal | Right Superior Frontal |
| Left SupraMarginal | Right Caudal Middle Frontal |
| Left SupraMarginal | Right ParsOpercularis |
| Left SupraMarginal | Right ParsTriangularis |
| Left SupraMarginal | Right Orbital Frontal |
| Left SupraMarginal | Right PreCentral |
| Left Superior Parietal | Right Superior Frontal |
| Left Superior Parietal | Right Rostral Middle Frontal |
| Left Superior Parietal | Right Caudal Middle Frontal |
| Left Superior Parietal | Right ParsOpercularis |
| Left Superior Parietal | Right ParsTriangularis |
| Left Superior Parietal | Right Orbital Frontal |
| Left Superior Parietal | Right PreCentral |
| Left Inferior Parietal | Right Superior Frontal |
| Left Inferior Parietal | Right Caudal Middle Frontal |
| Left Inferior Parietal | Right ParsOpercularis |
| Left Inferior Parietal | Right ParsTriangularis |
| Left Inferior Parietal | Right Orbital Frontal |

| Left Inferior Parietal | Right PreCentral |
| --- | --- |
| Left PreCuneus | Right Superior Frontal |
| Left PreCuneus | Right Caudal Middle Frontal |
| Left PreCuneus | Right ParsTriangularis |
| Left PreCuneus | Right Orbital Frontal |
| Left PreCuneus | Right PreCentral |
| Left Lingual | Right ParsOpercularis |
| Left Lingual | Right PreCentral |
| Left Lateral Occipital | Right Superior Frontal |
| Left Lateral Occipital | Right ParsOpercularis |
| Left Lateral Occipital | Right ParsTriangularis |
| Left Lateral Occipital | Right PreCentral |
| Left Insula | Right Caudal Middle Frontal |
| Left Insula | Right ParsTriangularis |
| Left Insula | Right Orbital Frontal |
| Left Insula | Right PreCentral |
| Left Entorhinal | Left Temporal Pole |
| Left Entorhinal | Right Entorhinal |
| Left Entorhinal | Right Temporal Pole |
| Left Parahippocampal | Right Parahippocampal |
| Left Temporal Pole | Left Fusiform |
| Left Temporal Pole | Left Superior Temporal |
| Left Temporal Pole | Left Middle Temporal |
| Left Temporal Pole | Left Inferior Temporal |
| Left Temporal Pole | Right Temporal Pole |
| Left Temporal Pole | Right Middle Temporal |
| Left Temporal Pole | Right Inferior Temporal |
| Left Fusiform | Left Superior Temporal |
| Left Fusiform | Left Middle Temporal |
| Left Fusiform | Left Inferior Temporal |
| Left Fusiform | Left Banks Superior Temporal |
| Left Fusiform | Right Entorhinal |
| Left Fusiform | Right Superior Temporal |
| Left Fusiform | Right Middle Temporal |
| Left Fusiform | Right Inferior Temporal |
| Left Fusiform | Right Banks Superior Temporal |
| Left Superior Temporal | Left Middle Temporal |
| Left Superior Temporal | Left Inferior Temporal |
| Left Superior Temporal | Right Parahippocampal |
| Left Superior Temporal | Right Temporal Pole |
| Left Superior Temporal | Right Fusiform |
| Left Superior Temporal | Right Superior Temporal |
| Left Superior Temporal | Right Middle Temporal |
| Left Superior Temporal | Right Inferior Temporal |
| Left Superior Temporal | Right Transverse Temporal |
| Left Superior Temporal | Right Banks Superior Temporal |
| Left Middle Temporal | Left Inferior Temporal |
| Left Middle Temporal | Left Transverse Temporal |
| Left Middle Temporal | Left Banks Superior Temporal |
| Left Middle Temporal | Right Temporal Pole |
| Left Middle Temporal | Right Fusiform |
| Left Middle Temporal | Right Superior Temporal |
| Left Middle Temporal | Right Middle Temporal |
| Left Middle Temporal | Right Inferior Temporal |
| Left Middle Temporal | Right Banks Superior Temporal |
| Left Inferior Temporal | Right Entorhinal |
| Left Inferior Temporal | Right Parahippocampal |
| Left Inferior Temporal | Right Temporal Pole |
| Left Inferior Temporal | Right Fusiform |
| Left Inferior Temporal | Right Superior Temporal |
| Left Inferior Temporal | Right Middle Temporal |
| Left Inferior Temporal | Right Inferior Temporal |
| Left Inferior Temporal | Right Banks Superior Temporal |
| Left Transverse Temporal | Right Superior Temporal |
| Left Transverse Temporal | Right Inferior Temporal |
| Left Transverse Temporal | Right Transverse Temporal |

| Right Entorhinal | Right Temporal Pole |
| --- | --- |
| Right Entorhinal | Right Fusiform |
| Right Entorhinal | Right Middle Temporal |
| Right Entorhinal | Right Inferior Temporal |
| Right Entorhinal | Right Banks Superior Temporal |
| Right Parahippocampal | Right Superior Temporal |
| Right Parahippocampal | Right Middle Temporal |
| Right Temporal Pole | Right Superior Temporal |
| Right Temporal Pole | Right Middle Temporal |
| Right Temporal Pole | Right Inferior Temporal |
| Right Fusiform | Right Superior Temporal |
| Right Fusiform | Right Middle Temporal |
| Right Fusiform | Right Inferior Temporal |
| Right Fusiform | Right Banks Superior Temporal |
| Right Superior Temporal | Right Middle Temporal |
| Right Superior Temporal | Right Inferior Temporal |
| Right Superior Temporal | Right Transverse Temporal |
| Right Superior Temporal | Right Banks Superior Temporal |
| Right Middle Temporal | Right Inferior Temporal |
| Right Middle Temporal | Right Banks Superior Temporal |
| Right Inferior Temporal | Right Banks Superior Temporal |
| Right Transverse Temporal | Right Banks Superior Temporal |
| Left Fusiform | Left SupraMarginal |
| Left Fusiform | Left Inferior Parietal |
| Left Fusiform | Right SupraMarginal |
| Left Fusiform | Right Inferior Parietal |
| Left Superior Temporal | Left PostCentral |
| Left Superior Temporal | Left SupraMarginal |
| Left Superior Temporal | Left Superior Parietal |
| Left Superior Temporal | Left Inferior Parietal |
| Left Superior Temporal | Right ParaCentral Lobule |
| Left Superior Temporal | Right PostCentral |
| Left Superior Temporal | Right SupraMarginal |
| Left Superior Temporal | Right Superior Parietal |
| Left Superior Temporal | Right Inferior Parietal |
| Left Middle Temporal | Left PostCentral |
| Left Middle Temporal | Left SupraMarginal |
| Left Middle Temporal | Left Superior Parietal |
| Left Middle Temporal | Left Inferior Parietal |
| Left Middle Temporal | Right ParaCentral Lobule |
| Left Middle Temporal | Right SupraMarginal |
| Left Middle Temporal | Right Superior Parietal |
| Left Middle Temporal | Right Inferior Parietal |
| Left Inferior Temporal | Left SupraMarginal |
| Left Inferior Temporal | Left Superior Parietal |
| Left Inferior Temporal | Left Inferior Parietal |
| Left Inferior Temporal | Right SupraMarginal |
| Left Inferior Temporal | Right Inferior Parietal |
| Right Entorhinal | Right SupraMarginal |
| Right Entorhinal | Right Inferior Parietal |
| Right Temporal Pole | Right SupraMarginal |
| Right Temporal Pole | Right Superior Parietal |
| Right Temporal Pole | Right Inferior Parietal |
| Right Fusiform | Right SupraMarginal |
| Right Fusiform | Right Inferior Parietal |
| Right Superior Temporal | Right PostCentral |
| Right Superior Temporal | Right SupraMarginal |
| Right Superior Temporal | Right Superior Parietal |
| Right Superior Temporal | Right Inferior Parietal |
| Right Middle Temporal | Right SupraMarginal |
| Right Middle Temporal | Right Superior Parietal |
| Right Middle Temporal | Right Inferior Parietal |
| Right Inferior Temporal | Right SupraMarginal |
| Right Inferior Temporal | Right Superior Parietal |
| Right Inferior Temporal | Right Inferior Parietal |
| Right Banks Superior Temporal | Right SupraMarginal |

| Right Banks Superior Temporal | Right Superior Parietal |
| --- | --- |
| Right Banks Superior Temporal | Right Inferior Parietal |
| Left Fusiform | Left PreCuneus |
| Left Fusiform | Left Lateral Occipital |
| Left Fusiform | Right PreCuneus |
| Left Fusiform | Right Lateral Occipital |
| Left Superior Temporal | Left PreCuneus |
| Left Superior Temporal | Left Lateral Occipital |
| Left Superior Temporal | Right PreCuneus |
| Left Superior Temporal | Right Lateral Occipital |
| Left Middle Temporal | Left PreCuneus |
| Left Middle Temporal | Left Lateral Occipital |
| Left Middle Temporal | Right PreCuneus |
| Left Middle Temporal | Right Lateral Occipital |
| Left Inferior Temporal | Left PreCuneus |
| Left Inferior Temporal | Left Lateral Occipital |
| Left Inferior Temporal | Right PreCuneus |
| Left Inferior Temporal | Right Lateral Occipital |
| Left Banks Superior Temporal | Left Lingual |
| Right Entorhinal | Right Lingual |
| Right Entorhinal | Right Lateral Occipital |
| Right Temporal Pole | Right PreCuneus |
| Right Fusiform | Right PreCuneus |
| Right Fusiform | Right Lingual |
| Right Fusiform | Right Lateral Occipital |
| Right Superior Temporal | Right PreCuneus |
| Right Superior Temporal | Right Lateral Occipital |
| Right Middle Temporal | Right PreCuneus |
| Right Middle Temporal | Right Lingual |
| Right Middle Temporal | Right Lateral Occipital |
| Right Inferior Temporal | Right PreCuneus |
| Right Inferior Temporal | Right Lingual |
| Right Inferior Temporal | Right Lateral Occipital |
| Right Banks Superior Temporal | Right PreCuneus |
| Right Banks Superior Temporal | Right Lingual |
| Left Fusiform | Right Insula |
| Left Superior Temporal | Right Insula |
| Left Middle Temporal | Right Insula |
| Left Inferior Temporal | Right Insula |
| Left ParaCentral Lobule | Right Temporal Pole |
| Left ParaCentral Lobule | Right Superior Temporal |
| Left ParaCentral Lobule | Right Inferior Temporal |
| Left PostCentral | Right Temporal Pole |
| Left PostCentral | Right Superior Temporal |
| Left PostCentral | Right Middle Temporal |
| Left SupraMarginal | Right Parahippocampal |
| Left SupraMarginal | Right Temporal Pole |
| Left SupraMarginal | Right Fusiform |
| Left SupraMarginal | Right Superior Temporal |
| Left SupraMarginal | Right Middle Temporal |
| Left SupraMarginal | Right Inferior Temporal |
| Left SupraMarginal | Right Banks Superior Temporal |
| Left Superior Parietal | Right Temporal Pole |
| Left Superior Parietal | Right Fusiform |
| Left Superior Parietal | Right Superior Temporal |
| Left Superior Parietal | Right Middle Temporal |
| Left Superior Parietal | Right Inferior Temporal |
| Left Superior Parietal | Right Banks Superior Temporal |
| Left Inferior Parietal | Right Entorhinal |
| Left Inferior Parietal | Right Temporal Pole |
| Left Inferior Parietal | Right Fusiform |
| Left Inferior Parietal | Right Superior Temporal |
| Left Inferior Parietal | Right Middle Temporal |
| Left Inferior Parietal | Right Inferior Temporal |
| Left Inferior Parietal | Right Banks Superior Temporal |
| Right ParaCentral Lobule | Right Entorhinal |

| Right ParaCentral Lobule | Right Temporal Pole |
| --- | --- |
| Right ParaCentral Lobule | Right Fusiform |
| Right ParaCentral Lobule | Right Superior Temporal |
| Right ParaCentral Lobule | Right Middle Temporal |
| Right ParaCentral Lobule | Right Banks Superior Temporal |
| Left PreCuneus | Right Entorhinal |
| Left PreCuneus | Right Temporal Pole |
| Left PreCuneus | Right Fusiform |
| Left PreCuneus | Right Superior Temporal |
| Left PreCuneus | Right Middle Temporal |
| Left PreCuneus | Right Inferior Temporal |
| Left PreCuneus | Right Banks Superior Temporal |
| Left Lingual | Right Middle Temporal |
| Left Lingual | Right Inferior Temporal |
| Left Pericalcarine | Right Inferior Temporal |
| Left Lateral Occipital | Right Entorhinal |
| Left Lateral Occipital | Right Fusiform |
| Left Lateral Occipital | Right Superior Temporal |
| Left Lateral Occipital | Right Middle Temporal |
| Left Lateral Occipital | Right Inferior Temporal |
| Left Lateral Occipital | Right Banks Superior Temporal |
| Left Insula | Left Superior Temporal |
| Left Insula | Left Middle Temporal |
| Left Insula | Right Entorhinal |
| Left Insula | Right Temporal Pole |
| Left Insula | Right Superior Temporal |
| Left Insula | Right Middle Temporal |
| Right Insula | Right Temporal Pole |
| Right Insula | Right Middle Temporal |
| Left ParaCentral Lobule | Left PostCentral |
| Left ParaCentral Lobule | Left SupraMarginal |
| Left ParaCentral Lobule | Left Superior Parietal |
| Left ParaCentral Lobule | Left Inferior Parietal |
| Left ParaCentral Lobule | Right ParaCentral Lobule |
| Left ParaCentral Lobule | Right SupraMarginal |
| Left ParaCentral Lobule | Right Superior Parietal |
| Left ParaCentral Lobule | Right Inferior Parietal |
| Left Posterior Cingulate | Right Posterior Cingulate |
| Left Isthmus | Right Isthmus |
| Left PostCentral | Left SupraMarginal |
| Left PostCentral | Left Superior Parietal |
| Left PostCentral | Left Inferior Parietal |
| Left PostCentral | Right ParaCentral Lobule |
| Left PostCentral | Right PostCentral |
| Left PostCentral | Right SupraMarginal |
| Left PostCentral | Right Superior Parietal |
| Left PostCentral | Right Inferior Parietal |
| Left SupraMarginal | Left Superior Parietal |
| Left SupraMarginal | Left Inferior Parietal |
| Left SupraMarginal | Right ParaCentral Lobule |
| Left SupraMarginal | Right SupraMarginal |
| Left SupraMarginal | Right Superior Parietal |
| Left SupraMarginal | Right Inferior Parietal |
| Left Superior Parietal | Left Inferior Parietal |
| Left Superior Parietal | Right ParaCentral Lobule |
| Left Superior Parietal | Right PostCentral |
| Left Superior Parietal | Right SupraMarginal |
| Left Superior Parietal | Right Superior Parietal |
| Left Superior Parietal | Right Inferior Parietal |
| Left Inferior Parietal | Right ParaCentral Lobule |
| Left Inferior Parietal | Right SupraMarginal |
| Left Inferior Parietal | Right Superior Parietal |
| Left Inferior Parietal | Right Inferior Parietal |
| Right ParaCentral Lobule | Right PostCentral |
| Right ParaCentral Lobule | Right SupraMarginal |
| Right ParaCentral Lobule | Right Superior Parietal |

| Right ParaCentral Lobule | Right Inferior Parietal |
| --- | --- |
| Right Posterior Cingulate | Right Isthmus |
| Right PostCentral | Right SupraMarginal |
| Right PostCentral | Right Superior Parietal |
| Right SupraMarginal | Right Superior Parietal |
| Right SupraMarginal | Right Inferior Parietal |
| Right Superior Parietal | Right Inferior Parietal |
| Left ParaCentral Lobule | Left PreCuneus |
| Left ParaCentral Lobule | Right PreCuneus |
| Left ParaCentral Lobule | Right Pericalcarine |
| Left PostCentral | Left PreCuneus |
| Left PostCentral | Left Lingual |
| Left PostCentral | Left Lateral Occipital |
| Left PostCentral | Right PreCuneus |
| Left PostCentral | Right Lateral Occipital |
| Left SupraMarginal | Left PreCuneus |
| Left SupraMarginal | Left Lateral Occipital |
| Left SupraMarginal | Right PreCuneus |
| Left SupraMarginal | Right Lateral Occipital |
| Left Superior Parietal | Left PreCuneus |
| Left Superior Parietal | Left Lingual |
| Left Superior Parietal | Left Cuneus |
| Left Superior Parietal | Left Lateral Occipital |
| Left Superior Parietal | Right PreCuneus |
| Left Superior Parietal | Right Lingual |
| Left Superior Parietal | Right Cuneus |
| Left Superior Parietal | Right Lateral Occipital |
| Left Inferior Parietal | Left PreCuneus |
| Left Inferior Parietal | Left Lingual |
| Left Inferior Parietal | Left Lateral Occipital |
| Left Inferior Parietal | Right PreCuneus |
| Left Inferior Parietal | Right Lateral Occipital |
| Right ParaCentral Lobule | Right PreCuneus |
| Right ParaCentral Lobule | Right Lateral Occipital |
| Right SupraMarginal | Right PreCuneus |
| Right SupraMarginal | Right Lateral Occipital |
| Right Superior Parietal | Right PreCuneus |
| Right Superior Parietal | Right Lingual |
| Right Superior Parietal | Right Pericalcarine |
| Right Superior Parietal | Right Cuneus |
| Right Superior Parietal | Right Lateral Occipital |
| Right Inferior Parietal | Right PreCuneus |
| Right Inferior Parietal | Right Lingual |
| Right Inferior Parietal | Right Lateral Occipital |
| Left PostCentral | Right Insula |
| Left SupraMarginal | Right Insula |
| Left Superior Parietal | Right Insula |
| Left Inferior Parietal | Right Insula |
| Left PreCuneus | Right ParaCentral Lobule |
| Left PreCuneus | Right SupraMarginal |
| Left PreCuneus | Right Superior Parietal |
| Left PreCuneus | Right Inferior Parietal |
| Left Lingual | Right ParaCentral Lobule |
| Left Lingual | Right PostCentral |
| Left Lingual | Right SupraMarginal |
| Left Lingual | Right Superior Parietal |
| Left Lingual | Right Inferior Parietal |
| Left Cuneus | Right Superior Parietal |
| Left Cuneus | Right Inferior Parietal |
| Left Lateral Occipital | Right ParaCentral Lobule |
| Left Lateral Occipital | Right SupraMarginal |
| Left Lateral Occipital | Right Superior Parietal |
| Left Lateral Occipital | Right Inferior Parietal |
| Left Insula | Left Inferior Parietal |
| Left Insula | Right ParaCentral Lobule |
| Left Insula | Right Inferior Parietal |

| Right Insula | Right PostCentral |
| --- | --- |
| Right Insula | Right SupraMarginal |
| Right Insula | Right Superior Parietal |
| Right Insula | Right Inferior Parietal |
| Left PreCuneus | Left Lingual |
| Left PreCuneus | Left Lateral Occipital |
| Left PreCuneus | Right PreCuneus |
| Left PreCuneus | Right Lingual |
| Left PreCuneus | Right Lateral Occipital |
| Left Lingual | Left Lateral Occipital |
| Left Lingual | Right PreCuneus |
| Left Lingual | Right Lingual |
| Left Pericalcarine | Right Pericalcarine |
| Left Cuneus | Right Pericalcarine |
| Left Cuneus | Right Cuneus |
| Left Lateral Occipital | Right PreCuneus |
| Left Lateral Occipital | Right Lingual |
| Left Lateral Occipital | Right Lateral Occipital |
| Right PreCuneus | Right Lingual |
| Right Lingual | Right Lateral Occipital |
| Right Pericalcarine | Right Cuneus |
| Right Cuneus | Right Lateral Occipital |
| Left PreCuneus | Right Insula |
| Left Lateral Occipital | Right Insula |
| Left Insula | Left Lateral Occipital |
| Left Insula | Right PreCuneus |
| Left Insula | Right Lateral Occipital |
| Right Insula | Right PreCuneus |
| Right Insula | Right Lateral Occipital |
| Left Insula | Right Insula |

**Table S18.** Cross-cortical correlations in the adolescence-onset conduct disorder (AO-CD) group showing overlap between the significant inter-regional correlations identified using structural covariance methods in the Cambridge and Southampton samples when including the covariates in the statistical models

| Left Superior Frontal | Right Superior Frontal |
| --- | --- |
| Left Superior Frontal | Right Caudal Middle Frontal |
| Left Caudal Middle Frontal | Left PreCentral |
| Left Caudal Middle Frontal | Right Superior Frontal |
| Left Caudal Middle Frontal | Right Caudal Middle Frontal |
| Left ParsOrbitalis | Left Orbital Frontal |
| Left Orbital Frontal | Right Orbital Frontal |
| Left PreCentral | Right PreCentral |
| Right Superior Frontal | Right Rostral Middle Frontal |
| Right Superior Frontal | Right Caudal Middle Frontal |
| Right Rostral Middle Frontal | Right Caudal Middle Frontal |
| Left Superior Frontal | Right Fusiform |
| Left Caudal Middle Frontal | Right Fusiform |
| Left PreCentral | Left Middle Temporal |
| Left Superior Frontal | Left PostCentral |
| Left Superior Frontal | Left SupraMarginal |
| Left Superior Frontal | Left Inferior Parietal |
| Left Superior Frontal | Right SupraMarginal |
| Left Superior Frontal | Right Superior Parietal |
| Left Superior Frontal | Right Inferior Parietal |
| Left Caudal Middle Frontal | Left SupraMarginal |
| Right Superior Frontal | Right SupraMarginal |
| Right Superior Frontal | Right Superior Parietal |
| Right Superior Frontal | Right Inferior Parietal |
| Right Caudal Middle Frontal | Right SupraMarginal |
| Right PreCentral | Right SupraMarginal |
| Left Superior Frontal | Left PreCuneus |
| Left Superior Frontal | Left Lateral Occipital |
| Left Caudal Middle Frontal | Left PreCuneus |
| Left PreCentral | Left Lateral Occipital |

| Left PreCentral | Right Lingual |
| --- | --- |
| Left Transverse Temporal | Right Caudal Middle Frontal |
| Left PostCentral | Right Caudal Middle Frontal |
| Left SupraMarginal | Right Superior Frontal |
| Left SupraMarginal | Right Caudal Middle Frontal |
| Left Inferior Parietal | Right Caudal Middle Frontal |
| Left PreCuneus | Right Superior Frontal |
| Left Insula | Right Orbital Frontal |
| Left Fusiform | Right Fusiform |
| Left Fusiform | Right Inferior Temporal |
| Right Fusiform | Right Inferior Temporal |
| Left Middle Temporal | Left SupraMarginal |
| Left Transverse Temporal | Left SupraMarginal |
| Left Banks Superior Temporal | Right ParaCentral Lobule |
| Right Fusiform | Right SupraMarginal |
| Right Fusiform | Right Superior Parietal |
| Right Fusiform | Right PreCuneus |
| Right Fusiform | Right Lateral Occipital |
| Left Transverse Temporal | Right Insula |
| Left SupraMarginal | Right Superior Temporal |
| Left Inferior Parietal | Right Middle Temporal |
| Right ParaCentral Lobule | Right Fusiform |
| Left PreCuneus | Right Fusiform |
| Left Lateral Occipital | Right Fusiform |
| Left Insula | Right Superior Temporal |
| Left ParaCentral Lobule | Left SupraMarginal |
| Left ParaCentral Lobule | Left Superior Parietal |
| Left ParaCentral Lobule | Right Superior Parietal |
| Left PostCentral | Left Superior Parietal |
| Left PostCentral | Right ParaCentral Lobule |
| Left PostCentral | Right SupraMarginal |
| Left PostCentral | Right Superior Parietal |
| Left SupraMarginal | Left Superior Parietal |
| Left SupraMarginal | Right SupraMarginal |
| Left SupraMarginal | Right Inferior Parietal |
| Left Superior Parietal | Left Inferior Parietal |
| Left Superior Parietal | Right Superior Parietal |
| Left Inferior Parietal | Right SupraMarginal |
| Left Inferior Parietal | Right Superior Parietal |
| Left Inferior Parietal | Right Inferior Parietal |
| Right ParaCentral Lobule | Right Superior Parietal |
| Right SupraMarginal | Right Inferior Parietal |
| Right Superior Parietal | Right Inferior Parietal |
| Left ParaCentral Lobule | Left PreCuneus |
| Left PostCentral | Left PreCuneus |
| Left PostCentral | Left Lateral Occipital |
| Left PostCentral | Right Lateral Occipital |
| Left SupraMarginal | Left PreCuneus |
| Left SupraMarginal | Right PreCuneus |
| Left Superior Parietal | Left PreCuneus |
| Left Superior Parietal | Right PreCuneus |
| Left Inferior Parietal | Left PreCuneus |
| Left Inferior Parietal | Right PreCuneus |
| Right ParaCentral Lobule | Right Lingual |
| Right ParaCentral Lobule | Right Lateral Occipital |
| Right PostCentral | Right Lateral Occipital |
| Right Superior Parietal | Right PreCuneus |
| Right Inferior Parietal | Right PreCuneus |
| Left PreCuneus | Right ParaCentral Lobule |
| Left PreCuneus | Right SupraMarginal |
| Left PreCuneus | Right Superior Parietal |
| Left PreCuneus | Right Inferior Parietal |
| Left Lateral Occipital | Right ParaCentral Lobule |
| Left Lateral Occipital | Right Superior Parietal |
| Left Lateral Occipital | Right Inferior Parietal |
| Left PreCuneus | Left Lateral Occipital |

| Left PreCuneus | Right PreCuneus |
| --- | --- |
| Left Lingual | Left Lateral Occipital |
| Left Lingual | Right Lingual |
| Left Cuneus | Left Lateral Occipital |
| Left Cuneus | Right Lingual |
| Left Cuneus | Right Cuneus |
| Left Cuneus | Right Lateral Occipital |
| Left Lateral Occipital | Right PreCuneus |
| Left Lateral Occipital | Right Lingual |
| Left Lateral Occipital | Right Lateral Occipital |
| Right PreCuneus | Right Lateral Occipital |
| Right Lingual | Right Cuneus |
| Right Lingual | Right Lateral Occipital |

**Table S19.** Cross-cortical correlations in the healthy controls group showing overlap between the significant inter-regional correlations identified using structural covariance methods in the Cambridge and Southampton samples *when* *not* including the covariates in the statistical models

| Left Superior Frontal | Left Rostral Middle Frontal |
| --- | --- |
| Left Superior Frontal | Left Caudal Middle Frontal |
| Left Superior Frontal | Left ParsOpercularis |
| Left Superior Frontal | Left Orbital Frontal |
| Left Superior Frontal | Left Medial Orbital Frontal |
| Left Superior Frontal | Right Superior Frontal |
| Left Superior Frontal | Right Rostral Middle Frontal |
| Left Superior Frontal | Right Caudal Middle Frontal |
| Left Superior Frontal | Right ParsOpercularis |
| Left Superior Frontal | Right ParsOrbitalis |
| Left Superior Frontal | Right Orbital Frontal |
| Left Superior Frontal | Right Medial Orbital Frontal |
| Left Rostral Middle Frontal | Left Caudal Middle Frontal |
| Left Rostral Middle Frontal | Left ParsTriangularis |
| Left Rostral Middle Frontal | Left ParsOrbitalis |
| Left Rostral Middle Frontal | Right Superior Frontal |
| Left Rostral Middle Frontal | Right Rostral Middle Frontal |
| Left Rostral Middle Frontal | Right Caudal Middle Frontal |
| Left Rostral Middle Frontal | Right ParsOpercularis |
| Left Rostral Middle Frontal | Right ParsTriangularis |
| Left Caudal Middle Frontal | Left Orbital Frontal |
| Left Caudal Middle Frontal | Right Superior Frontal |
| Left Caudal Middle Frontal | Right Rostral Middle Frontal |
| Left Caudal Middle Frontal | Right Caudal Middle Frontal |
| Left Caudal Middle Frontal | Right ParsOpercularis |
| Left ParsOpercularis | Right Superior Frontal |
| Left ParsOpercularis | Right Orbital Frontal |
| Left ParsOrbitalis | Right Superior Frontal |
| Left ParsOrbitalis | Right Rostral Middle Frontal |
| Left ParsOrbitalis | Right PreCentral |
| Left Orbital Frontal | Right Superior Frontal |
| Left Orbital Frontal | Right Rostral Middle Frontal |
| Left Orbital Frontal | Right Caudal Middle Frontal |
| Left Orbital Frontal | Right ParsOpercularis |
| Left Orbital Frontal | Right ParsOrbitalis |
| Left Orbital Frontal | Right Orbital Frontal |
| Left Orbital Frontal | Right Medial Orbital Frontal |
| Left Medial Orbital Frontal | Right Rostral Middle Frontal |
| Left Medial Orbital Frontal | Right Orbital Frontal |
| Left Medial Orbital Frontal | Right Medial Orbital Frontal |
| Left PreCentral | Right PreCentral |
| Right Superior Frontal | Right Rostral Middle Frontal |
| Right Superior Frontal | Right Caudal Middle Frontal |
| Right Superior Frontal | Right ParsOpercularis |
| Right Superior Frontal | Right ParsTriangularis |
| Right Superior Frontal | Right ParsOrbitalis |
| Right Superior Frontal | Right Orbital Frontal |
| Right Rostral Middle Frontal | Right Caudal Middle Frontal |

| Right Rostral Middle Frontal | Right ParsOpercularis |
| --- | --- |
| Right Rostral Middle Frontal | Right ParsTriangularis |
| Right Rostral Middle Frontal | Right ParsOrbitalis |
| Right Rostral Middle Frontal | Right Orbital Frontal |
| Right Caudal Middle Frontal | Right ParsOpercularis |
| Right Caudal Middle Frontal | Right ParsTriangularis |
| Right Caudal Middle Frontal | Right Orbital Frontal |
| Right Caudal Middle Frontal | Right PreCentral |
| Right ParsOpercularis | Right ParsTriangularis |
| Right ParsOpercularis | Right Orbital Frontal |
| Right ParsOpercularis | Right PreCentral |
| Right ParsOrbitalis | Right Orbital Frontal |
| Right ParsOrbitalis | Right Medial Orbital Frontal |
| Right Orbital Frontal | Right Medial Orbital Frontal |
| Right Medial Orbital Frontal | Right RA cingulate |
| Right Medial Orbital Frontal | Right CA Cingulate |
| Left ParsOpercularis | Left Middle Temporal |
| Left ParsTriangularis | Left Middle Temporal |
| Left ParsOrbitalis | Left Middle Temporal |
| Left ParsOrbitalis | Left Inferior Temporal |
| Left ParsOrbitalis | Right Middle Temporal |
| Right Rostral Middle Frontal | Right Middle Temporal |
| Right Caudal Middle Frontal | Right Fusiform |
| Right Caudal Middle Frontal | Right Middle Temporal |
| Right Caudal Middle Frontal | Right Inferior Temporal |
| Right Orbital Frontal | Right Fusiform |
| Right PreCentral | Right Superior Temporal |
| Left Superior Frontal | Left SupraMarginal |
| Left Superior Frontal | Left Superior Parietal |
| Left Superior Frontal | Left Inferior Parietal |
| Left Superior Frontal | Right ParaCentral Lobule |
| Left Superior Frontal | Right Superior Parietal |
| Left Superior Frontal | Right Inferior Parietal |
| Left Rostral Middle Frontal | Left SupraMarginal |
| Left Rostral Middle Frontal | Left Inferior Parietal |
| Left Caudal Middle Frontal | Left PostCentral |
| Left Caudal Middle Frontal | Left SupraMarginal |
| Left Caudal Middle Frontal | Left Superior Parietal |
| Left Caudal Middle Frontal | Left Inferior Parietal |
| Left Caudal Middle Frontal | Right ParaCentral Lobule |
| Left Caudal Middle Frontal | Right Superior Parietal |
| Left Orbital Frontal | Right SupraMarginal |
| Left PreCentral | Left PostCentral |
| Left PreCentral | Left SupraMarginal |
| Right Superior Frontal | Right ParaCentral Lobule |
| Right Superior Frontal | Right Superior Parietal |
| Right Superior Frontal | Right Inferior Parietal |
| Right Rostral Middle Frontal | Right Inferior Parietal |
| Right Caudal Middle Frontal | Right ParaCentral Lobule |
| Right Caudal Middle Frontal | Right Inferior Parietal |
| Right Orbital Frontal | Right SupraMarginal |
| Right Orbital Frontal | Right Inferior Parietal |
| Left Superior Frontal | Left PreCuneus |
| Left Superior Frontal | Left Cuneus |
| Left Superior Frontal | Right PreCuneus |
| Left Orbital Frontal | Left PreCuneus |
| Left Orbital Frontal | Right PreCuneus |
| Left Orbital Frontal | Right Lateral Occipital |
| Right Superior Frontal | Right PreCuneus |
| Right Rostral Middle Frontal | Right PreCuneus |
| Right Caudal Middle Frontal | Right PreCuneus |
| Right ParsOpercularis | Right PreCuneus |
| Right ParsTriangularis | Right PreCuneus |
| Right ParsOrbitalis | Right PreCuneus |
| Right Orbital Frontal | Right PreCuneus |
| Right Medial Orbital Frontal | Right PreCuneus |

| Right Orbital Frontal | Right Insula |
| --- | --- |
| Right Medial Orbital Frontal | Right Insula |
| Left Middle Temporal | Right Superior Frontal |
| Left Middle Temporal | Right Rostral Middle Frontal |
| Left Middle Temporal | Right Caudal Middle Frontal |
| Left Middle Temporal | Right ParsOpercularis |
| Left Middle Temporal | Right ParsTriangularis |
| Left Middle Temporal | Right Orbital Frontal |
| Left Inferior Temporal | Right Rostral Middle Frontal |
| Left Inferior Temporal | Right Caudal Middle Frontal |
| Left ParaCentral Lobule | Right Caudal Middle Frontal |
| Left PostCentral | Right Caudal Middle Frontal |
| Left SupraMarginal | Right Rostral Middle Frontal |
| Left SupraMarginal | Right Caudal Middle Frontal |
| Left SupraMarginal | Right ParsOpercularis |
| Left Superior Parietal | Right Superior Frontal |
| Left Superior Parietal | Right ParsOpercularis |
| Left Inferior Parietal | Right Superior Frontal |
| Left Inferior Parietal | Right Rostral Middle Frontal |
| Left Inferior Parietal | Right Caudal Middle Frontal |
| Left Inferior Parietal | Right ParsTriangularis |
| Left PreCuneus | Right Caudal Middle Frontal |
| Left PreCuneus | Right ParsOpercularis |
| Left Parahippocampal | Right Parahippocampal |
| Left Fusiform | Left Superior Temporal |
| Left Fusiform | Left Middle Temporal |
| Left Fusiform | Right Fusiform |
| Left Fusiform | Right Inferior Temporal |
| Left Superior Temporal | Left Middle Temporal |
| Left Superior Temporal | Left Inferior Temporal |
| Left Superior Temporal | Left Banks Superior Temporal |
| Left Superior Temporal | Right Superior Temporal |
| Left Superior Temporal | Right Inferior Temporal |
| Left Middle Temporal | Left Inferior Temporal |
| Left Middle Temporal | Right Fusiform |
| Left Middle Temporal | Right Superior Temporal |
| Left Middle Temporal | Right Middle Temporal |
| Left Middle Temporal | Right Inferior Temporal |
| Left Inferior Temporal | Right Fusiform |
| Left Inferior Temporal | Right Superior Temporal |
| Left Inferior Temporal | Right Middle Temporal |
| Left Inferior Temporal | Right Inferior Temporal |
| Left Banks Superior Temporal | Right Banks Superior Temporal |
| Right Fusiform | Right Superior Temporal |
| Right Fusiform | Right Middle Temporal |
| Right Superior Temporal | Right Middle Temporal |
| Right Superior Temporal | Right Inferior Temporal |
| Right Middle Temporal | Right Inferior Temporal |
| Left Fusiform | Right Inferior Parietal |
| Left Superior Temporal | Right SupraMarginal |
| Left Superior Temporal | Right Inferior Parietal |
| Left Middle Temporal | Left PostCentral |
| Left Middle Temporal | Left SupraMarginal |
| Left Middle Temporal | Left Inferior Parietal |
| Left Inferior Temporal | Left Inferior Parietal |
| Left Banks Superior Temporal | Left Inferior Parietal |
| Right Fusiform | Right PreCuneus |
| Right Fusiform | Right Lateral Occipital |
| Left Fusiform | Right Insula |
| Left PostCentral | Right Superior Temporal |
| Left SupraMarginal | Right Superior Temporal |
| Right ParaCentral Lobule | Right Superior Temporal |
| Right ParaCentral Lobule | Right Middle Temporal |
| Left Lateral Occipital | Right Inferior Temporal |
| Left Insula | Right Inferior Temporal |
| Right Insula | Right Fusiform |

| Left ParaCentral Lobule | Left PostCentral |
| --- | --- |
| Left ParaCentral Lobule | Right ParaCentral Lobule |
| Left ParaCentral Lobule | Right Inferior Parietal |
| Left PostCentral | Left SupraMarginal |
| Left PostCentral | Right ParaCentral Lobule |
| Left PostCentral | Right SupraMarginal |
| Left PostCentral | Right Superior Parietal |
| Left SupraMarginal | Left Superior Parietal |
| Left SupraMarginal | Left Inferior Parietal |
| Left SupraMarginal | Right Superior Parietal |
| Left Superior Parietal | Left Inferior Parietal |
| Left Superior Parietal | Right Superior Parietal |
| Left Superior Parietal | Right Inferior Parietal |
| Left Inferior Parietal | Right ParaCentral Lobule |
| Left Inferior Parietal | Right Superior Parietal |
| Left Inferior Parietal | Right Inferior Parietal |
| Right ParaCentral Lobule | Right SupraMarginal |
| Right ParaCentral Lobule | Right Inferior Parietal |
| Right Superior Parietal | Right Inferior Parietal |
| Left ParaCentral Lobule | Left Lateral Occipital |
| Left ParaCentral Lobule | Right Lateral Occipital |
| Left PostCentral | Left PreCuneus |
| Left PostCentral | Right Lateral Occipital |
| Left SupraMarginal | Left PreCuneus |
| Left SupraMarginal | Right PreCuneus |
| Left Superior Parietal | Left PreCuneus |
| Left Superior Parietal | Left Lateral Occipital |
| Left Superior Parietal | Right PreCuneus |
| Left Inferior Parietal | Left Lateral Occipital |
| Left Inferior Parietal | Right PreCuneus |
| Right PostCentral | Right PreCuneus |
| Right Superior Parietal | Right PreCuneus |
| Right Superior Parietal | Right Lateral Occipital |
| Right Inferior Parietal | Right PreCuneus |
| Left PreCuneus | Right Superior Parietal |
| Left PreCuneus | Right Inferior Parietal |
| Left Lingual | Right Isthmus |
| Left Cuneus | Right Superior Parietal |
| Left Lateral Occipital | Right Superior Parietal |
| Left Lateral Occipital | Right Inferior Parietal |
| Left PreCuneus | Left Cuneus |
| Left PreCuneus | Right PreCuneus |
| Left PreCuneus | Right Cuneus |
| Left PreCuneus | Right Lateral Occipital |
| Left Lingual | Right Lingual |
| Left Pericalcarine | Left Cuneus |
| Left Cuneus | Right PreCuneus |
| Left Cuneus | Right Cuneus |
| Left Lateral Occipital | Right PreCuneus |
| Left Lateral Occipital | Right Lateral Occipital |
| Right PreCuneus | Right Cuneus |
| Right PreCuneus | Right Lateral Occipital |
| Right Insula | Right PreCuneus |

**Table S20.** Cross-cortical correlations in the childhood-onset conduct disorder (CO-CD) group showing overlap between the significant inter-regional correlations identified using structural covariance methods in the Cambridge and Southampton samples *when not* including the covariates in the statistical models

| Left Superior Frontal | Left Rostral Middle Frontal |
| --- | --- |
| Left Superior Frontal | Left Caudal Middle Frontal |
| Left Superior Frontal | Left ParsOpercularis |
| Left Superior Frontal | Left ParsTriangularis |
| Left Superior Frontal | Left Orbital Frontal |
| Left Superior Frontal | Left PreCentral |
| Left Superior Frontal | Right Superior Frontal |
| Left Superior Frontal | Right Rostral Middle Frontal |
| Left Superior Frontal | Right Caudal Middle Frontal |
| Left Superior Frontal | Right ParsOpercularis |
| Left Superior Frontal | Right ParsTriangularis |
| Left Superior Frontal | Right Orbital Frontal |
| Left Superior Frontal | Right PreCentral |
| Left Superior Frontal | Right CA Cingulate |
| Left Rostral Middle Frontal | Left Caudal Middle Frontal |
| Left Rostral Middle Frontal | Left ParsOpercularis |
| Left Rostral Middle Frontal | Left ParsTriangularis |
| Left Rostral Middle Frontal | Right Superior Frontal |
| Left Rostral Middle Frontal | Right Rostral Middle Frontal |
| Left Rostral Middle Frontal | Right ParsOpercularis |
| Left Caudal Middle Frontal | Left ParsOpercularis |
| Left Caudal Middle Frontal | Left ParsTriangularis |
| Left Caudal Middle Frontal | Left Orbital Frontal |
| Left Caudal Middle Frontal | Left PreCentral |
| Left Caudal Middle Frontal | Right Superior Frontal |
| Left Caudal Middle Frontal | Right Rostral Middle Frontal |
| Left Caudal Middle Frontal | Right Caudal Middle Frontal |
| Left Caudal Middle Frontal | Right ParsOpercularis |
| Left Caudal Middle Frontal | Right ParsTriangularis |
| Left Caudal Middle Frontal | Right PreCentral |
| Left ParsOpercularis | Left ParsTriangularis |
| Left ParsOpercularis | Left Orbital Frontal |
| Left ParsOpercularis | Left PreCentral |
| Left ParsOpercularis | Right Superior Frontal |
| Left ParsOpercularis | Right Rostral Middle Frontal |
| Left ParsOpercularis | Right Caudal Middle Frontal |
| Left ParsOpercularis | Right ParsOpercularis |
| Left ParsOpercularis | Right ParsTriangularis |
| Left ParsOpercularis | Right PreCentral |
| Left ParsTriangularis | Left Orbital Frontal |
| Left ParsTriangularis | Left PreCentral |
| Left ParsTriangularis | Left CA Cingulate |
| Left ParsTriangularis | Right Superior Frontal |
| Left ParsTriangularis | Right Caudal Middle Frontal |
| Left ParsTriangularis | Right ParsOpercularis |
| Left ParsTriangularis | Right ParsTriangularis |
| Left ParsTriangularis | Right Orbital Frontal |
| Left ParsTriangularis | Right PreCentral |
| Left ParsTriangularis | Right CA Cingulate |
| Left ParsOrbitalis | Left Orbital Frontal |
| Left Orbital Frontal | Left Frontal Pole |
| Left Orbital Frontal | Left PreCentral |
| Left Orbital Frontal | Right Superior Frontal |
| Left Orbital Frontal | Right Caudal Middle Frontal |
| Left Orbital Frontal | Right ParsOpercularis |
| Left Orbital Frontal | Right ParsTriangularis |
| Left Orbital Frontal | Right Orbital Frontal |
| Left Orbital Frontal | Right PreCentral |
| Left Orbital Frontal | Right CA Cingulate |
| Left Medial Orbital Frontal | Right Rostral Middle Frontal |
| Left Frontal Pole | Right Caudal Middle Frontal |
| Left Frontal Pole | Right ParsTriangularis |

| Left Frontal Pole | Right CA Cingulate |
| --- | --- |
| Left PreCentral | Right Superior Frontal |
| Left PreCentral | Right Caudal Middle Frontal |
| Left PreCentral | Right ParsOpercularis |
| Left PreCentral | Right ParsTriangularis |
| Left PreCentral | Right PreCentral |
| Left CA Cingulate | Right ParsOpercularis |
| Left CA Cingulate | Right CA Cingulate |
| Right Superior Frontal | Right Rostral Middle Frontal |
| Right Superior Frontal | Right Caudal Middle Frontal |
| Right Superior Frontal | Right ParsOpercularis |
| Right Superior Frontal | Right ParsTriangularis |
| Right Superior Frontal | Right Orbital Frontal |
| Right Superior Frontal | Right Medial Orbital Frontal |
| Right Superior Frontal | Right PreCentral |
| Right Rostral Middle Frontal | Right Caudal Middle Frontal |
| Right Rostral Middle Frontal | Right ParsOpercularis |
| Right Rostral Middle Frontal | Right ParsTriangularis |
| Right Caudal Middle Frontal | Right ParsOpercularis |
| Right Caudal Middle Frontal | Right ParsTriangularis |
| Right Caudal Middle Frontal | Right Orbital Frontal |
| Right Caudal Middle Frontal | Right PreCentral |
| Right Caudal Middle Frontal | Right CA Cingulate |
| Right ParsOpercularis | Right ParsTriangularis |
| Right ParsOpercularis | Right Orbital Frontal |
| Right ParsOpercularis | Right PreCentral |
| Right ParsTriangularis | Right ParsOrbitalis |
| Right ParsTriangularis | Right Orbital Frontal |
| Right ParsTriangularis | Right Medial Orbital Frontal |
| Right ParsTriangularis | Right PreCentral |
| Right ParsOrbitalis | Right Orbital Frontal |
| Right Orbital Frontal | Right Medial Orbital Frontal |
| Left Superior Frontal | Left Fusiform |
| Left Superior Frontal | Left Superior Temporal |
| Left Superior Frontal | Left Middle Temporal |
| Left Superior Frontal | Left Inferior Temporal |
| Left Superior Frontal | Right Temporal Pole |
| Left Superior Frontal | Right Superior Temporal |
| Left Superior Frontal | Right Middle Temporal |
| Left Superior Frontal | Right Inferior Temporal |
| Left Superior Frontal | Right Transverse Temporal |
| Left Rostral Middle Frontal | Left Superior Temporal |
| Left Rostral Middle Frontal | Right Superior Temporal |
| Left Rostral Middle Frontal | Right Inferior Temporal |
| Left Caudal Middle Frontal | Left Superior Temporal |
| Left Caudal Middle Frontal | Left Middle Temporal |
| Left Caudal Middle Frontal | Left Inferior Temporal |
| Left Caudal Middle Frontal | Right Superior Temporal |
| Left Caudal Middle Frontal | Right Middle Temporal |
| Left Caudal Middle Frontal | Right Inferior Temporal |
| Left ParsOpercularis | Left Fusiform |
| Left ParsOpercularis | Left Superior Temporal |
| Left ParsOpercularis | Left Middle Temporal |
| Left ParsOpercularis | Left Inferior Temporal |
| Left ParsOpercularis | Left Transverse Temporal |
| Left ParsOpercularis | Right Superior Temporal |
| Left ParsOpercularis | Right Middle Temporal |
| Left ParsOpercularis | Right Inferior Temporal |
| Left ParsTriangularis | Left Fusiform |
| Left ParsTriangularis | Left Superior Temporal |
| Left ParsTriangularis | Left Middle Temporal |
| Left ParsTriangularis | Left Inferior Temporal |
| Left ParsTriangularis | Right Fusiform |
| Left ParsTriangularis | Right Superior Temporal |
| Left ParsTriangularis | Right Middle Temporal |
| Left ParsTriangularis | Right Banks Superior Temporal |

| Left ParsOrbitalis | Left Inferior Temporal |
| --- | --- |
| Left ParsOrbitalis | Right Temporal Pole |
| Left Orbital Frontal | Left Temporal Pole |
| Left Orbital Frontal | Left Fusiform |
| Left Orbital Frontal | Left Superior Temporal |
| Left Orbital Frontal | Left Middle Temporal |
| Left Orbital Frontal | Left Inferior Temporal |
| Left Orbital Frontal | Right Temporal Pole |
| Left Orbital Frontal | Right Superior Temporal |
| Left Orbital Frontal | Right Middle Temporal |
| Left Orbital Frontal | Right Inferior Temporal |
| Left PreCentral | Left Superior Temporal |
| Left PreCentral | Left Middle Temporal |
| Left PreCentral | Right Entorhinal |
| Left PreCentral | Right Superior Temporal |
| Left PreCentral | Right Middle Temporal |
| Left PreCentral | Right Inferior Temporal |
| Right Superior Frontal | Right Temporal Pole |
| Right Superior Frontal | Right Fusiform |
| Right Superior Frontal | Right Superior Temporal |
| Right Superior Frontal | Right Middle Temporal |
| Right Superior Frontal | Right Inferior Temporal |
| Right Rostral Middle Frontal | Right Superior Temporal |
| Right Caudal Middle Frontal | Right Superior Temporal |
| Right Caudal Middle Frontal | Right Middle Temporal |
| Right ParsOpercularis | Right Middle Temporal |
| Right ParsOpercularis | Right Inferior Temporal |
| Right ParsTriangularis | Right Entorhinal |
| Right ParsTriangularis | Right Parahippocampal |
| Right ParsTriangularis | Right Superior Temporal |
| Right ParsTriangularis | Right Middle Temporal |
| Right ParsTriangularis | Right Inferior Temporal |
| Right ParsTriangularis | Right Banks Superior Temporal |
| Right Orbital Frontal | Right Entorhinal |
| Right Orbital Frontal | Right Parahippocampal |
| Right Orbital Frontal | Right Temporal Pole |
| Right Orbital Frontal | Right Superior Temporal |
| Right Orbital Frontal | Right Middle Temporal |
| Right Orbital Frontal | Right Inferior Temporal |
| Right Orbital Frontal | Right Transverse Temporal |
| Right PreCentral | Right Entorhinal |
| Right PreCentral | Right Parahippocampal |
| Right PreCentral | Right Temporal Pole |
| Right PreCentral | Right Superior Temporal |
| Right PreCentral | Right Middle Temporal |
| Right PreCentral | Right Inferior Temporal |
| Right PreCentral | Right Transverse Temporal |
| Right PreCentral | Right Banks Superior Temporal |
| Left Superior Frontal | Left ParaCentral Lobule |
| Left Superior Frontal | Left PostCentral |
| Left Superior Frontal | Left SupraMarginal |
| Left Superior Frontal | Left Superior Parietal |
| Left Superior Frontal | Left Inferior Parietal |
| Left Superior Frontal | Right ParaCentral Lobule |
| Left Superior Frontal | Right SupraMarginal |
| Left Superior Frontal | Right Superior Parietal |
| Left Superior Frontal | Right Inferior Parietal |
| Left Rostral Middle Frontal | Left Superior Parietal |
| Left Rostral Middle Frontal | Right SupraMarginal |
| Left Caudal Middle Frontal | Left ParaCentral Lobule |
| Left Caudal Middle Frontal | Left PostCentral |
| Left Caudal Middle Frontal | Left SupraMarginal |
| Left Caudal Middle Frontal | Left Superior Parietal |
| Left Caudal Middle Frontal | Left Inferior Parietal |
| Left Caudal Middle Frontal | Right ParaCentral Lobule |
| Left Caudal Middle Frontal | Right SupraMarginal |

| Left Caudal Middle Frontal | Right Superior Parietal |
| --- | --- |
| Left Caudal Middle Frontal | Right Inferior Parietal |
| Left ParsOpercularis | Left ParaCentral Lobule |
| Left ParsOpercularis | Left PostCentral |
| Left ParsOpercularis | Left SupraMarginal |
| Left ParsOpercularis | Left Superior Parietal |
| Left ParsOpercularis | Left Inferior Parietal |
| Left ParsOpercularis | Right ParaCentral Lobule |
| Left ParsOpercularis | Right SupraMarginal |
| Left ParsOpercularis | Right Inferior Parietal |
| Left ParsTriangularis | Left ParaCentral Lobule |
| Left ParsTriangularis | Left SupraMarginal |
| Left ParsTriangularis | Left Superior Parietal |
| Left ParsTriangularis | Left Inferior Parietal |
| Left ParsTriangularis | Right ParaCentral Lobule |
| Left ParsTriangularis | Right SupraMarginal |
| Left ParsTriangularis | Right Superior Parietal |
| Left ParsTriangularis | Right Inferior Parietal |
| Left Orbital Frontal | Left ParaCentral Lobule |
| Left Orbital Frontal | Left SupraMarginal |
| Left Orbital Frontal | Left Superior Parietal |
| Left Orbital Frontal | Left Inferior Parietal |
| Left Orbital Frontal | Right Posterior Cingulate |
| Left Orbital Frontal | Right SupraMarginal |
| Left Orbital Frontal | Right Inferior Parietal |
| Left Medial Orbital Frontal | Left Inferior Parietal |
| Left Medial Orbital Frontal | Right Inferior Parietal |
| Left Frontal Pole | Right SupraMarginal |
| Left PreCentral | Left ParaCentral Lobule |
| Left PreCentral | Left PostCentral |
| Left PreCentral | Left SupraMarginal |
| Left PreCentral | Left Superior Parietal |
| Left PreCentral | Right ParaCentral Lobule |
| Left PreCentral | Right SupraMarginal |
| Left PreCentral | Right Inferior Parietal |
| Right Superior Frontal | Right ParaCentral Lobule |
| Right Superior Frontal | Right PostCentral |
| Right Superior Frontal | Right SupraMarginal |
| Right Superior Frontal | Right Superior Parietal |
| Right Superior Frontal | Right Inferior Parietal |
| Right Rostral Middle Frontal | Right ParaCentral Lobule |
| Right Rostral Middle Frontal | Right PostCentral |
| Right Rostral Middle Frontal | Right SupraMarginal |
| Right Caudal Middle Frontal | Right ParaCentral Lobule |
| Right Caudal Middle Frontal | Right PostCentral |
| Right Caudal Middle Frontal | Right SupraMarginal |
| Right Caudal Middle Frontal | Right Superior Parietal |
| Right Caudal Middle Frontal | Right Inferior Parietal |
| Right ParsOpercularis | Right PostCentral |
| Right ParsOpercularis | Right SupraMarginal |
| Right ParsOpercularis | Right Superior Parietal |
| Right ParsOpercularis | Right Inferior Parietal |
| Right ParsTriangularis | Right ParaCentral Lobule |
| Right ParsTriangularis | Right PostCentral |
| Right ParsTriangularis | Right SupraMarginal |
| Right ParsTriangularis | Right Superior Parietal |
| Right ParsTriangularis | Right Inferior Parietal |
| Right ParsOrbitalis | Right Superior Parietal |
| Right Orbital Frontal | Right SupraMarginal |
| Right Orbital Frontal | Right Superior Parietal |
| Right Orbital Frontal | Right Inferior Parietal |
| Right PreCentral | Right ParaCentral Lobule |
| Right PreCentral | Right SupraMarginal |
| Right PreCentral | Right Superior Parietal |
| Right PreCentral | Right Inferior Parietal |
| Right CA Cingulate | Right Inferior Parietal |

| Left Superior Frontal | Left PreCuneus |
| --- | --- |
| Left Superior Frontal | Left Lingual |
| Left Superior Frontal | Left Lateral Occipital |
| Left Superior Frontal | Right PreCuneus |
| Left Superior Frontal | Right Lateral Occipital |
| Left Rostral Middle Frontal | Right Lateral Occipital |
| Left Caudal Middle Frontal | Left PreCuneus |
| Left Caudal Middle Frontal | Left Lateral Occipital |
| Left Caudal Middle Frontal | Right Lateral Occipital |
| Left ParsOpercularis | Left PreCuneus |
| Left ParsOpercularis | Left Lateral Occipital |
| Left ParsOpercularis | Right Lateral Occipital |
| Left ParsTriangularis | Left PreCuneus |
| Left ParsTriangularis | Left Lingual |
| Left ParsTriangularis | Left Lateral Occipital |
| Left ParsTriangularis | Right PreCuneus |
| Left ParsTriangularis | Right Lingual |
| Left ParsTriangularis | Right Lateral Occipital |
| Left Orbital Frontal | Left PreCuneus |
| Left Orbital Frontal | Left Lateral Occipital |
| Left Orbital Frontal | Right PreCuneus |
| Left Orbital Frontal | Right Lateral Occipital |
| Left Medial Orbital Frontal | Left PreCuneus |
| Left PreCentral | Left PreCuneus |
| Left PreCentral | Left Lateral Occipital |
| Left CA Cingulate | Left PreCuneus |
| Right Superior Frontal | Right PreCuneus |
| Right Superior Frontal | Right Lateral Occipital |
| Right Rostral Middle Frontal | Right Lateral Occipital |
| Right Caudal Middle Frontal | Right PreCuneus |
| Right ParsTriangularis | Right PreCuneus |
| Right ParsTriangularis | Right Lateral Occipital |
| Right Orbital Frontal | Right PreCuneus |
| Right Orbital Frontal | Right Lingual |
| Right Orbital Frontal | Right Lateral Occipital |
| Right PreCentral | Right PreCuneus |
| Right PreCentral | Right Lingual |
| Left Superior Frontal | Left Insula |
| Left Superior Frontal | Right Insula |
| Left Rostral Middle Frontal | Left Insula |
| Left Rostral Middle Frontal | Right Insula |
| Left Caudal Middle Frontal | Left Insula |
| Left ParsOpercularis | Left Insula |
| Left ParsOpercularis | Right Insula |
| Left ParsTriangularis | Left Insula |
| Left ParsTriangularis | Right Insula |
| Left ParsOrbitalis | Left Insula |
| Left Orbital Frontal | Left Insula |
| Left Orbital Frontal | Right Insula |
| Left Frontal Pole | Right Insula |
| Left PreCentral | Left Insula |
| Left PreCentral | Right Insula |
| Right Superior Frontal | Right Insula |
| Right Rostral Middle Frontal | Right Insula |
| Right Caudal Middle Frontal | Right Insula |
| Right ParsOpercularis | Right Insula |
| Right ParsTriangularis | Right Insula |
| Right Orbital Frontal | Right Insula |
| Right PreCentral | Right Insula |
| Right CA Cingulate | Right Insula |
| Left Fusiform | Right ParsOpercularis |
| Left Fusiform | Right Orbital Frontal |
| Left Fusiform | Right RA cingulate |
| Left Superior Temporal | Right Superior Frontal |
| Left Superior Temporal | Right Rostral Middle Frontal |
| Left Superior Temporal | Right Caudal Middle Frontal |

| Left Superior Temporal | Right ParsTriangularis |
| --- | --- |
| Left Superior Temporal | Right PreCentral |
| Left Middle Temporal | Right Superior Frontal |
| Left Middle Temporal | Right Caudal Middle Frontal |
| Left Middle Temporal | Right ParsOpercularis |
| Left Middle Temporal | Right ParsTriangularis |
| Left Middle Temporal | Right Orbital Frontal |
| Left Middle Temporal | Right PreCentral |
| Left Inferior Temporal | Right Superior Frontal |
| Left Inferior Temporal | Right ParsTriangularis |
| Left ParaCentral Lobule | Right Superior Frontal |
| Left ParaCentral Lobule | Right Caudal Middle Frontal |
| Left ParaCentral Lobule | Right ParsOpercularis |
| Left ParaCentral Lobule | Right ParsTriangularis |
| Left ParaCentral Lobule | Right PreCentral |
| Left PostCentral | Right Superior Frontal |
| Left PostCentral | Right Caudal Middle Frontal |
| Left PostCentral | Right ParsOpercularis |
| Left PostCentral | Right ParsTriangularis |
| Left PostCentral | Right Orbital Frontal |
| Left PostCentral | Right PreCentral |
| Left SupraMarginal | Right Superior Frontal |
| Left SupraMarginal | Right Caudal Middle Frontal |
| Left SupraMarginal | Right ParsOpercularis |
| Left SupraMarginal | Right ParsTriangularis |
| Left SupraMarginal | Right Orbital Frontal |
| Left SupraMarginal | Right PreCentral |
| Left Superior Parietal | Right Superior Frontal |
| Left Superior Parietal | Right Caudal Middle Frontal |
| Left Superior Parietal | Right ParsOpercularis |
| Left Superior Parietal | Right ParsTriangularis |
| Left Superior Parietal | Right Orbital Frontal |
| Left Superior Parietal | Right PreCentral |
| Left Inferior Parietal | Right Superior Frontal |
| Left Inferior Parietal | Right Caudal Middle Frontal |
| Left Inferior Parietal | Right ParsOpercularis |
| Left Inferior Parietal | Right ParsTriangularis |
| Left Inferior Parietal | Right Orbital Frontal |
| Left Inferior Parietal | Right PreCentral |
| Left PreCuneus | Right Superior Frontal |
| Left PreCuneus | Right Caudal Middle Frontal |
| Left PreCuneus | Right ParsOpercularis |
| Left PreCuneus | Right ParsTriangularis |
| Left PreCuneus | Right Orbital Frontal |
| Left PreCuneus | Right PreCentral |
| Left Lingual | Right Caudal Middle Frontal |
| Left Lingual | Right ParsOpercularis |
| Left Lingual | Right ParsTriangularis |
| Left Lingual | Right Orbital Frontal |
| Left Lingual | Right PreCentral |
| Left Lateral Occipital | Right Superior Frontal |
| Left Lateral Occipital | Right ParsTriangularis |
| Left Lateral Occipital | Right PreCentral |
| Left Insula | Right Superior Frontal |
| Left Insula | Right Caudal Middle Frontal |
| Left Insula | Right ParsOpercularis |
| Left Insula | Right ParsTriangularis |
| Left Insula | Right Orbital Frontal |
| Left Insula | Right PreCentral |
| Left Entorhinal | Left Temporal Pole |
| Left Entorhinal | Right Entorhinal |
| Left Parahippocampal | Right Parahippocampal |
| Left Temporal Pole | Left Fusiform |
| Left Temporal Pole | Left Middle Temporal |
| Left Temporal Pole | Left Inferior Temporal |
| Left Temporal Pole | Right Temporal Pole |

| Left Temporal Pole | Right Middle Temporal |
| --- | --- |
| Left Temporal Pole | Right Inferior Temporal |
| Left Fusiform | Left Superior Temporal |
| Left Fusiform | Left Middle Temporal |
| Left Fusiform | Left Inferior Temporal |
| Left Fusiform | Left Banks Superior Temporal |
| Left Fusiform | Right Entorhinal |
| Left Fusiform | Right Superior Temporal |
| Left Fusiform | Right Middle Temporal |
| Left Fusiform | Right Inferior Temporal |
| Left Fusiform | Right Banks Superior Temporal |
| Left Superior Temporal | Left Middle Temporal |
| Left Superior Temporal | Left Inferior Temporal |
| Left Superior Temporal | Right Temporal Pole |
| Left Superior Temporal | Right Superior Temporal |
| Left Superior Temporal | Right Middle Temporal |
| Left Superior Temporal | Right Inferior Temporal |
| Left Middle Temporal | Left Inferior Temporal |
| Left Middle Temporal | Left Transverse Temporal |
| Left Middle Temporal | Left Banks Superior Temporal |
| Left Middle Temporal | Right Temporal Pole |
| Left Middle Temporal | Right Fusiform |
| Left Middle Temporal | Right Superior Temporal |
| Left Middle Temporal | Right Middle Temporal |
| Left Middle Temporal | Right Inferior Temporal |
| Left Middle Temporal | Right Banks Superior Temporal |
| Left Inferior Temporal | Right Entorhinal |
| Left Inferior Temporal | Right Parahippocampal |
| Left Inferior Temporal | Right Temporal Pole |
| Left Inferior Temporal | Right Fusiform |
| Left Inferior Temporal | Right Superior Temporal |
| Left Inferior Temporal | Right Middle Temporal |
| Left Inferior Temporal | Right Inferior Temporal |
| Left Inferior Temporal | Right Banks Superior Temporal |
| Left Transverse Temporal | Right Inferior Temporal |
| Right Entorhinal | Right Temporal Pole |
| Right Entorhinal | Right Fusiform |
| Right Entorhinal | Right Middle Temporal |
| Right Entorhinal | Right Inferior Temporal |
| Right Parahippocampal | Right Temporal Pole |
| Right Parahippocampal | Right Superior Temporal |
| Right Parahippocampal | Right Middle Temporal |
| Right Temporal Pole | Right Superior Temporal |
| Right Temporal Pole | Right Middle Temporal |
| Right Temporal Pole | Right Inferior Temporal |
| Right Fusiform | Right Superior Temporal |
| Right Fusiform | Right Middle Temporal |
| Right Fusiform | Right Banks Superior Temporal |
| Right Superior Temporal | Right Middle Temporal |
| Right Superior Temporal | Right Inferior Temporal |
| Right Superior Temporal | Right Transverse Temporal |
| Right Superior Temporal | Right Banks Superior Temporal |
| Right Middle Temporal | Right Inferior Temporal |
| Right Middle Temporal | Right Transverse Temporal |
| Right Middle Temporal | Right Banks Superior Temporal |
| Right Inferior Temporal | Right Banks Superior Temporal |
| Right Transverse Temporal | Right Banks Superior Temporal |
| Left Fusiform | Left SupraMarginal |
| Left Fusiform | Left Inferior Parietal |
| Left Fusiform | Right SupraMarginal |
| Left Fusiform | Right Inferior Parietal |
| Left Superior Temporal | Left PostCentral |
| Left Superior Temporal | Left SupraMarginal |
| Left Superior Temporal | Left Superior Parietal |
| Left Superior Temporal | Left Inferior Parietal |
| Left Superior Temporal | Right ParaCentral Lobule |

| Left Superior Temporal | Right SupraMarginal |
| --- | --- |
| Left Superior Temporal | Right Superior Parietal |
| Left Superior Temporal | Right Inferior Parietal |
| Left Middle Temporal | Left PostCentral |
| Left Middle Temporal | Left SupraMarginal |
| Left Middle Temporal | Left Superior Parietal |
| Left Middle Temporal | Left Inferior Parietal |
| Left Middle Temporal | Right ParaCentral Lobule |
| Left Middle Temporal | Right SupraMarginal |
| Left Middle Temporal | Right Inferior Parietal |
| Left Inferior Temporal | Left SupraMarginal |
| Left Inferior Temporal | Right SupraMarginal |
| Left Inferior Temporal | Right Inferior Parietal |
| Right Temporal Pole | Right SupraMarginal |
| Right Temporal Pole | Right Superior Parietal |
| Right Temporal Pole | Right Inferior Parietal |
| Right Fusiform | Right SupraMarginal |
| Right Fusiform | Right Inferior Parietal |
| Right Superior Temporal | Right PostCentral |
| Right Superior Temporal | Right SupraMarginal |
| Right Superior Temporal | Right Superior Parietal |
| Right Superior Temporal | Right Inferior Parietal |
| Right Middle Temporal | Right SupraMarginal |
| Right Middle Temporal | Right Superior Parietal |
| Right Middle Temporal | Right Inferior Parietal |
| Right Inferior Temporal | Right SupraMarginal |
| Right Inferior Temporal | Right Inferior Parietal |
| Right Banks Superior Temporal | Right SupraMarginal |
| Right Banks Superior Temporal | Right Inferior Parietal |
| Left Fusiform | Left PreCuneus |
| Left Fusiform | Left Lateral Occipital |
| Left Fusiform | Right PreCuneus |
| Left Fusiform | Right Lateral Occipital |
| Left Superior Temporal | Left PreCuneus |
| Left Superior Temporal | Left Lateral Occipital |
| Left Superior Temporal | Right PreCuneus |
| Left Superior Temporal | Right Lateral Occipital |
| Left Middle Temporal | Left PreCuneus |
| Left Middle Temporal | Left Lateral Occipital |
| Left Middle Temporal | Right PreCuneus |
| Left Middle Temporal | Right Lateral Occipital |
| Left Inferior Temporal | Left PreCuneus |
| Left Inferior Temporal | Left Lateral Occipital |
| Left Inferior Temporal | Right PreCuneus |
| Right Entorhinal | Right Lingual |
| Right Temporal Pole | Right PreCuneus |
| Right Superior Temporal | Right PreCuneus |
| Right Superior Temporal | Right Lateral Occipital |
| Right Middle Temporal | Right PreCuneus |
| Right Middle Temporal | Right Lingual |
| Right Middle Temporal | Right Lateral Occipital |
| Right Inferior Temporal | Right PreCuneus |
| Right Inferior Temporal | Right Lateral Occipital |
| Right Banks Superior Temporal | Right PreCuneus |
| Left Fusiform | Right Insula |
| Left Superior Temporal | Right Insula |
| Left Middle Temporal | Right Insula |
| Left Inferior Temporal | Right Insula |
| Left ParaCentral Lobule | Left Superior Temporal |
| Left ParaCentral Lobule | Right Superior Temporal |
| Left ParaCentral Lobule | Right Middle Temporal |
| Left ParaCentral Lobule | Right Inferior Temporal |
| Left ParaCentral Lobule | Right Banks Superior Temporal |
| Left PostCentral | Right Temporal Pole |
| Left PostCentral | Right Superior Temporal |
| Left PostCentral | Right Middle Temporal |

| Left SupraMarginal | Right Fusiform |
| --- | --- |
| Left SupraMarginal | Right Superior Temporal |
| Left SupraMarginal | Right Middle Temporal |
| Left SupraMarginal | Right Inferior Temporal |
| Left SupraMarginal | Right Banks Superior Temporal |
| Left Superior Parietal | Right Temporal Pole |
| Left Superior Parietal | Right Superior Temporal |
| Left Superior Parietal | Right Middle Temporal |
| Left Superior Parietal | Right Inferior Temporal |
| Left Superior Parietal | Right Banks Superior Temporal |
| Left Inferior Parietal | Right Superior Temporal |
| Left Inferior Parietal | Right Middle Temporal |
| Left Inferior Parietal | Right Inferior Temporal |
| Left Inferior Parietal | Right Banks Superior Temporal |
| Right ParaCentral Lobule | Right Fusiform |
| Right ParaCentral Lobule | Right Superior Temporal |
| Right ParaCentral Lobule | Right Middle Temporal |
| Right ParaCentral Lobule | Right Transverse Temporal |
| Right ParaCentral Lobule | Right Banks Superior Temporal |
| Left PreCuneus | Right Temporal Pole |
| Left PreCuneus | Right Superior Temporal |
| Left PreCuneus | Right Middle Temporal |
| Left PreCuneus | Right Inferior Temporal |
| Left PreCuneus | Right Banks Superior Temporal |
| Left Lingual | Right Superior Temporal |
| Left Lingual | Right Inferior Temporal |
| Left Pericalcarine | Right Inferior Temporal |
| Left Cuneus | Right Inferior Temporal |
| Left Lateral Occipital | Right Entorhinal |
| Left Lateral Occipital | Right Parahippocampal |
| Left Lateral Occipital | Right Temporal Pole |
| Left Lateral Occipital | Right Superior Temporal |
| Left Lateral Occipital | Right Middle Temporal |
| Left Lateral Occipital | Right Inferior Temporal |
| Left Lateral Occipital | Right Banks Superior Temporal |
| Left Insula | Left Fusiform |
| Left Insula | Left Superior Temporal |
| Left Insula | Left Middle Temporal |
| Left Insula | Left Inferior Temporal |
| Left Insula | Right Temporal Pole |
| Left Insula | Right Superior Temporal |
| Left Insula | Right Middle Temporal |
| Left Insula | Right Inferior Temporal |
| Right Insula | Right Temporal Pole |
| Right Insula | Right Middle Temporal |
| Left ParaCentral Lobule | Left Posterior Cingulate |
| Left ParaCentral Lobule | Left PostCentral |
| Left ParaCentral Lobule | Left SupraMarginal |
| Left ParaCentral Lobule | Left Superior Parietal |
| Left ParaCentral Lobule | Left Inferior Parietal |
| Left ParaCentral Lobule | Right ParaCentral Lobule |
| Left ParaCentral Lobule | Right SupraMarginal |
| Left ParaCentral Lobule | Right Superior Parietal |
| Left ParaCentral Lobule | Right Inferior Parietal |
| Left Posterior Cingulate | Right Posterior Cingulate |
| Left Isthmus | Right Isthmus |
| Left PostCentral | Left SupraMarginal |
| Left PostCentral | Left Superior Parietal |
| Left PostCentral | Left Inferior Parietal |
| Left PostCentral | Right ParaCentral Lobule |
| Left PostCentral | Right PostCentral |
| Left PostCentral | Right SupraMarginal |
| Left PostCentral | Right Superior Parietal |
| Left PostCentral | Right Inferior Parietal |
| Left SupraMarginal | Left Superior Parietal |
| Left SupraMarginal | Left Inferior Parietal |

| Left SupraMarginal | Right ParaCentral Lobule |
| --- | --- |
| Left SupraMarginal | Right SupraMarginal |
| Left SupraMarginal | Right Superior Parietal |
| Left SupraMarginal | Right Inferior Parietal |
| Left Superior Parietal | Left Inferior Parietal |
| Left Superior Parietal | Right ParaCentral Lobule |
| Left Superior Parietal | Right PostCentral |
| Left Superior Parietal | Right SupraMarginal |
| Left Superior Parietal | Right Superior Parietal |
| Left Superior Parietal | Right Inferior Parietal |
| Left Inferior Parietal | Right ParaCentral Lobule |
| Left Inferior Parietal | Right SupraMarginal |
| Left Inferior Parietal | Right Superior Parietal |
| Left Inferior Parietal | Right Inferior Parietal |
| Right ParaCentral Lobule | Right PostCentral |
| Right ParaCentral Lobule | Right SupraMarginal |
| Right ParaCentral Lobule | Right Superior Parietal |
| Right ParaCentral Lobule | Right Inferior Parietal |
| Right PostCentral | Right Superior Parietal |
| Right SupraMarginal | Right Superior Parietal |
| Right SupraMarginal | Right Inferior Parietal |
| Right Superior Parietal | Right Inferior Parietal |
| Left ParaCentral Lobule | Left PreCuneus |
| Left ParaCentral Lobule | Right PreCuneus |
| Left PostCentral | Left PreCuneus |
| Left PostCentral | Left Lingual |
| Left PostCentral | Left Lateral Occipital |
| Left PostCentral | Right PreCuneus |
| Left PostCentral | Right Lateral Occipital |
| Left SupraMarginal | Left PreCuneus |
| Left SupraMarginal | Left Lateral Occipital |
| Left SupraMarginal | Right PreCuneus |
| Left SupraMarginal | Right Lateral Occipital |
| Left Superior Parietal | Left PreCuneus |
| Left Superior Parietal | Left Lingual |
| Left Superior Parietal | Left Cuneus |
| Left Superior Parietal | Left Lateral Occipital |
| Left Superior Parietal | Right PreCuneus |
| Left Superior Parietal | Right Lingual |
| Left Superior Parietal | Right Lateral Occipital |
| Left Inferior Parietal | Left PreCuneus |
| Left Inferior Parietal | Left Lingual |
| Left Inferior Parietal | Left Lateral Occipital |
| Left Inferior Parietal | Right PreCuneus |
| Left Inferior Parietal | Right Lateral Occipital |
| Right ParaCentral Lobule | Right PreCuneus |
| Right SupraMarginal | Right PreCuneus |
| Right SupraMarginal | Right Lateral Occipital |
| Right Superior Parietal | Right PreCuneus |
| Right Superior Parietal | Right Cuneus |
| Right Superior Parietal | Right Lateral Occipital |
| Right Inferior Parietal | Right PreCuneus |
| Right Inferior Parietal | Right Lingual |
| Right Inferior Parietal | Right Lateral Occipital |
| Left ParaCentral Lobule | Right Insula |
| Left PostCentral | Right Insula |
| Left SupraMarginal | Right Insula |
| Left Superior Parietal | Right Insula |
| Left Inferior Parietal | Right Insula |
| Right ParaCentral Lobule | Right Insula |
| Right Posterior Cingulate | Right Insula |
| Left PreCuneus | Right ParaCentral Lobule |
| Left PreCuneus | Right SupraMarginal |
| Left PreCuneus | Right Superior Parietal |
| Left PreCuneus | Right Inferior Parietal |
| Left Lingual | Right PostCentral |

| Left Lingual | Right SupraMarginal |
| --- | --- |
| Left Lingual | Right Superior Parietal |
| Left Lingual | Right Inferior Parietal |
| Left Pericalcarine | Right SupraMarginal |
| Left Cuneus | Right Superior Parietal |
| Left Cuneus | Right Inferior Parietal |
| Left Lateral Occipital | Right ParaCentral Lobule |
| Left Lateral Occipital | Right SupraMarginal |
| Left Lateral Occipital | Right Inferior Parietal |
| Left Insula | Left PostCentral |
| Left Insula | Left SupraMarginal |
| Left Insula | Left Superior Parietal |
| Left Insula | Left Inferior Parietal |
| Left Insula | Right ParaCentral Lobule |
| Left Insula | Right SupraMarginal |
| Left Insula | Right Inferior Parietal |
| Right Insula | Right PostCentral |
| Right Insula | Right SupraMarginal |
| Right Insula | Right Inferior Parietal |
| Left PreCuneus | Left Lingual |
| Left PreCuneus | Left Lateral Occipital |
| Left PreCuneus | Right PreCuneus |
| Left PreCuneus | Right Lingual |
| Left PreCuneus | Right Lateral Occipital |
| Left Lingual | Left Lateral Occipital |
| Left Lingual | Right PreCuneus |
| Left Lingual | Right Lingual |
| Left Cuneus | Right Cuneus |
| Left Lateral Occipital | Right PreCuneus |
| Left Lateral Occipital | Right Lingual |
| Left Lateral Occipital | Right Lateral Occipital |
| Right Lingual | Right Lateral Occipital |
| Left PreCuneus | Right Insula |
| Left Lateral Occipital | Right Insula |
| Left Insula | Left Lingual |
| Left Insula | Left Lateral Occipital |
| Left Insula | Right PreCuneus |
| Left Insula | Right Lateral Occipital |
| Right Insula | Right PreCuneus |
| Right Insula | Right Lateral Occipital |
| Left Insula | Right Insula |

**Table S21.** Cross-cortical correlations in the adolescence-onset conduct disorder (AO-CD) group showing overlap between the significant inter-regional correlations identified using structural covariance methods in the Cambridge and Southampton samples *when not* including the covariates in the statistical models

| Left Superior Frontal | Left Rostral Middle Frontal |
| --- | --- |
| Left Superior Frontal | Left Caudal Middle Frontal |
| Left Superior Frontal | Left PreCentral |
| Left Superior Frontal | Right Superior Frontal |
| Left Superior Frontal | Right Rostral Middle Frontal |
| Left Superior Frontal | Right Caudal Middle Frontal |
| Left Superior Frontal | Right PreCentral |
| Left Rostral Middle Frontal | Left Caudal Middle Frontal |
| Left Rostral Middle Frontal | Right Superior Frontal |
| Left Rostral Middle Frontal | Right Rostral Middle Frontal |
| Left Caudal Middle Frontal | Left PreCentral |
| Left Caudal Middle Frontal | Right Superior Frontal |
| Left Caudal Middle Frontal | Right Rostral Middle Frontal |
| Left Caudal Middle Frontal | Right Caudal Middle Frontal |
| Left Caudal Middle Frontal | Right PreCentral |
| Left PreCentral | Right Caudal Middle Frontal |
| Left PreCentral | Right PreCentral |
| Right Superior Frontal | Right Rostral Middle Frontal |
| Right Superior Frontal | Right Caudal Middle Frontal |

| Right Rostral Middle Frontal | Right Caudal Middle Frontal |
| --- | --- |
| Right Rostral Middle Frontal | Right ParsTriangularis |
| Left ParsTriangularis | Left Superior Temporal |
| Right Superior Frontal | Right Middle Temporal |
| Left Superior Frontal | Left PostCentral |
| Left Superior Frontal | Left SupraMarginal |
| Left Superior Frontal | Left Superior Parietal |
| Left Superior Frontal | Left Inferior Parietal |
| Left Superior Frontal | Right ParaCentral Lobule |
| Left Superior Frontal | Right SupraMarginal |
| Left Superior Frontal | Right Superior Parietal |
| Left Superior Frontal | Right Inferior Parietal |
| Left Rostral Middle Frontal | Left SupraMarginal |
| Left Caudal Middle Frontal | Left SupraMarginal |
| Left Caudal Middle Frontal | Left Inferior Parietal |
| Left Caudal Middle Frontal | Right SupraMarginal |
| Left PreCentral | Left PostCentral |
| Left PreCentral | Left SupraMarginal |
| Right Superior Frontal | Right Isthmus |
| Right Superior Frontal | Right SupraMarginal |
| Right Superior Frontal | Right Superior Parietal |
| Right Superior Frontal | Right Inferior Parietal |
| Right Caudal Middle Frontal | Right SupraMarginal |
| Left Superior Frontal | Left PreCuneus |
| Left Superior Frontal | Right PreCuneus |
| Left Caudal Middle Frontal | Right PreCuneus |
| Left PreCentral | Left Lateral Occipital |
| Left PreCentral | Right Lateral Occipital |
| Right Superior Frontal | Right PreCuneus |
| Right Caudal Middle Frontal | Right PreCuneus |
| Right ParsOpercularis | Right Insula |
| Left PostCentral | Right Caudal Middle Frontal |
| Left SupraMarginal | Right Superior Frontal |
| Left SupraMarginal | Right Caudal Middle Frontal |
| Left SupraMarginal | Right PreCentral |
| Left Inferior Parietal | Right Superior Frontal |
| Left Inferior Parietal | Right Caudal Middle Frontal |
| Left PreCuneus | Right Superior Frontal |
| Left Entorhinal | Left Temporal Pole |
| Left Fusiform | Right Fusiform |
| Left Fusiform | Right Inferior Temporal |
| Left Superior Temporal | Right Superior Temporal |
| Left Transverse Temporal | Right Superior Temporal |
| Left Transverse Temporal | Right Transverse Temporal |
| Right Fusiform | Right Middle Temporal |
| Right Fusiform | Right Inferior Temporal |
| Right Fusiform | Right SupraMarginal |
| Right Fusiform | Right Superior Parietal |
| Right Fusiform | Right Inferior Parietal |
| Right Middle Temporal | Right Inferior Parietal |
| Right Fusiform | Right PreCuneus |
| Right Fusiform | Right Lingual |
| Right Fusiform | Right Lateral Occipital |
| Right Banks Superior Temporal | Right PreCuneus |
| Left Inferior Parietal | Right Middle Temporal |
| Left PreCuneus | Right Fusiform |
| Left Lateral Occipital | Right Fusiform |
| Left Insula | Left Superior Temporal |
| Right Insula | Right Superior Temporal |
| Left ParaCentral Lobule | Left PostCentral |
| Left ParaCentral Lobule | Left SupraMarginal |
| Left ParaCentral Lobule | Left Superior Parietal |
| Left ParaCentral Lobule | Left Inferior Parietal |
| Left ParaCentral Lobule | Right ParaCentral Lobule |
| Left ParaCentral Lobule | Right Superior Parietal |
| Left ParaCentral Lobule | Right Inferior Parietal |

| Left Posterior Cingulate | Left Isthmus |
| --- | --- |
| Left PostCentral | Left SupraMarginal |
| Left PostCentral | Left Superior Parietal |
| Left PostCentral | Left Inferior Parietal |
| Left PostCentral | Right ParaCentral Lobule |
| Left PostCentral | Right PostCentral |
| Left PostCentral | Right SupraMarginal |
| Left PostCentral | Right Superior Parietal |
| Left PostCentral | Right Inferior Parietal |
| Left SupraMarginal | Left Superior Parietal |
| Left SupraMarginal | Left Inferior Parietal |
| Left SupraMarginal | Right SupraMarginal |
| Left SupraMarginal | Right Superior Parietal |
| Left SupraMarginal | Right Inferior Parietal |
| Left Superior Parietal | Left Inferior Parietal |
| Left Superior Parietal | Right Superior Parietal |
| Left Superior Parietal | Right Inferior Parietal |
| Left Inferior Parietal | Right SupraMarginal |
| Left Inferior Parietal | Right Superior Parietal |
| Left Inferior Parietal | Right Inferior Parietal |
| Right ParaCentral Lobule | Right Superior Parietal |
| Right ParaCentral Lobule | Right Inferior Parietal |
| Right PostCentral | Right Superior Parietal |
| Right SupraMarginal | Right Inferior Parietal |
| Right Superior Parietal | Right Inferior Parietal |
| Left ParaCentral Lobule | Left PreCuneus |
| Left PostCentral | Left PreCuneus |
| Left PostCentral | Left Cuneus |
| Left PostCentral | Left Lateral Occipital |
| Left PostCentral | Right PreCuneus |
| Left PostCentral | Right Lateral Occipital |
| Left SupraMarginal | Left PreCuneus |
| Left SupraMarginal | Left Lateral Occipital |
| Left SupraMarginal | Right PreCuneus |
| Left Superior Parietal | Left PreCuneus |
| Left Superior Parietal | Right PreCuneus |
| Left Inferior Parietal | Left PreCuneus |
| Left Inferior Parietal | Left Lingual |
| Left Inferior Parietal | Left Lateral Occipital |
| Left Inferior Parietal | Right PreCuneus |
| Right ParaCentral Lobule | Right PreCuneus |
| Right ParaCentral Lobule | Right Lingual |
| Right ParaCentral Lobule | Right Lateral Occipital |
| Right PostCentral | Right Lateral Occipital |
| Right SupraMarginal | Right PreCuneus |
| Right Superior Parietal | Right PreCuneus |
| Right Superior Parietal | Right Lingual |
| Right Superior Parietal | Right Cuneus |
| Right Superior Parietal | Right Lateral Occipital |
| Right Inferior Parietal | Right PreCuneus |
| Right Inferior Parietal | Right Lateral Occipital |
| Left PreCuneus | Right ParaCentral Lobule |
| Left PreCuneus | Right SupraMarginal |
| Left PreCuneus | Right Superior Parietal |
| Left PreCuneus | Right Inferior Parietal |
| Left Lingual | Right Superior Parietal |
| Left Cuneus | Right SupraMarginal |
| Left Cuneus | Right Superior Parietal |
| Left Lateral Occipital | Right ParaCentral Lobule |
| Left Lateral Occipital | Right Superior Parietal |
| Left Lateral Occipital | Right Inferior Parietal |
| Left PreCuneus | Left Lateral Occipital |
| Left PreCuneus | Right PreCuneus |
| Left PreCuneus | Right Cuneus |
| Left PreCuneus | Right Lateral Occipital |
| Left Lingual | Left Pericalcarine |

| Left Lingual | Left Cuneus |
| --- | --- |
| Left Lingual | Left Lateral Occipital |
| Left Lingual | Right PreCuneus |
| Left Lingual | Right Lingual |
| Left Lingual | Right Lateral Occipital |
| Left Cuneus | Left Lateral Occipital |
| Left Cuneus | Right Lingual |
| Left Cuneus | Right Cuneus |
| Left Cuneus | Right Lateral Occipital |
| Left Lateral Occipital | Right PreCuneus |
| Left Lateral Occipital | Right Lingual |
| Left Lateral Occipital | Right Cuneus |
| Left Lateral Occipital | Right Lateral Occipital |
| Right PreCuneus | Right Cuneus |
| Right PreCuneus | Right Lateral Occipital |
| Right Lingual | Right Cuneus |
| Right Lingual | Right Lateral Occipital |
| Right Pericalcarine | Right Cuneus |
| Left Insula | Right Insula |
